# Requirement of transcription-coupled nucleotide excision repair for the removal of a specific type of oxidatively induced DNA damage

**DOI:** 10.1093/nar/gkad256

**Published:** 2023-04-07

**Authors:** Leen Sarmini, Mohammed Meabed, Eirini Emmanouil, George Atsaves, Elena Robeska, Bolesław T Karwowski, Anna Campalans, Thanasis Gimisis, Andriy Khobta

**Affiliations:** Institute of Nutritional Sciences, Friedrich Schiller University Jena, Jena 07743, Germany; Institute of Nutritional Sciences, Friedrich Schiller University Jena, Jena 07743, Germany; Department of Chemistry, National and Kapodistrian University of Athens, Athens 15771, Greece; Department of Chemistry, National and Kapodistrian University of Athens, Athens 15771, Greece; Université Paris-Saclay, CEA/IBFJ/IRCM. UMR Stabilité Génétique Cellules Souches et Radiations, Fontenay-aux-Roses, F-92265, France; Université de Paris Cité, CEA/IBFJ/IRCM. UMR Stabilité Génétique Cellules Souches et Radiations, Fontenay-aux-Roses, F-92265, France; DNA Damage Laboratory of Food Science Department, Faculty of Pharmacy, Medical University of Lodz, Lodz 90-151, Poland; Université Paris-Saclay, CEA/IBFJ/IRCM. UMR Stabilité Génétique Cellules Souches et Radiations, Fontenay-aux-Roses, F-92265, France; Université de Paris Cité, CEA/IBFJ/IRCM. UMR Stabilité Génétique Cellules Souches et Radiations, Fontenay-aux-Roses, F-92265, France; Department of Chemistry, National and Kapodistrian University of Athens, Athens 15771, Greece; Institute of Nutritional Sciences, Friedrich Schiller University Jena, Jena 07743, Germany

## Abstract

Accumulation of DNA damage resulting from reactive oxygen species was proposed to cause neurological and degenerative disease in patients, deficient in nucleotide excision repair (NER) or its transcription-coupled subpathway (TC-NER). Here, we assessed the requirement of TC-NER for the repair of specific types of oxidatively generated DNA modifications. We incorporated synthetic 5′,8-cyclo-2′-deoxypurine nucleotides (cyclo-dA, cyclo-dG) and thymine glycol (Tg) into an *EGFP* reporter gene to measure transcription-blocking potentials of these modifications in human cells. Using null mutants, we further identified the relevant DNA repair components by a host cell reactivation approach. The results indicated that NTHL1-initiated base excision repair is by far the most efficient pathway for Tg. Moreover, Tg was efficiently bypassed during transcription, which effectively rules out TC-NER as an alternative repair mechanism. In a sharp contrast, both cyclopurine lesions robustly blocked transcription and were repaired by NER, wherein the specific TC-NER components *CSB/ERCC6* and *CSA/ERCC8* were as essential as *XPA*. Instead, repair of classical NER substrates, cyclobutane pyrimidine dimer and *N*-(deoxyguanosin-8-yl)-2-acetylaminofluorene, occurred even when TC-NER was disrupted. The strict requirement of TC-NER highlights cyclo-dA and cyclo-dG as candidate damage types, accountable for cytotoxic and degenerative responses in individuals affected by genetic defects in this pathway.

## INTRODUCTION

The nucleotide excision repair (NER) pathway is of a paramount importance for protection from potentially mutagenic DNA damage inflicted by environmental carcinogens. Hereditary defects in NER genes typically translate to sensitivity to UV irradiation and increased tumour incidence already at young age, which has established their causal role in the cancer predisposition syndrome xeroderma pigmentosum (XP) (1). Two subpathways—the global genome (GG-NER) and transcription-coupled (TC-NER)—have been recognised within the NER system. The discovery of their molecular details led to understanding that genetic defects in the components of GG-NER and TC-NER cause different disease phenotypes, as extensively reviewed elsewhere (2–4). Thus, defects in GG-NER genes (such as *XPC* and *XPE*/*DDB2*) confer cancer predisposition, since accumulating DNA damage causes increased mutation rates in replicating cells. In contrast, loss-of-function mutations in TC-NER components (most prominently *CSA*/*ERCC8* and *CSB*/*ERCC6*) do not lead to cancer but rather to diseases characterised by pronounced neurological features and segmental degeneration, such as Cockayne syndrome (CS) and an even more severe cerebro-oculo-facio-skeletal syndrome (COFS). The molecular defect beneath the pathogenesis of CS is commonly defined as incapacity of cells to recover transcription after exposure to genotoxic agents, however the nature of the responsible DNA damage remains unknown.

Blockage of transcribing RNA polymerase complexes at unrepaired damage sites is highly cytotoxic (5,6). In healthy cells, the capacity of DNA modifications to block elongating RNA polymerase II provides a versatile substrate recognition mechanism for TC-NER, however this very mechanism is impaired in CS (7–13). In contrast, GG-NER components primarily recognise structural properties of damaged DNA duplex itself (14–21). Since GG-NER and TC-NER use fundamentally different damage recognition principles, their substrate spectra may not fully overlap. Accordingly, some types of DNA modifications strictly require TC-NER for their removal (22). Others are efficiently, or even preferentially, recognised by GG-NER (23). Based on these considerations, the range of candidate DNA lesions causing neuropathology in CS can be narrowed down to transcription blocking NER substrates, which cannot undergo repair by other mechanisms, including GG-NER. Moreover, as central nervous system is protected from exogenous genotoxic substances by the blood brain barrier, the damage in question should likely arise from an endogenous source.

Since neuronal function heavily relies on aerobic metabolism, it has been proposed that reactive oxygen species arising as by-products during respiration could be responsible for the critical DNA damage (24). Several types of oxidatively generated DNA lesions were suggested as candidates. In the first line, these include intrastrand crosslinks, such as 5′,8-cyclopurine-2′-deoxynucleosides (cyclo-dA, cyclo-dG), whose second covalent linkage to deoxyribose makes them inherently resistant to base excision repair (BER), unlike other common DNA oxidation products (24,25). Biochemical evidence has characterised both cyclo-dA cyclo-dG as fairly good NER substrates (26–28), and their transcription blocking potentials towards human RNA polymerase II have been reported (29). Another candidate is thymine glycol (Tg), which is a reported NER substrate as well (30). Both types of cyclopurine lesions and thymine glycol can be generated in DNA by reactions with hydroxyl radicals, which arise through radiolytic decay of water or by Fenton-like reactions of hydrogen peroxide (31–34). The latter is ubiquitously generated in cells as an intermediate detoxification product of superoxide radical anions released during various redox reactions, including the mitochondrial electron transport (35).

We previously developed procedures for efficient incorporation of various types of synthetic DNA modifications into plasmid-borne reporter genes (36,37) and proposed a reliable methodology for quantification of the reporter expression in transfected human cells (38). The combination of these techniques provided a means to both determine the impairment of transcription by structurally defined types of DNA damage and, based on cells’ capacities to recuperate the expression, measure the specific repair efficiencies (22,23). In the present study we used patient-derived cell lines, carrying genetic defects in the specific NER genes, as well as newly generated knockout cell models, to define specific contributions of GG-NER and TC-NER to the repair of synthetic cyclo-dA, cyclo-dG and Tg lesions.

## MATERIALS AND METHODS

### Synthesis of the protected phosphoramidite of 2′α-fluorothymine glycol

The synthesis of the protected phosphoramidite of 2′α-fluorothymine glycol is reported in detail in Supplementary Information (Supplementary Figure S1). In brief, the benzyl protected precursor 2′α-F-thymidine was synthesised through a minor modification of the reported synthesis (39). 5′-*O*-(4,4′-Dimethoxytrityl)-5,6-dihydro-5,6-di[(tert-butyl)dimethoxysilyl]-2′α-fluorothymidine was obtained as an 8:1 mixture of two inseparable diastereomers. After comparing with the bibliographic spectra (40), the major diastereomer was assigned to (5*R*,6*S*) and the minor to (5*S*,6*R*). The last step of the synthesis was the formation of the (2-cyanoethyl)-*N*,*N*-diisopropylphosphoramidite. Both the reaction and the chromatographic purification of the crude mixture took place under strictly anhydrous and degassed conditions. The desired product was obtained as an 8:1 mixture of the (5*R*,6*S*) and (5*S*,6*R*) diastereomers.

### Patient derived cell lines

All patient-derived cell lines were purchased from the NIGMS Human Genetic Cell Repository, Coriell Institute for Medical Research (Camden, New Jersey, USA). Immortalised skin fibroblasts of the following NER complementation groups were used: XP-A (GM04312), CS-B (GM16095), CS-A (GM16094), and XP-C (GM15983). The reference repair-proficient cell lines were MRC-5VA1 (AG10076) and the cell line derived from GM04312 by complementation with human XPA cDNA (GM15876) (23,41).

### Targeted disruption of *CSA/ERCC8*, *DDB2/XPE* and *NTHL1* genes in HeLa cells

Cells used for gene knockout were monoclonal descendants of HeLa cervical carcinoma cell line (German Collection of Microorganisms and Cell Cultures No. ACC 57). Each gene was targeted by a pair of sgRNAs expressed from the pX330-SpCas9-HF1 vector (Addgene, Watertown, MA, USA) (42), designed to cut on both sides of exon sequences critical for the protein functions. Potentially suitable sgRNA sequences were selected using the CHOPCHOP web tool (43). Clones isolated by single cell sorting of transfected cells were expanded in 96-well plates, screened by PCR, and validated by Western blotting, as described previously (44). In the case of CSA knockout, the TC-NER deficiency was independently validated by the host-cell reactivation assay using synthetic 3-(deoxyguanosin-*N^2^*-yl)-2-acetylaminofluorene (dG(*N^2^*)-AAF) as a specific TC-NER substrate (22). Comprehensive gene targeting results as well as sgRNA and PCR primer sequences are reported in Supplementary Information (Supplementary Figures S2–S4; Supplementary Table S1).

### Generation of the reporter constructs carrying specific synthetic DNA modifications

**Figure 1.**
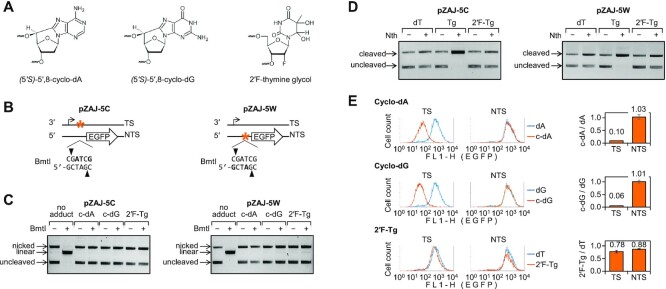
Analysis of transcription blocking capacities of cyclo-dA, cyclo-dG and 2′F-Tg modifications in human XP-A (GM04312) cells. (**A**) Structures of synthetic DNA modifications incorporated into vector DNA. (**B**) Schemes indicating positions of the incorporated modifications (marked bold in the sequence) within the BmtI site. Cyclo-dA was incorporated opposite to T, cyclo-dG opposite to C, and 2′F- or deoxy-Tg opposite to A. A pair of vectors with inverted target sequence was used to selectively incorporate synthetic oligonucleotides with modifications into either the transcribed (TS) or non-transcribed DNA strand (NTS). (**C**) Verification of efficient incorporation of synthetic DNA modifications by BmtI cleavage inhibition. (**D**) Verification of resistance of 2′F-Tg incorporated into vector DNA to endonuclease III (*Nth*) compared to the 2′-deoxy-Tg substrate. The reference ‘no adduct’ constructs were obtained by ligation of the modification-free synthetic oligonucleotide. (**E**) Quantification of EGFP expression in XP-A (GM04312) cells by flow cytometry. EGFP fluorescence distribution plots show overlaid expression data for reporter constructs containing the specified modifications (amber) along with the modification-free reference constructs (blue). Bar charts on the right show quantification of the EGFP expression. Median fluorescence values were normalised relative to the modification-free constructs and mean relative expression calculated for three to six independent experiments (±SD).

The procedure for efficient site-specific incorporation of synthetic DNA modifications (Figure 1A) into the 5′-untranslated region of the enhanced green fluorescent protein (EGFP) gene was described previously (22). Expression vectors pZAJ-5C of pZAJ-5W were cut at the available tandem sites with the Nb.BsrDI nicking endonuclease (NEB GmbH, Frankfurt am Main, Germany) in either the transcribed DNA strand (pZAJ-5C) or the opposite strand (pZAJ-5W) to generate 18-nt gaps. Synthetic oligonucleotides containing the specified modifications or unmodified control strands (all HPLC-purified and validated by mass spectrometry) were annealed and ligated to obtain fully double stranded covalently closed reporter constructs for transfection.

Synthetic 18-mer oligonucleotides 5′-CATTGCTTCGCT[cyclo-dA]GCACG and 5′-CATTGCTTC[cyclo-dG]CTAGCACG containing 5′*S* isomers of the specified cyclopurine modifications were purchased from Kaneka Eurogentec S.A. (Seraing, Belgium). For specific incorporation of 5′*R* and 5′*S* cyclo-dA stereoisomers, phosphoramidites of the respective 2′-deoxyribonucleosides were synthesised according to the previously described protocol (45), and the oligonucleotides were produced in the Bioorganic Chemistry Department of the Polish Academy of Science (Lodz, Poland), as described previously (46). The oligonucleotides 5′-CATTGCTTCGC[Tg]AGCACG and 5′-CATTGCTTCGC[2′F-Tg]AGCACG containing 2′-deoxy thymine glycol (Tg) or its 2′α-fluorinated analog (2′F-Tg) were produced at Metabion GmbH (Planegg, Germany). The oligonucleotide 5′-CATTGC[TT dimer]CGCTAGCACG containing a *cis*-*syn* cyclobutane pyrimidine dimer (TT dimer) was from TriLink BioTechnologies (San Diego, CA). Oligonucleotides containing dG(*N^2^*)-AAF and N-(deoxyguanosin-8-yl)-2-acetylaminofluorene (dG(C8)-AAF) at the same position as cyclo-dG were described previously (22). The reference unmodified deoxyribo-oligonucleotide 5′-CATTGCTTCGCTAGCACG was from Eurofins Genomics (Ebersberg, Germany).

### Verification of lesion incorporation into reporter vectors

Correct incorporation of synthetic oligonucleotides was monitored by inhibition of ligation by unphosphorylated synthetic strands and, additionally, by formation of covalently closed circular DNA in the presence of polynucleotide kinase, as described previously (37). Incorporation of thymine dimer was further specifically verified by incision with T4 endonuclease V (NEB), as described previously (23). The presence of Tg lesion was verified by an analogous reaction with endonuclease III (NEB), as described previously (37). The presence of cyclo-dA and cyclo-dG adducts was confirmed by inhibition of cleavage by BmtI restriction endonuclease (NEB) at the specific 5′-GCTAGC sequence (Figure 1B and C). The presence of dG(*N^2^*)-AAF and dG(C8)-AAF was verified, as described previously (22), by the inhibition of NheI (NEB) cleavage (Supplementary Figure S5).

### EGFP expression and host-cell reactivation assays

Transfection of cells with reporter constructs carrying the specified synthetic modifications were performed exactly as described previously for other types of DNA lesions (23). Constructs prepared with oligonucleotides with and without modifications were always transfected and analysed in parallel. They were mixed with equal amounts of pDsRed-Monomer-N1 (Clontech, Saint-Germainen-Laye, France) as a marker allowing selective measurement of transfected cells during flow cytometry. EGFP and DsRed expression was analysed in cells fixed 24 h post-transfection using FACSCalibur™ and the CellQuest™ Pro software (Beckton Dickinson GmbH, Heidelberg, Germany), as described previously (23). Where indicated, CytoFLEX instrument with the CytExpert software (Beckman Coulter GmbH, Krefeld, Germany) was used for analysis with quantitatively identical results. After exclusion of fragments and aggregates, transfected cells were selectively gated by the same DsRed expression threshold to generate the EGFP fluorescence distribution plots shown in figures. Median EGFP fluorescence values (*M*) of the obtained distributions were used to calculate relative expression as *M*(construct with modification):*M*(reference construct without modification).

## RESULTS

### Cyclopurine lesions but not thymine glycol inhibit transcription by the RNA polymerase-mediated mechanism

Transcription-blocking potentials of nucleobase modifications, can be determined by their incorporation into the transcribed strand of a reporter gene and measurement of the gene expression in a NER-deficient cell model, such as cells derived from XP-A patients (22,27,29). Due to the presence of the covalent 5′,8 bonds (Figure 1A), cyclo-dA and cyclo-dG adducts are intrinsically resistant to excision by DNA *N*-glycosylases of the BER pathway and, thereby, cannot be removed from vector DNA transfected to XP-A cells. This is different for Tg lesion, which can be removed from DNA by at least two different DNA *N*-glycosylases (47–51). Therefore, to assess transcription blocking capacity of Tg, we used its BER-resistant 2′α-fluorinated analog (Figure 1A), which was synthesised specifically for this purpose (Supplementary Figure S1).

We used a pair of EGFP expression vectors containing an inverted sequence in the 5′-untranslated region (22) to incorporate cyclo-dA, cyclo-dG or 2′F-Tg into either the transcribed DNA strand or the non-transcribed strand. An efficient and correct incorporation of synthetic oligonucleotides carrying the specified modifications was confirmed by analytical ligation reactions (Supplementary Figure S6). By design, all modifications were placed within the recognition sequence of the BmtI restriction endonuclease. Thereby, the presence of modifications could additionally be validated by prevention of BmtI cleavage (Figure 1B and C). Finally, we compared susceptibilities of synthetic Tg and 2′F-Tg to endonuclease III, which has both DNA *N*-glycosylase and AP-lyase activities. At the saturating concentration resulting in complete cleavage at 2′-deoxyTg, only a non-specific nicking activity (at the levels equivalent to the respective dT controls) was detectable in the plasmid DNA containing the 2′α-fluorinated analog (Figure 1D and Supplementary Figure S7). The results thus indicate that 2′F-Tg is BER-resistant, as intended by design.

To assess transcription blocking capacities of cyclo-dA, cyclo-dG, and Tg, the expression of constructs containing the specified modifications in the transcribed DNA strand was measured in NER-deficient XP-A cells. Based on the EGFP fluorescence levels, 2′F-Tg caused a very minor inhibition of the EGFP expression, which can hardly be regarded as biologically significant (Figure 1E). In a sharp contrast, the presence of a single cyclopurine modification led to a 10-fold (cyclo-dA, *P* = 7.8 × 10^−12^, heteroscedastic Student's *t*-test) or a 17-fold (cyclo-dG, *P* = 2.4 × 10^−12^) reduction of the gene expression, relative to the modification-free control (Figure 1E, ‘TS’). These values reflect exceptionally high transcription-blocking potencies, alike those of cyclobutane pyrimidine dimers or acetylaminofluorene adducts (22). It is important to note that EGFP expression remained unaffected when the same cyclopurine adducts were placed in the opposite DNA strand (Figure 1E, ‘NTS’). The strict confinement of the effects of cyclo-dA and cyclo-dG to the transcribed strand provides a proof that impaired gene expression results from interaction of the adducts with components of the transcribing RNA polymerase complex as the only strand-specific biological component.

### Removal of transcription-blocking cyclopurine lesions requires XP-A

**Figure 2.**
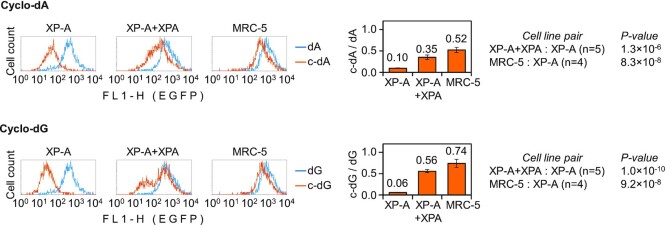
Recovery of gene expression from the transcription block caused by synthetic cyclo-dA or cyclo-dG modifications after complementation with XPA. Expression of the same constructs was analysed in parallel in the XP-A cells (with and without XPA complementation) and MRC-5 as a NER-proficient control. Fluorescence distribution plots of a representative experiment and quantification of the EGFP expression relative to the modification-free construct (mean of *n*≥ 4 independent experiments, ± SD; *P*-values calculated by Student's two-tailed *t*-test).

To investigate whether transcription-blocking cyclo-dA and cyclo-dG can be repaired by reconstitution of NER capacity, we performed the gene expression recovery analyses in XP-A cells complemented by expression of human XPA cDNA (Figure 2). We observed a very significant reactivation of both cyclo-dA (from 9.51 ± 0.74% to 35.40 ± 5.47%) and cyclo-dG (from 5.50 ± 0.61% to 55.88 ± 3.72%) reporter constructs in the XP-A corrected cell lines, indicating that both adducts undergo repair, which requires a functional *XPA* gene. Even higher levels of reporter reactivation were documented in fully NER-proficient MRC-5 cells. Besides, we have noticed that the recovery of gene expression was more pronounced in the case of cyclo-dG (74.18 ± 9.61%) compared to the cyclo-dA construct (52.33 ± 5.86%), suggesting that cyclo-dG is repaired more efficiently (Figure 2).

### TC-NER is strictly required for the repair of cyclopurine lesions

**Figure 3.**
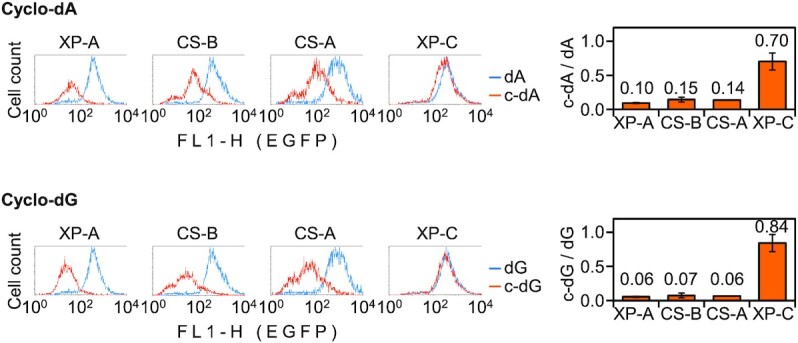
Repair capacities of CS-B (GM16095), CS-A (GM16094), and XP-C (GM15983) cells towards transcription-blocking cyclo-dA or cyclo-dG in the transcribed DNA strand. XP-A cells were transfected with the same constructs as a reference for a total NER defect. Fluorescence distribution plots of a representative experiment and quantification of the EGFP expression relative to the modification-free construct (mean of *n*≥ 3 independent experiments, ±SD).

The requirement of XPA implied that repair of cyclo-dA and cyclo-dG takes place by the NER mechanism. Based on this, we further assessed the requirements of TC-NER and GG-NER for the removal of both types of cyclopurine lesions from transcribed DNA, using cell lines derived from patients with defects in the respective damage recognition components (Figure 3). We found that CS-A and CS-B cells were deficient in the reporter gene reactivation to the same degree as the XP-A cell line, indicating that GG-NER, although unaffected in CS cells, fails to recognise cyclo-dA and cyclo-dG. This notion was further supported by high repair capacities of XP-C cells, which indicated that both cyclo-dA and cyclo-dG in the transcribed DNA strand are recognised very efficiently by the remaining TC-NER.

**Figure 4.**
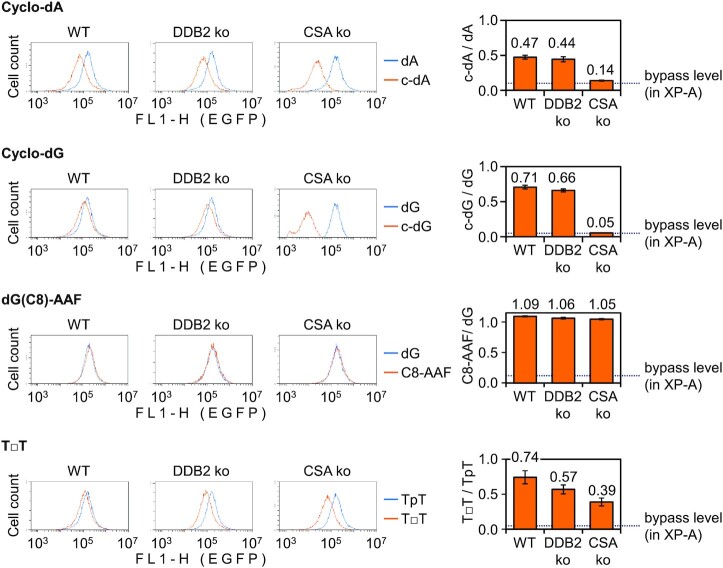
Repair capacities of HeLa (‘WT’) and the derived knockout cell lines (‘CSA ko’ and ‘DDB2 ko’) towards transcription-blocking cyclo-dA or cyclo-dG in the transcribed DNA strand, analysed by the CytoFLEX flow cytometer. Host-cell reactivation of reporter constructs containing synthetic dG(C8)-AAF and TT dimer (T□T), as NER substrates processed by both TC-NER and GG-NER, is shown for comparison. Fluorescence distribution plots of a representative experiment and quantification of the EGFP expression relative to the modification-free construct (mean of *n*= 4 independent experiments, ±SD).

Since differences observed between the patients-derived cell lines could potentially be influenced by the individual genetic backgrounds, we also generated isogenic cell lines with specific defects in either TC-NER or GG-NER pathways by disruption of *CSA*/*ERCC8* or *DDB2*/*XPE* genes in HeLa cells (Supplementary Figures S2 and S3). Reporter reactivation analyses in these cell lines revealed a severe repair defect in the HeLa-derived TC-NER deficient *CSA* knockout cell line (Figure 4). Indeed, *CSA* knockout cells showed relative expression values of 13.96 ± 0.73% (compared to 47.43 ± 2.67% in the parental HeLa cell line) for cyclo-dA and 5.21 ± 0.07% for cyclo-dG (compared to 70.78 ± 2.63% in HeLa). Since the expression of cyclo-dA constructs in the *CSA* knockout cell line was barely above the levels attributable to transcriptional bypass in totally NER-deficient XP-A cells (9.51 ± 0.74%) (Figure 3), we conclude that CSA is of a major importance for cyclo-dA repair. We further conclude that CSA is essential for cyclo-dG repair, since the reporter expression was negligible in the absence of CSA and did not exceed the level measured in the XP-A cells (5.5 ± 0.62%).

In contrast, *DDB2* knockout cells retained essentially full repair capacities for both cyclo-dA (44.47 ± 3.62% relative EGFP expression compared to 47.43 ± 2.67% in the parental cell line) and cyclo-dG (66.03 ± 2.21% compared to 70.78 ± 2.63% in the parental cell line). Collectively, the results in the isogenic *DDB2* and *CSA* knockout cell lines exactly recapitulated the TC-NER and GG-NER phenotypes of the patients-derived cell lines (Figure 3), thereby corroborating the conclusions that repair of cyclopurine nucleotides in the transcribed strand of the reporter gene is dominated by TC-NER whereas GG-NER contribution to total repair capacity is negligible.

### GG-NER of non-cyclopurine lesions is excellently detectable in the transcribed DNA strand

Considering a peculiar chromatin structure of the transfected plasmid vectors (38) and, especially, the fact that the reporter-based repair assay is confined to the transcribed DNA strand, we questioned whether GG-NER would be as readily detected within the present experimental system as TC-NER. We therefore next determined repair efficiencies of two more synthetic adducts, a *cis*-*syn* cyclobutane pyrimidine dimer (TT dimer) and an N-(deoxyguanosin-8-yl)-2-acetylaminofluorene (dG(C8)-AAF), both of which have very strong transcription-blocking potentials (22) and are well characterised substrates for both TC-NER (9,52,53) and GG-NER (14,54,55). In XP-A cells, both dG(C8)-AAF and TT dimer caused a nearly absolute abolition of transcription, very much alike cyclo-dA and cyclo-dG (Supplementary Figures S5 and S8). Remarkably, however, expression of the dG(C8)-AAF construct was reactivated to the level of the adduct-free control not only in HeLa cells but also in the DDB2 and CSA knockouts. Such a complete reactivation should indicate that either GG-NER or TC-NER alone is sufficient for the dG(C8)-AAF repair, and that GG-NER is well detectable in the transcribed DNA strand.

For the construct containing TT dimer, relative expression recovered to 38.97 ± 5.60% under the *CSA* knockout conditions (compared to 74.27 ± 9.30% in the parental cell line). Thus, despite the well recognised fact that TT dimers are repaired by TC-NER far more efficiently than by GG-NER (14,54–56), the impact of *CSA* knockout on the capacity of cells to repair TT dimer was by far milder than observed for cyclopurine adducts (Figure 4). Combined, the results indicate that GG-NER provides a significant backup repair activity for the removal of TT-dimers and even more for dG(C8)-AAF. On the other hand, GG-NER is largely or entirely ineffective against cyclo-dA or cyclo-dG.

### GG-NER makes a minimal or no contribution to the repair of both cyclo-dA diastereoisomers

**Figure 5.**
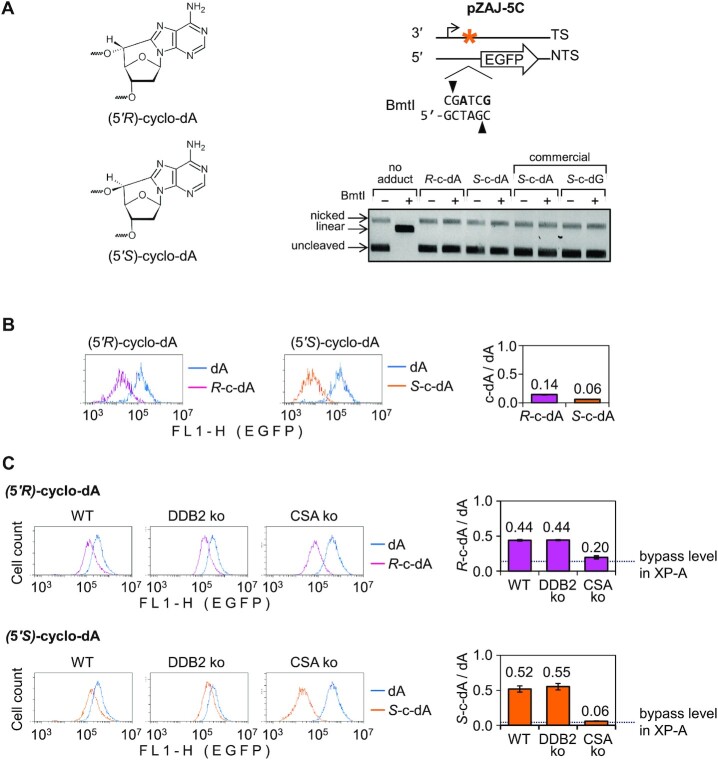
Transcription blockage by 5′*R* and 5′*S* cyclo-dA diastereoisomers and the impact of DDB2 and CSA knockouts on the repair. (**A**) Chemical structures of (5′*R*)- and (5′*S*)-cyclo-dA and verification of efficient incorporation of synthesised oligonucleotides containing the specific cyclo-dA diastereoisomers into the pZAJ-5C transcribed strand by the BmtI inhibition. Constructs obtained by ligation of unmodified oligonucleotide (‘no adduct’) or the commercial cyclo-dA/cyclo-dG oligonucleotides (as in Figure 1) are shown for comparison. (**B**) Transcription blockage in the XP-A (GM04312) cell line. Fluorescence distribution plots of a representative experiment and quantification of the EGFP expression relative to the modification-free construct (mean of *n*= 4 independent experiments, ±SD). (**C**) Host-cell reactivation in HeLa (‘WT’) and the derived knockout cell lines (‘CSA ko’ and ‘DDB2 ko’). (B and C) EGFP expression in cells transfected with constructs harbouring (5′*R*)- and (5′*S*)-cyclo-dA was analysed by the CytoFLEX flow cytometer. Fluorescence distribution plots of representative experiments and quantification of the EGFP expression relative to the modification-free construct are shown (mean of *n*= 4 independent experiments, ±SD).

According to the manufacturer's communication (Kaneka Eurogentec S.A.), oligonucleotide strands used for construction of the reporters described above were synthesised with 5′*S* diastereoisomers of the cyclopurine 2′-deoxyribonucleosides. Thereby our previous conclusions about TC-NER requirements for the repair were limited to 5′*S*-cyclo-dG and 5′*S*-cyclo-dA. It was reported in the literature that 5′*R* stereoisomers of cyclo-dA or cyclo-dG are slightly better NER substrates under cell-free conditions than their 5′*S* counterparts (28). These observations should refer specifically to GG-NER activity because the assay used by the authors does not detect TC-NER. We therefore questioned whether GG-NER significantly contributes to repair of 5′*R*-cyclo-dA in the cellular context. Cyclo-dA was chosen, because data obtained with its 5′*S* stereoisomer did not entirely exclude a minor contribution of GG-NER to the repair. We performed stereoselective synthesis of 5′*R*- and 5′*S*-cyclo-dA phosphoramidites, synthesized the respective oligonucleotides, as specified under Materials and Methods, and incorporated them into the transcribed DNA strand of the reporter EGFP gene (Figure 5A).

Expression analyses in the XP-A cell line, showed that both cyclo-dA stereoisomers inflicted strong transcription blockage, 5′*R*-cyclo-dA being bypasses with a slightly higher efficiency (14.3 ± 0.58% EGFP expression relative to the adduct-free construct) than the 5′*S* counterpart (5.93 ± 0.28%) (Figure 5B). In fully NER-proficient HeLa cells the expression recovered to the levels of 44 ± 0.94% (5′*R*) and 51.81 ± 4.45% (5′*S*), suggesting that 5′*S*-cyclo-dA may be repaired with a slightly higher efficiency. The reporter reactivation rates were not significantly affected by the DDB2 knockout (Figure 5C). In contrast, CSA knockout led to strong decrease of the EGFP expression in comparison to the parental cell line, which was highly significant for both cyclo-dA stereoisomers (*P* = 9.0 × 10^−7^ for 5′*R*- and *P* = 8.5 × 10^−7^ for 5′*S*-cyclo-dA, heteroscedastic Student's *t*-test). The expression levels in the absence of CSA were as low as in the XP-A cell line (for 5′*S* isomer) or slightly above the XP-A value (for 5′*R*). The results thus indicate that TC-NER is by far more efficient than GG-NER for both cyclo-dA stereoisomers and, within the accuracy margins of the host-cell reactivation assay, the only NER subpathway contributing to the removal of 5′*S*-cyclo-dA from the transcribed DNA strand. Also for 5′*R*-cyclo-dA, TC-NER is clearly the primary repair mechanism, although a minor contribution of GG-NER cannot be entirely excluded for this stereoisomer.

### Thymine glycol is predominantly processed by BER

**Figure 6.**
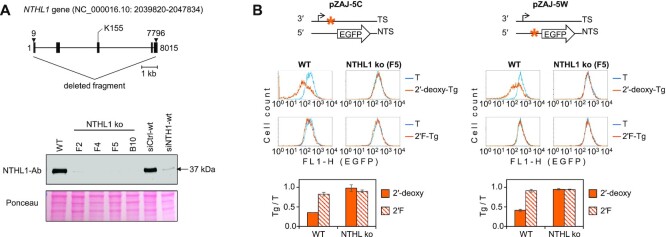
Impact of Tg (2′-deoxy and 2′F) lesions on the EFGP gene expression in HeLa (‘WT’) and the derived *NTHL1* knockout cell line (‘NTHL1 ko’). (**A**) Validation of the NTHL1 knockout in selected clones by Western blotting with EPR15930 antibody (Abcam, ab191413). The last two lanes show parental ‘WT’ cell line after transfection with short interfering RNA against NTHL1 (siNTHL1) or unspecific short RNA (siCtrl). (**B**) Representative EGFP fluorescence distribution plots of cells transfected with constructs containing 2′-deoxy-Tg or 2′F-Tg in the specified DNA strands (TS or NTS) and the relative EGFP expression values (mean of *n*= 3 independent experiments, ±SD).

Excision of DNA damage can lead to impairment of transcription via conflicts between its components and the repair proteins or the strand-cleaved intermediates (9,57). During NER, such conflicts are probably prevented by the multiprotein transcription factor IIH (TFIIH), which has essential functions in both DNA repair reactions and transcription. Accordingly, active NER leads to reactivation of the reporter gene expression by removing transcription-blocking adducts, as reported previously (22) and seen in the experiments described above (Figure 2). It needs to be noted that BER, in contrast to NER, typically has a different outcome for the reporter gene expression. BER starts from recognition and release of a modified nucleobase by the specific DNA *N*-glycosylase and proceeds with cleavage of the abasic deoxyribose remnant by the endo-/exonuclease APE1. Such a mode of excision commonly leads to transcriptional silencing, as reported for several BER substrates (36,44,58–60). So, in many cell lines, including HeLa, BER activity can be deduced based on a diminished gene expression even if the primary DNA modification does not impede the RNA polymerase progression as such (36,59). Returning to the question about the primary repair mechanism of non-transcription-blocking Tg, we therefore further compared the expression of reporter constructs containing BER-resistant 2′F-Tg and its physiological 2′-deoxy counterpart in HeLa cells. Moreover, we examined the role of NTHL1—one of the DNA glycosylases known to recognise Tg—by disrupting the *NTHL1* gene (Figure 6A).

As expected from previous experiments in the XP-A cell line (Figure 1E), 2′F-Tg did not cause an appreciable decrease of EGFP expression also in HeLa cells (Figure 6B). In contrast, a strong decrease of gene expression was caused by 2′-deoxy-Tg in either the transcribed or the non-transcribed DNA strand. The absence of strand specificity indicated that the decrease of transcription did not require a direct interaction of elongating RNA polymerase with primary DNA modification. Moreover, the fact that such an effect was not caused by 2′F-Tg strongly suggested its dependence on BER. We therefore disrupted the *NTHL*1 gene in HeLa cells (Supplementary Figure S4), which fully reversed the inhibitory effect of 2′-deoxy-Tg (Figure 6). The expression levels of constructs carrying 2′-deoxy- or 2′F-Tg were no longer different in the NTHL1 knockout cells, which implies that ablation of NTHL1 is equivalent to total BER deficiency. Combined, our findings imply that Tg is primarily processed by BER. Although the fraction of Tg lesions excised by BER cannot be determined exactly, based on more than a 2-fold drop in the gene expression levels (35.33 ± 0.37% residual expression for Tg in the transcribed strand, *P* = 1.07 × 10^−5^; 41.35 ± 2.62% in the opposite strand, *P* = 6.66 × 10^−4^) we conclude that BER dominates even in fully NER-proficient HeLa cells.

## DISCUSSION

Potential diversity of repair mechanisms of Tg in mammalian cells remained the matter of debate despite characterisation of NTHL1 and NEIL1 as the main specific DNA glycosylases (47–49). Due to its non-planar structure, Tg causes a significant destabilisation of DNA double helix (61,62) and, at least under specific circumstances, can also cause blockage or pausing of various RNA polymerases (63–66). Based on these properties, Tg is regarded by many as a potential TC-NER substrate. However, such a view is hard to reconcile with the observation that mammalian RNA polymerase II efficiently bypasses Tg in the template DNA strand under cell-free conditions (67). Several attempts to demonstrate TC-NER of Tg in cells were undertaken, but all the reports were subsequently disclosed as invalid, leaving the question open (https://www.science.org/doi/full/10.1126/science.300.5626.1657b; https://aacrjournals.org/cancerres/article/63/13/3846/510149/Retraction; https://www.sciencedirect.com/science/article/pii/S1568786402002367). In our opinion, discrepancies between the previous publications could be explained by uncontrolled BER activity, which would generate apyrimidinic lesion at the Tg site. This is reminiscent of another common oxidative product in DNA, 8-oxo-7,8-dihydroguanine (8-oxoG). Unless cleaved by the respective DNA glycosylase OGG1, 8-oxoG does not block transcription to any significant degree (36). Nonetheless, evidence suggests that TC-NER contributes to the repair, likely at the level of a post-excision BER intermediate (68,69). With the BER-resistant Tg analog, we now could ascertain that the lesion is efficiently bypassed during transcription, since there was no strand-specific effect of 2′F-Tg on the reporter gene expression levels in NER-deficient cells (Figure 1). Since initiation of TC-NER requires transcription blockage (70), our results rule this pathway out as a conceivable repair mechanism for Tg. Still, the results do not formally exclude a possibility of GG-NER. A strand cleavage pattern characteristic for NER was previously reported in biochemically reconstituted repair reactions of DNA containing synthetic Tg (30). However, biological relevance of either GG-NER or a NEIL1-mediated BER appears to be low in the cellular context, as demonstrated by different outcomes of 2′-deoxy-Tg in the isogenic NTHL1-proficient versus knockout cell lines (Figure 6). Our results imply that NTHL1 recognises Tg in transcribed DNA in a strand-independent manner and processes it far more efficiently than any potentially concurrent mechanism.

Cyclo-dA and cyclo-dG were previously characterised as NER substrates (26,28). Elevated levels of cyclopurine lesions were reported in primary XP-C keratinocytes and fibroblasts (71) as well as in CS-A and CS-B cells (72), however neither the causality nor the relative contributions of GG-NER and TC-NER to the repair in cells was established in these studies. Biochemical reconstitution of TC-NER is exceedingly difficult; however, poor transcriptional bypass of cyclopurine lesions under cell-free conditions suggested a possibility of their recognition by TC-NER (29). Accordingly, analyses in NER-deficient rodent and human cells confirmed that both lesions strongly decreased the reporter gene expression rates (27,29), which was attributed partly to blockage and partly to erroneous bypass during transcription (29,73). In NER-proficient 293T cells, reactivation rates of the reporter constructs were approximately 30% and about one third of the reactivation capacity was lost after treatment with short interfering RNA against CSB, suggesting that the repair is partly CSB-dependent (29).

To obtain more conclusive information about repair pathways of cyclopurine nucleotides, we enhanced several parameters compared to the experimental procedures used in previous studies. Firstly, we used an improved design of reporter constructs carrying synthetic cyclo-dA and cyclo-dG. Both modifications were incorporated selectively into either the template or the non-template DNA strand. By this, the impairment of gene expression could be assigned to direct interactions of cyclo-dA and cyclo-dG with transcribing RNA polymerase complexes (Figure 1). Of note, the incorporation sites were chosen within the untranslated gene region to eliminate potential effects of transcriptional mutagenesis on the EGFP expression levels. Secondly, we refined the procedure for quantitative measurement of transcription blockage and recovery by recording the distribution of the EGFP expression over the whole population of individual cells, visualised by co-expression of a transfection marker. Finally, to strictly assign the repair of cyclo-dA and cyclo-dG to the specific NER subpathway, we applied cell lines with genetically defined and phenotypically confirmed total defects in the specific GG-NER and TC-NER components, including isogenic cell pairs generated by gene complementation and knockout. Our results provided unequivocal evidence that disruption of the central GG-NER genes *XPC* and *XPE/DDB2* does not affect the repair capacities of cells, whereas deletions of either *CSA* or *CSB* genes have equally severe consequences, manifested as total repair defects (Figures 3 and 4). These two lines of evidence independently indicate that TC-NER is the only relevant pathway for cyclo-dA and cyclo-dG repair.

As a potential limitation of the used methodology and of transfection-based DNA repair assays in general, it is necessary to consider that chromatin folding of vector DNA is not identical to nuclear chromatin, where the access of DNA repair factors could be limited by nucleosomes or the higher-order structures. However, in our opinion, it is unlikely that apparent absence of GG-NER of cyclopurine lesions in the transiently transfected gene is caused by a restricted access of the repair components to damage sites in DNA, since a less condensed chromatin structure should rather have an opposite effect. It is remarkable that two other well-characterised NER substrates—cyclobutane TT dimer and, especially, the dG(C8)-AAF adduct—display different balance between GG-NER and TC-NER than cyclo-dA and cyclo-dG. Thus GG-NER alone was sufficient for full repair of dG(C8)-AAF, as measured by the host-cell reactivation assay. TT dimer is a relatively poor GG-NER substrate and has long served as paradigmatic type of DNA damage for TC-NER studies (14,54–56). Nonetheless, we observed significant levels of its repair in the absence of CSA (Figure 4) or CSB (22), which indicates that even the lowest GG-NER levels were readily detectable at our assay settings. Consequently, the absent repair activity in CS-B, CS-A (Figure 3), and HeLa-derived CSA knockout (Figures 4 and 5) cell models means that the remaining GG-NER pathway does not process 5′*S* stereoisomers of cyclopurine nucleotides at all. This conclusion is reinforced by re-evaluation of published quantitative data from biochemical reconstitution of human GG-NER. Indeed, although cyclopurine nucleotides are processed by NER, their excision efficiencies are 40 to 150 times lower compared to a *cis-*platin adduct as a canonical reference substrate (26). Moreover, 5′*S* stereoisomers of both cyclo-dA and cyclo-dG lesions destabilise the DNA double helix to lesser degrees than the 5′*R* counterparts and, accordingly, are even poorer GG-NER substrates (28). The cumulative evidence thus corroborates the conclusion that cellular GG-NER capacity is insufficient to warrant a biologically meaningful repair of 5′*S* stereoisomers of cyclopurine nucleotides.

**Figure 7.**
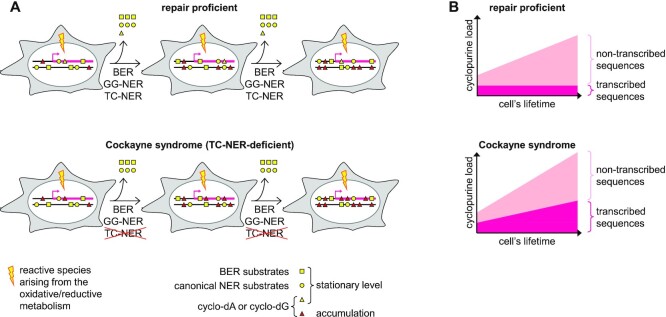
Model predicting dynamics of DNA lesions over the lifetime of a cell under constant exposure to endogenously arising DNA damaging agents (represented with a lightning bolt). Different shapes represent structurally different modification types, which are recognised by different DNA repair pathways. The levels in transcribed DNA strands of active genes (pink strings with broken arrows) are compared to the rest of the genome DNA. (**A**) In fully repair proficient cells, BER and GG-NER substrates (squares and circles) remain at constant levels on average in the genome, since their generation and repair rates are equal under physiological conditions (when repair capacities are not oversaturated). In contrast, TC-NER substrates (yellow triangles) remain at a steady level only in transcribed DNA strands of active genes, where the repair is efficient. The very same lesion types will accumulate all over the non-transcribed DNA sequences (red triangles on the black strings), but their burden can be tolerated, particularly in non-replicating cell types, as far as functionally critical genome regions remain unaffected. This is different in Cockayne syndrome, where TC-NER deficiency will cause the damage to accumulate also in transcribed segments of the genome, where the tolerance threshold is low. (**B**) Expected dynamics of accumulation of cyclopurine DNA lesions (whose repair explicitly requires TC-NER) in transcribed strands of the actively expressed genes (hot pink profiles) and in the rest of the genome (a lighter pink) in the repair proficient versus Cockayne syndrome cells.

Mutations in genes involved in TC-NER commonly result in developmental phenotypes, including early cessation of growth, microcephaly, multiple neurodegenerative features, and a greatly reduced life expectancy. Most affected in CS are neural tissues in the brain that are characterised by high levels of oxidative metabolism, high transcription rates and a slow or absent cell division (4,74). Even under physiologically low rates of damage generation, the deficit of a suitable repair mechanism in the absence of replication should lead to irreversible accumulation of modifications in DNA of such cells. If both GG-NER and TC-NER are impaired, multiple types of lesions would accumulate, leading to XP neurological disease (2). If only TC-NER is absent, only DNA modifications lacking structural determinants for efficient recognition by GG-NER would accumulate, leading to CS (Figure 7). Being refractory to GG-NER and strongly transcription blocking, cyclopurine lesions match the criteria of such cytotoxic DNA lesions.

## DATA AVAILABILITY

CHOPCHOP is a web tool for selecting target sites for CRISPR/Cas9, CRISPR/Cpf1, CRISPR/Cas13 or NICKASE/TALEN-directed mutagenesis (https://chopchop.cbu.uib.no/).

## Supplementary Material

gkad256_Supplemental_File
