# Supplementary material for: Requirement of transcription-coupled nucleotide excision repair for the removal of a specific type of oxidatively induced DNA damage
Source: Nucleic Acids Res. 2023 Apr 7;51(10):4982–94. doi: 10.1093/nar/gkad256 (PMC10250225; doi:10.1093/nar/gkad256)
Supplement: gkad256_Supplemental_File [file gkad256_supplemental_file.pdf]

Supplementary information

Requirement of transcription-coupled nucleotide excision repair for the removal of oxidatively induced DNA damage (by Leen Sarmini et al.)

Supplementary Figures and Table *pages 2 – 10*

Supplementary Figure S1. The synthesis procedure of the 2′F-Tg phosphoramidite.

Supplementary Figure S2. CSA knockout in HeLa cells by CRISPR-Cas9.

Supplementary Figure S3. DDB2 knockout in HeLa cells by CRISPR-Cas9.

Supplementary Figure S4. NTHL1 knockout in HeLa cells by CRISPR-Cas9.

Supplementary Figure S5. Generation and characterisation of reporter constructs harbouring synthetic dG(N^2)-AAF and dG(C8)-AAF adducts.

Supplementary Figure S6. Generation of reporter constructs harbouring synthetic cyclopurine adducts (c-dA or c-dG) or thymine glycol (Tg or 2′F-Tg) in the 5′-untranslated region of the EGFP gene.

Supplementary Figure S7. Quantification of the endonuclease III (Nth) nicking activities towards the reporter constructs containing synthetic 2′-deoxyTg (Tg), its 2′α-fluorinated analog (2′F-Tg), or thymine (T) under the conditions of full cleavage of the Tg substrate.

Supplementary Figure S8. Demonstration of transcription blockage by synthetic cis-syn cyclobutane TT dimer (T□T) in the transcribed DNA strand of the EGFP gene.

Supplementary Table S1. Positions and nucleotide sequences of sgRNA pairs used for CRISPR-Cas9 editing of the specified gene loci and the list of PCR primers used to screen single cell clones for the presence of specific deletions.

Extented Data: Synthesis Conditions and Products Analysis *pages 11 – 47*

Conditions for the synthesis of the 2′F-Tg phosphoramidite

NMR spectra of intermediates and of the final protected 2′F-Tg phosphoramidite (Extended Data 1−33)

Supplementary References *page 48*

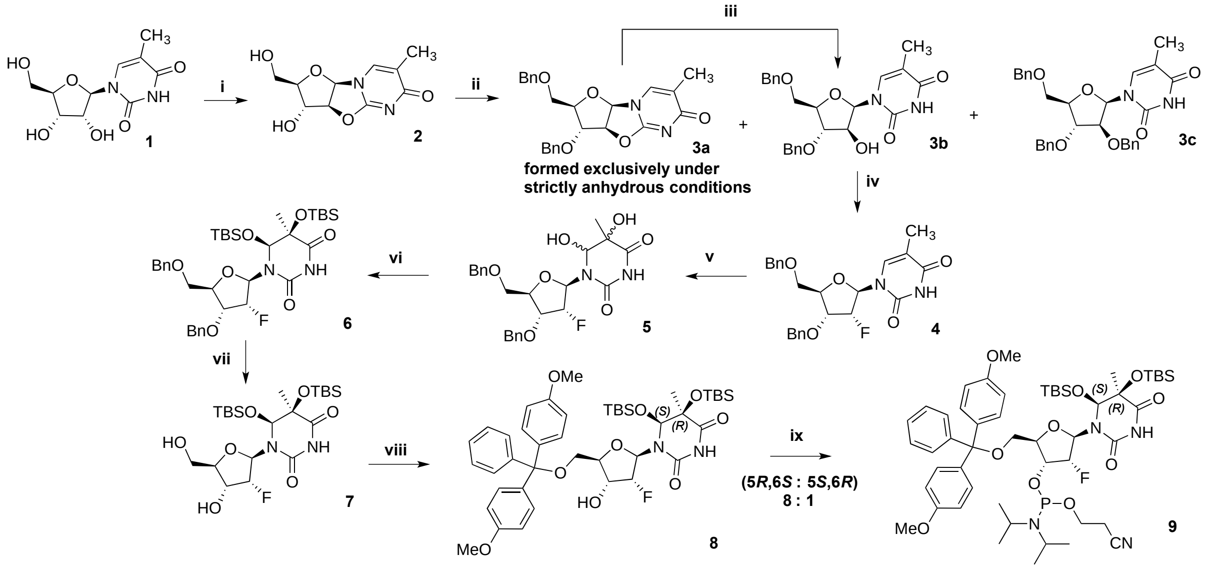

Supplementary Figure S1. Synthesis of 2′F-Tg phosphoramidite. 2,2′-Anhydrothymidine (**2**) and its 3′,5′-dibenzyl derivative were prepared from 5-methyluridine, based on a described procedure (1), with the following improvements and precautions. The main difference was that **2** was isolated by dissolving the reaction mixture in water, washing with diethyl ether to remove the excess of diphenyl carbonate, then concentrating and recrystallizing from MeOH to give the pure product in 86% yield. Similarly, the following benzylation of **2** produced exclusively 3′,5′-dibenzyl-*O*-2,2′-anhydrothymidine (**3a**), only when the reaction was performed under strictly anhydrous conditions. Even traces of humidity, led to a separable mixture of three products and specifically **3a** (22%), together with 3′,5′-di-*O*-benzyl-β-D-arabinofuranosylthymine (**3b**, 45%) and 2′,3′,5′-tri-*O*-benzyl-β-D-arabinofuranosylthymine (**3c**, 30%). Therefore, special cautions were taken to ensure that all glassware was flame-dried under an inert atmosphere and 2,2′-anhydrothymidine (**2**) was dried prior to use by evaporation (3X) of toluene solutions of **2**, followed by overnight drying under vacuum in an oven. 3′,5′-Di-*O*-benzyl-2,2′-anhydrothymidine (**3a**) could be converted to 3′,5′-di-*O*-benzyl-β-D-arabinofuranosylthymine (**3b**) by hydrolysis with K_2CO_3 in MeOH by following the literature procedure (1). The fluorination of **3b** was accomplished with DAST as the fluorinating agent (2), giving the fluorinated nucleoside **4** in 80% yield. Oxidation of the fluorinated derivative **4** by OsO4 produced an inseparable mixture of diastereomers **5**, in high yield (98%) and purity, without the need for chromatographic purification. In the ^{19}F-NMR spectrum, two major peaks, in a 6:1 ratio, were observed, together with minor peaks, indicating the presence of all four possible diastereomers. The 5,6-diol **5** was then protected with the TBDMS group compound **6** and was isolated after purification, in 76% yield, as an inseparable 6:1 mixture of two diastereomers. The removal of the benzyl protecting groups was performed by catalytic hydrogenation (Pd/C), within a 4 h reaction. The desired product **7** was obtained in 96% yield, as an inseparable 10:1 mixture of two diastereomers. It was followed by selective protection of the 5′-hydroxyl with the DMT-group, producing in 75% yield, after purification, 5′-*O*-(4,4′-dimethoxytrityl)-5,6-dihydro-5,6-di[(tert-butyl)dimethoxysilyl]-2′α-fluorothymidine (**8**), described in the literature (3), as an 8:1 mixture of two inseparable diastereomers. After comparing with the published spectra, the major diastereomer was assigned to (5*R*,6*S*) and the minor to (5*S*,6*R*). The last step of the synthesis was the formation of the (2-cyanoethyl)-*N*,*N*-diisopropylphosphoramidite **9**. Both the reaction and the chromatographic purification of the crude mixture took place under strictly anhydrous and degassed conditions. The desired product was obtained as an 8:1 mixture of diastereomers **9** in 67% yield. In the ^{19}F-NMR spectrum of this final target, the fluorine signals split to a 1:1 pair of doublets, due to coupling with the inserted proximal racemic phosphoramidite group. The conditions were: (**i**) $(PhO)_2CO$, $NaHCO_3$, DMF, reflux, 3 h, 82%; (**ii**) BnBr, NaH, DMF, H_2O traces, 0 ºC– r.t., 22% (**3a**), 45% (**3b**), 30% (**3c**); (**iii**) K_2CO_3, MeOH; (**iv**) DAST, pyridine, 0 ºC to refluc, 3h, 80%; (**v**) (a) OsO_4, pyridine, r.t., 2 h (b) $NaHSO_3$, pyridine, H_2O, 12 h, 98%; (**vi**) TBDMSCl, imidazole, dry DMF, 48 h, r.t., 76%; (**vii**) H_2 (1 atm), 10% Pd/C, EtOH, r.t., 4 h, 96 %; (**viii**) DMTCl, DMAP, dry pyridine, r.t., 16 h, 75 %; (**ix**) $(NCCH_2CH_2O)P(N^iPr_2)Cl$, DIPEA, dry DCM, r.t., 3 h, 67 %. Detailed experimental procedures are described in the annexed Extended Data.

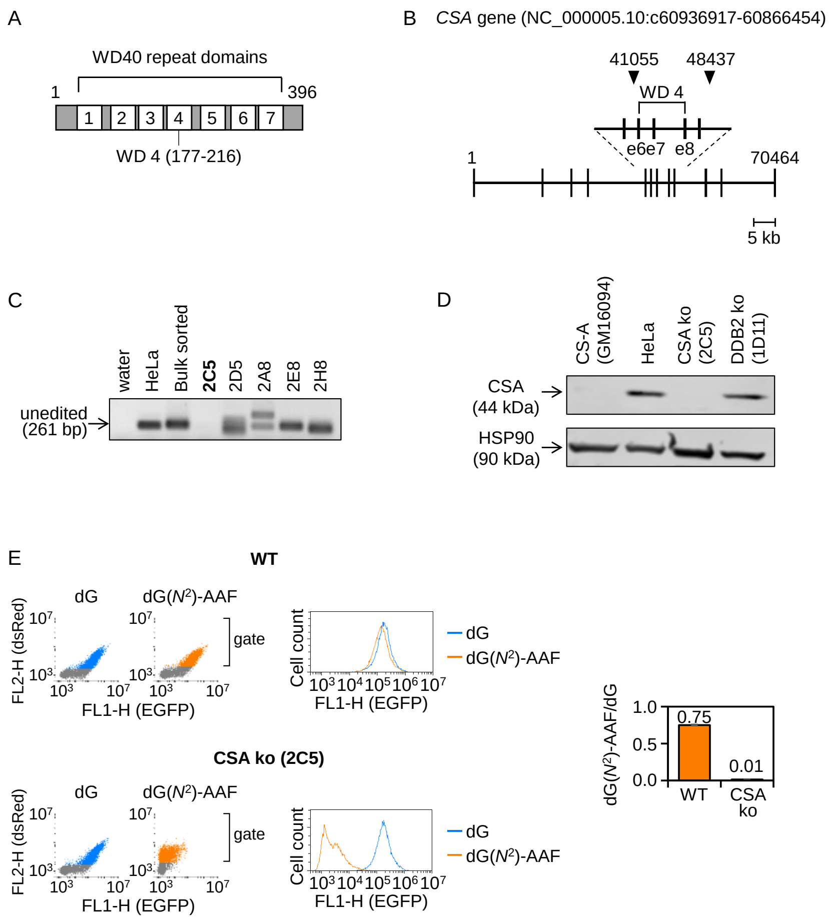

Supplementary Figure S2. CSA knockout in HeLa cells by CRISPR-Cas9.

(A) Depiction of the human 396-amino acid CSA protein with seven WD40 repeat domains (numbered boxes). WD40 repeat domain 4 (WD 4) is critical for the interaction with DDB1.

(B) *CSA* gene disruption scheme. A pair of sgRNAs (arrowheads) was used to delete the fragment including the WD 4-coding exons 6, 7, and 8 (bracket). SgRNA sequences are reported in Supplementary Table S1.

(C) Demonstration of deletion of the targeted *CSA* fragment in the 2C5 clone (bold typeface) chosen from 52 clones isolated by single cell sorting and screened by PCR. A primer pair flanking the upstream sgRNA binding site (Supplementary Table 1) was used to amplify the native (unedited) *CSA* allele.

(D) Validation of CSA knockout in the 2C5 clone by Western blotting with EPR9237 rabbit monoclonal antibody (Abcam, ab137033). Mouse monoclonal antibody to HSP90 (Santa Cruz Biotechnology #sc-13119) was used as an equal loading control.

(E) Validation of the TC-NER-deficient phenotype of the CSA knockout clone. Host cell reactivation assay was carried out using pZAJ-5C construct harboring synthetic dG(N^2)-AAF adduct, whose repair strictly requires TC-NER (4). Parental HeLa cells (WT) were analysed in parallel as a TC-NER-proficient control. Fluorescence distribution plots of transfected cells (representative experiment) and quantification of the recovered fraction of the EGFP expression (dG(N^2)-AAF/dG) in duplicate samples transfected with the same constructs (mean ± SD).

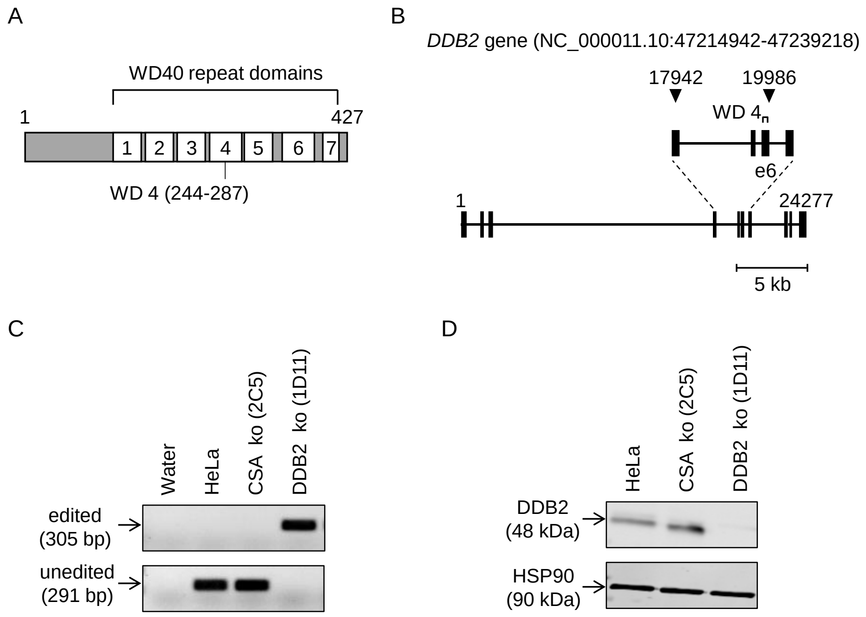

Supplementary Figure S3. DDB2 knockout in HeLa cells by CRISPR-Cas9.

(A) Scheme of the human DDB2 protein containing seven WD40 repeat domains (numbered boxes). The fourth WD40 repeat domain (WD 4) comprises the DWD box required for the DDB1 binding.

(B) *DDB2* gene targeting using a pair of sgRNAs (arrowheads) flanking the region encompassing the exons 4, 5, and 6 (magnified in the cartoon). The exon 6 contains the entire WD 4 coding sequence (bracket). SgRNA sequences are reported in Supplementary Table S1.

(C) Demonstration of the *DDB2* exon 6 deletion in two selected (of 30 screened) knockout clones (1D11 and 2C6) isolated by single cell sorting. Parental HeLa cells and the CSA knockout clone 2C5 (Supplementary Figure S2) were analyzed in parallel. One primer pair was designed to specifically amplify the rearranged (edited) *DDB2* gene fragment; another pair flanking the upstream sgRNA target site was used to amplify the native (unedited) *DDB2* fragment (Supplementary Table S1).

(D) Western blotting with the XPE/DDB2 rabbit polyclonal antibody (R&D Systems #AF3297). All cell extracts display a faint non-specific band slightly above the DDB2 level but there is no detectable DDB2 band in the knockout clones (1D11 and 2C6). Mouse monoclonal antibody to HSP90 (Santa Cruz Biotechnology #sc-13119) was used as an equal loading control.

A

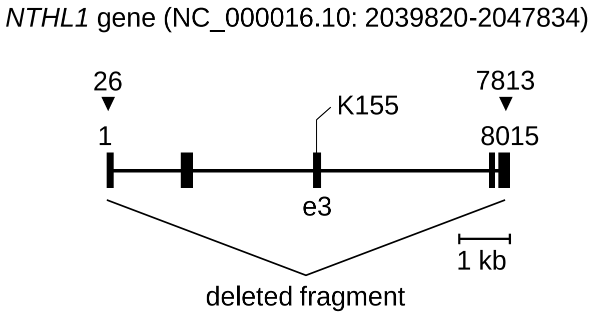

B

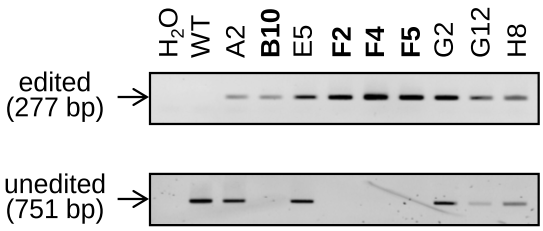

Supplementary Figure S4. NTHL1 knockout in HeLa cells by CRISPR-Cas9.

(A) Scheme of the *NTHL1* gene targeting using a pair of sgRNAs homologous to exons 1 and 5 (arrowheads). The generated deletion includes almost the entire protein coding sequence, including the catalytic lysine (K155) codon within the exon 3. SgRNA sequences are reported in Supplementary Table S1.

(B) PCR demonstration of the *NTHL1* gene deletion in chosen clones, as compared to the parental HeLa cell line (WT). A primer pair enclosing both sgRNA sites was used to amplify the rearranged (edited) *NTHL1* fragment; another pair was used to reject the clones displaying the native (unedited) fragment (Supplementary Table S1). Clones marked by bold typeface show only the band specific to the rearranged locus. The absence of the NTHL1 protein in the indicated clones was further confirmed by Western blotting (Figure 6).

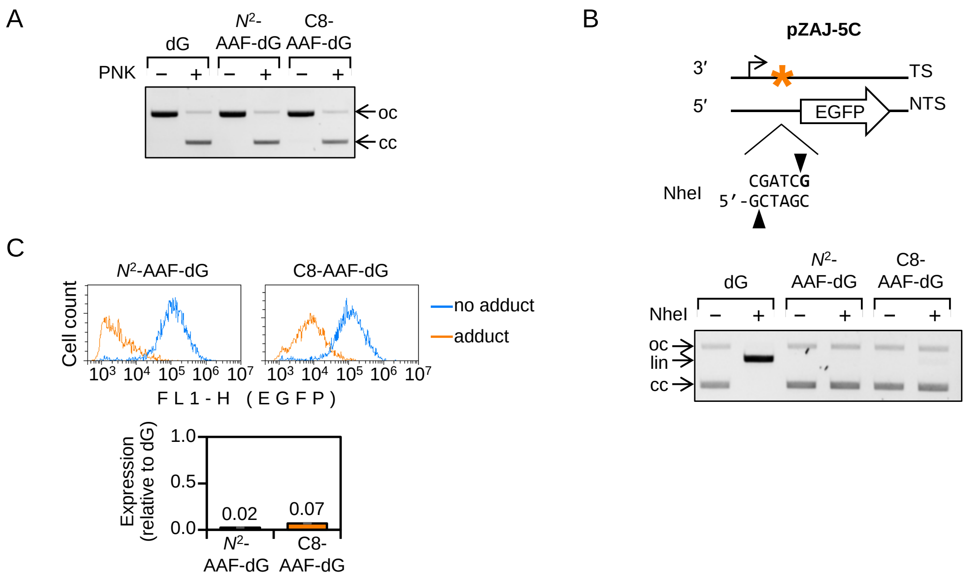

Supplementary Figure S5. Generation and characterisation of reporter constructs harbouring synthetic dG(N^2)-AAF and dG(C8)-AAF adducts.

(A) Incorporation of synthetic oligonucleotides containing the specified modifications into the 18-nucleotide gap in the transcribed strand of pZAJ-5C. Generation of the covalently closed plasmid DNA form ("cc") confirms the ligation rates of >89% for all constructs. Full suppression of the ligation in the absence of PNK indicates that the excised native DNA strand fragment was entirely substituted by the synthetic strands, as visualised by the open circular DNA form ("oc").

(B) Verification of the presence of adducts at the specified position by the inhibition of NheI cleavage. The indicated DNA constructs (100 ng) were incubated with 2 units NheI (NEB) to monitor conversion into the linearised form ("lin").

(C) Transcription blockage by adducts situated in the transcribed DNA strand, detected by the inhibition of the EGFP expression in transfected XP-A (GM04312) cells. Data of a representative experiment analysed with the CytoFLEX flow cytometer (fluorescence distribution plots) and the relative EGFP expression for transfections performed in duplicates (mean±SD).

A

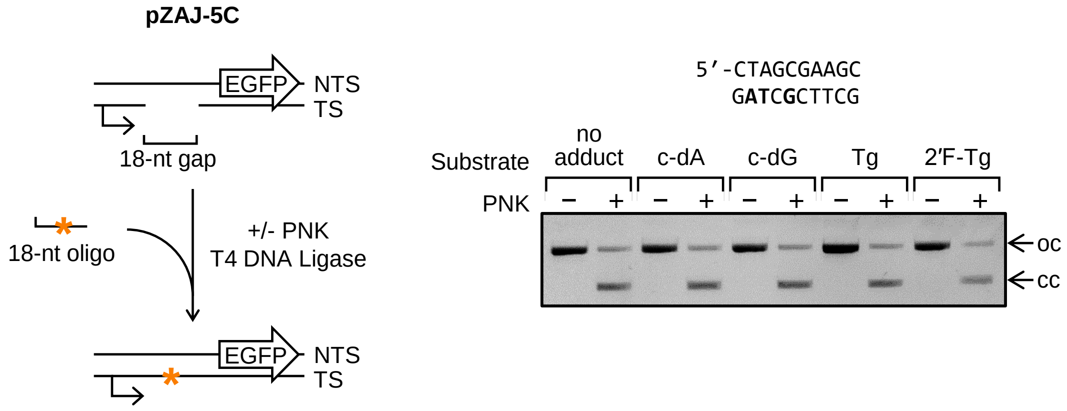

B

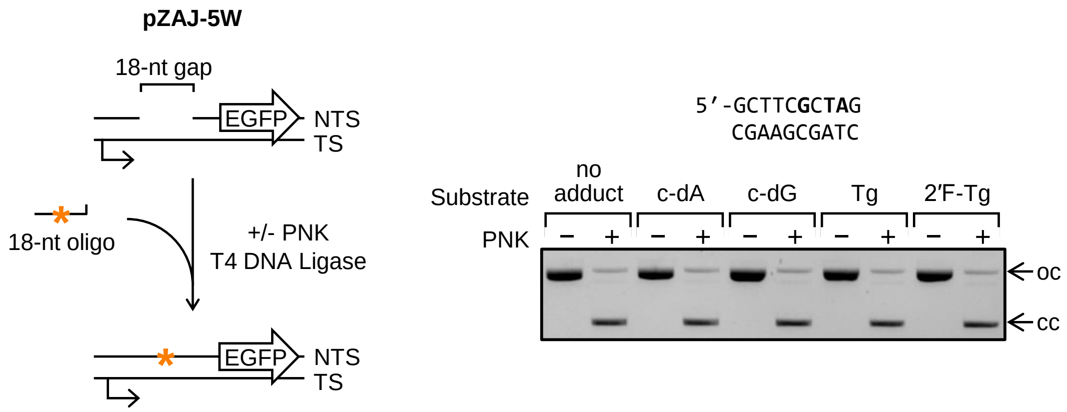

Supplementary Figure S6. Generation of reporter constructs harbouring synthetic cyclopurine adducts (c-dA or c-dG) or thymine glycol (Tg or 2′F-Tg) in the 5′-untranslated region of the EGFP gene.

(A) Highly efficient incorporation of synthetic oligonucleotides containing the specified modifications into the 18-nucleotide gap in the transcribed strand of pZAJ-5C. Covalently closed plasmid DNA form (cc) was generated in the presence of all modifications as efficiently as with the unmodified synthetic oligonucleotide (no adduct) when polynucleotide kinase (PNK) was added to the ligation reactions. In contrast, all DNA remained in the open circular form (oc) when PNK was omitted. Full suppression of the ligation reaction in the absence of PNK indicates that the excised native DNA strand fragment was entirely substituted by the synthetic strands.

(B) Incorporation of the same modifications as analysed in (A) into the non-transcribed DNA strand of the *EGFP* gene. The vector in this case was pZAJ-5W.

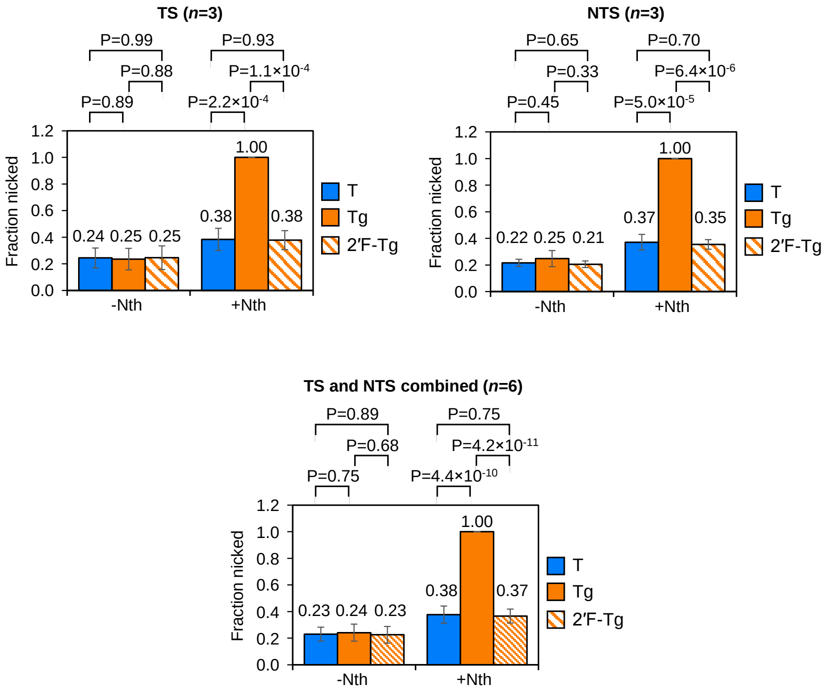

Supplementary Figure S7. Quantification of the endonuclease III (Nth) nicking activities towards the reporter constructs containing synthetic 2′-deoxyTg (Tg), its 2′α-fluorinated analog (2′F-Tg), or thymine (T) under the conditions of full cleavage of the Tg substrate. Six preparations of reporter constructs – three containing the modifications in the transcribed DNA strand (TS) and three in the non-transcribed strand (NTS) were analysed independently by incubation with 5 units Nth/100 ng DNA. Data shows the results of quantification of the open circular form of plasmid DNA after electrophoresis in the presence of ethidium bromide (mean±SD; P-values: Student’s two-tailed t-test).

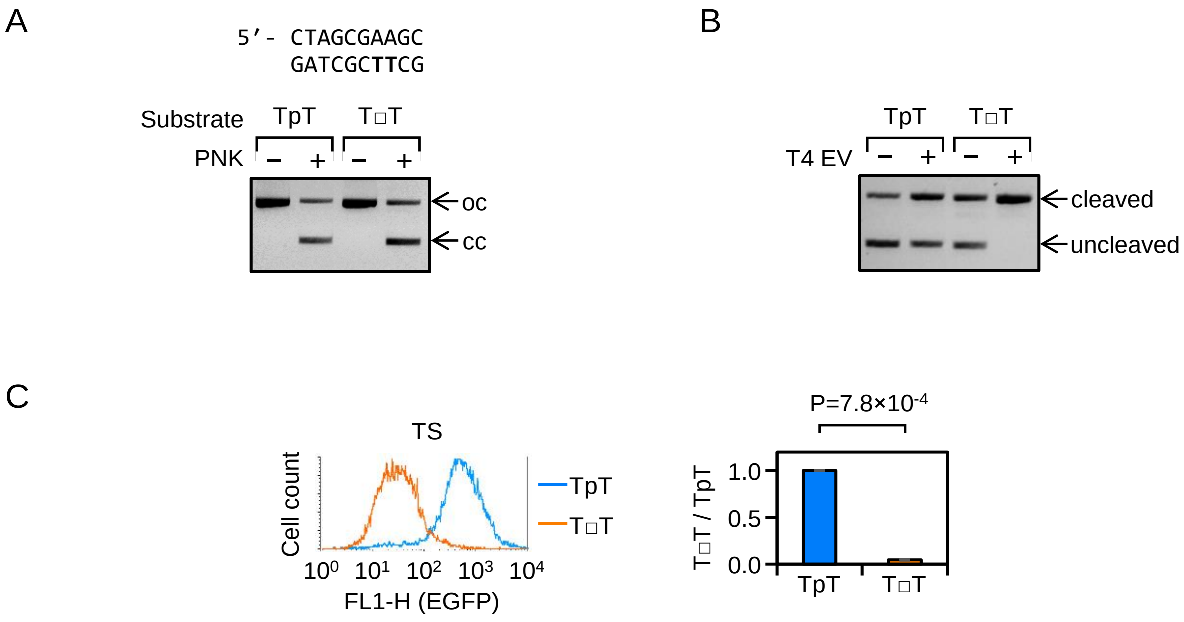

Supplementary Figure S8. Demonstration of transcription blockage by synthetic *cis*-*syn* cyclobutane TT dimer (T□T) in the transcribed DNA strand of the EGFP gene.

(A) High incorporation efficiency of the synthetic oligonucleotide containing single TT dimer (in the position indicated with bold typeface in the DNA sequence) and of the control modification-free oligonucleotide (TpT) into pZAJ-5C. Generation of covalently closed plasmid DNA (cc) was fully suppressed in the absence of PNK, compared to at least 85% in the presence of PNK, indicating that incorporation of both oligonucleotides was complete.

(B) Verification of the presence of the TT dimer in the vector DNA by its complete incision with T4 endonuclease V (T4 EV).

(C) EGFP expression analyses in XP-A (GM04312) cells transfected with constructs carrying no modification (TpT) or the TT dimer (T□T). Overlaid fluorescence distribution plots (on the left) and quantification of the EGFP expression (on the right). The values obtained in single independent experiments (*n=3*) were normalized to expression of the modification-free constructs (T□T/TpT) and the mean values calculated (± SD, P-value: Student’s two tailed *t*-test).

Supplementary Table S1. Positions and nucleotide sequences of sgRNA pairs used for CRISPR-Cas9 editing of the specified gene loci and the list of PCR primers used to screen single cell clones for the presence of specific deletions.

Edited gene locus (NCBI accession number)	sgRNA combinations		PCR primers	
	Position (cut site)	Sequence (5′→3′)	Position (5′)	Sequence (5′→3′)
CSA (NC_000005.10:c60936917-60866454)	41055<br>48437	AAACACGATTATGTGTGAGGTAAC<br>AAACACATAGAATACTTACGGTTGC	40890 (F)<br>41150 (R)	CTGTTACTAGTGATTGGAAGTAT<br>AGTAAGAGACCTCTATTATTAC
DDB2 (NC_000011.10:47214942-47239218)	17942<br>19986	AAACCCATTGAGGAGGCGTAAAAC<br>AAACGTCAACGCAGGTGTGATATC	17731 (F)<br>18021 (R)<br>20074 (R)	AGAGCGTCCTAGTGTCTAAG<br>CCCTGCTAGTTAAACTACTC<br>GGGTAAAGGTTTGACGTAGT
NTHL1 (NC_000016.10: 2039820-2047834)	26<br>7813	GCATGACCGCCTTGAGCGCGAGG<br>GCACGAGATCAATGGACTCTTGG	-98 (F)<br>7216 (R)<br>7947 (R)	GCTGGGAGTTGTAGTTCTGT<br>CGAGCTGTCGCTGTTATTGT<br>AAAGCCACTTCACAGACGGT

Extended Data: Synthesis Conditions Products Analysis

Conditions for the synthesis of the 2′F-Tg phosphoramidite

All reagents and solvents were purchased from commercial sources and used without further purification, unless otherwise stated. All reactions were carried out under argon atmosphere. Compounds were purified by flash chromatography on silica gel 40 – 60 µm, 60Å. NMR spectra were acquired on a Bruker Avance Neo Spectrometer (at 400 MHz for ^{1}H, 376 MHz for ^{19}F, 162 MHz for ^{31}P and 101 MHz for ^{13}C). Chemical shifts are given in ppm, using the residual solvent peaks as reference, for CDCl3 (δ 7.26 ppm for ^{1}H NMR and 77.16 ppm for ^{13}C NMR), DMSO-d6 (δ 2.50 ppm for ^{1}H NMR and 39.52 ppm for ^{13}C NMR). Coupling constants are reported in Hz. Atom assignments, where reported, are based on COSY, HSQC and HMBC spectra of the corresponding compounds. Mass Spectrometry experiments were carried out in a Thermo Finnigan Surveyor MSQ plus Mass Spectrometer, using the Electron Spray Ionization technique (ESI-MS). High Resolution Mass Spectrometry experiments were carried out in a Q-TOF Bruker MaXis Impact HR-Mass Spectrometer. Selected ^{1}H, ^{13}C, COSY, HSQC and HMBC NMR spectra of compounds **2-9** are reported (Extended data 1–33).

2,2′-Anhydrothymidine (**2**)

To a solution of **1** (15.5 g, 50 mmol, 1 eq) in anhydrous DMF (50 mL) were added diphenyl carbonate (12.9 g, 60 mmol, 1.2 eq) and $NaHCO_3$ (250 mg, 2.5 mmol, 0.05 eq) and the mixture was heated to reflux for 3 hours. The reaction mixture was cooled to room temperature and water was added. The water layer was washed 4 times with diethyl ether and then was evaporated under reduced pressure. The residue was recrystallized by MeOH and dried under reduced pressure to give 9.80 g (82%) of **2** as white powder.

^{1}H NMR (400 MHz, DMSO-*d*$_6$) δ 7.73 (d, *J* = 1.6 Hz, 1H), 6.29 (d, *J* = 5.7 Hz, 1H), 5.89 (s, 1H), 5.18 (d, *J* = 5.7 Hz, 1H), 4.95 (m, 1H), 4.38 (d, *J* = 1.8 Hz, 1H), 4.06 (td, *J* = 5.7, 1.7 Hz, 1H), 3.26 (dd, *J* = 11.6, 5.1 Hz, 1H), 3.17 (dd, *J* = 11.6, 6.1 Hz, 1H), 1.79 (s, 3H) ppm.

ESI-MS (+): 241.08 $[M+H]^+$, 263.01 $[M+Na]^+$, 481.31 $[2M+H]^+$. 503.17 $[2M+Na]^+$

Benzylation of 2′-anhydrothymidine

To a solution of **2** (2.30 g, 8.2 mmol, 1 eq) in anhydrous DMF (40 mL) was added BnBr (2.5 mL, 21.3 mmol, 2.6 eq) and the solution was cooled to 0 ˚C for 30 min. Then NaH (1.30 g, 28 mmol, 3 eq) was added at the reaction mixture in portions and the mixture was stirred at room temperature for 16 h. Then was quenched with saturated $NaHCO_3$, extracted with ethyl acetate, washed twice with saturated $NaHCO_3$, water and Brine. The organic layer was dried over Na_2SO_4 and evaporated under reduced pressure. The residue was purified by column chromatography using 10/90 EtOAc/PE to 20% MeOH/ EtOAc.

Three different products were isolated and fully characterized as following:

2′,3′,5′-tribenzyl-*O*-2′-arabinothymidine (3a) 1.214 mg, 30% as yellow oil: **^{1}H NMR (200 MHz, Chloroform-*d*)** δ 7.48 – 7.41 (m, 1H), 7.42 – 6.99 (m, 15H), 6.32 (dd, *J* = 5.2, 1.4 Hz, 1H), 4.75 – 4.43 (m, 5H), 4.41 (d, *J* = 3.1 Hz, 2H), 4.30 – 4.20 (m, 1H), 4.11 (ddd, *J* = 11.6, 6.8, 3.9 Hz, 2H), 3.68 (q, *J* = 4.0 Hz, 2H), 1.67 (q, *J* = 1.2 Hz, 3H) ppm;

3′,5′-dibenzyl-*O*-2,2′-anhydrothymidine (3b) 750 mg, 22% as a yellow powder:**^{1}H NMR (200 MHz, Chloroform-*d*)** δ 8.42 – 7.21 (m, 7H), 7.17 (dtd, *J* = 5.7, 4.9, 2.8 Hz, 3H), 6.17 (d, *J* = 6.0 Hz, 1H), 5.24 (dt, *J* = 6.0, 0.6 Hz, 1H), 4.62 (d, *J* = 11.7 Hz, 1H), 4.54 (d, *J* = 11.7 Hz, 1H), 4.41 (d, *J* = 2.3 Hz, 1H), 4.36 (d, *J* = 8.7 Hz, 1H), 4.29 (d, *J* = 0.7 Hz, 1H), 4.25 (d, *J* = 9.9 Hz, 1H), 3.27 (d, *J* = 4.2 Hz, 1H), 3.26 (d, *J* = 4.1 Hz, 1H), 1.96 (d, *J* = 1.3 Hz, 3H) ppm; **^{13}C NMR (50 MHz, Chloroform-*d*)** δ 172.39, 159.44, 136.61, 136.08, 130.51, 128.27, 128.06, 127.92, 127.65, 118.00, 90.49, 85.93, 85.25, 83.33, 73.08, 71.79, 68.51, 13.70 ppm; **ESI-MS (+):** 421.29 $[M+H]^+$, 442.82 $[M+Na]^+$, 841.47 $[2M+H]^+$.
3′,5′-dibenzyl-*O*-2′-arabinothymidine (3c) 1600 mg, 45% as colorless oil: **^{1}H NMR (400 MHz, Chloroform-*d*)** δ 10.01 (s, 1H), 7.51 (d, *J* = 1.6 Hz, 1H), 7.34 (s, 10H), 6.17 (d, *J* = 3.7 Hz, 1H), 4.77 (d, *J* = 11.9 Hz, 1H), 4.66 – 4.58 (m, 4H), 4.57 (d, *J* = 3.7 Hz, 1H), 4.21 (q, *J* = 3.8 Hz, 1H), 4.04 (dd, *J* = 3.7, 2.0 Hz, 1H), 3.81 (dd, *J* = 10.5, 3.4 Hz, 1H), 3.71 (dd, *J* = 10.5, 4.2 Hz, 1H), 1.70 (d, *J* = 1.2 Hz, 3H) ppm; **^{13}C NMR (101 MHz, Chloroform-*d*)** δ 165.00, 150.77, 138.39, 137.57, 137.25, 130.88 – 123.85 (m), 116.44, 108.65, 86.75, 83.85, 81.76, 73.76 (2x), 71.98, 69.67, 12.38 ppm; **HRMS**: (ESI) theoretical $[M+Na]^+$: 461.1683, calculated $[M+Na]^+$: 461.1678

3′,5′-*O*-dibenzyl-2′α-fluorothymidine (4)

To a solution of **3c** (3.00 g, 6.8 mmol, 1 eq) in dry DCM (57 mL) was added dry pyridine (6.8 mL) and the solution was cooled to 0 ˚C. Then DAST (2.7 mL, 20.4 mmol, 3 eq) was added, the reaction mixture was allowed to reach room temperature and then heated to reflux for 3 hours. The reaction was quenched with saturated $NaHCO_3$, extracted with DCM, washed with $NaHCO_3$, water and brine. The organic layer was dried over Na_2SO_4, evaporated under reduced pressure, and purified by column chromatography 40/60 EtOAc/PE to 60/40 EtOAc/PE to give 2.40 g (80%) **4** as a white foam.
The product was characterized as following: **^{1}H NMR (400 MHz, $CDCl_3$)** δ 9.48 (s, 1H), 7.45 (d, *J* = 1.7 Hz, 1H), 7.31 – 7.20 (m, 8H), 7.19-7.12(m, 2H), 5.97 (dd, *J* = 16.6, 1.9 Hz, 1H), 5.00 (ddd, *J* = 52.4, 4.1, 1.9 Hz, 1H), 4.67 (d, *J* = 11.7 Hz, 1H), 4.47 (d, *J* = 9.0 Hz, 2H), 4.43 (t, *J* = 9.6 Hz, 1H), 4.23 – 4.19 (m, 1H), 4.14 (ddd, *J* = 19.0, 7.7, 4.0 Hz, 1H), 3.84 (dd, *J* = 11.1, 2.1 Hz, 1H), 3.58 (dd, *J* = 11.1, 2.5 Hz, 1H), 1.53 – 1.26 (m, 3H) ppm; **^{13}C NMR (101 MHz, Chloroform-*d*)** δ 164.06, 150.29, 137.31, 137.13, 135.78, 128.62(x2), 128.58(x2), 128.22, 128.13, 127.90(x2), 127.79(x2), 110.90, 91.63 (d, *J* = 191.6 Hz), 88.69 (d, *J* = 34.2 Hz), 80.95 (d, *J* = 1.3 Hz), 74.65 (d, *J* = 15.8 Hz), 73.64, 72.80, 67.95, 11.99 ppm; **^{19}F NMR (376 MHz, Chloroform-*d*)** δ -202.7 ppm (s); **ESI-MS (+):** 441.13 $[M+H]^+$, 458.19 $[M+Na]^+$, 881.39 $[2M+H]^+$.

3′,5′-*O*-dibenzyl-2′-fluorothymidine glycol (5)

To a solution of OsO_4 (700 mg, 2.6 mmol, 1 eq) in dry pyridine (3 mL) was added a solution of **4** (1.150 mg, 2.6 mmol, 1 eq) in dry pyridine (3.5 mL). The reaction mixture was stirred in room temperature for 2.5 h and then aqueous solution of $NaHSO_3$ (1.8 g $NaHSO_3$ in 1 mL H_2O) and pyridine (7 mL) were added. The brown mixture was stirred for 12 hours at room temperature and then was extracted with DCM, washed with water, brine and the organic layer was dried over Na_2SO_4 and evaporated to give 1.20 g (98%) **5** as a mixture of diastereomers.
The products were characterized as following: **^{1}H NMR (400 MHz, Chloroform-d)** δ 8.26 (s, 1H), 7.34 (d, *J* = 4.9 Hz, 8H), 7.29 – 7.23 (m, 2H), 5.17 (dd, *J* = 52.9, 4.3 Hz, 1H), 5.15 (d, *J* = 4.3 Hz, 1H), 4.75

(dd, *J* = 11.8, 4.5 Hz, 1H), 4.59 – 4.47 (m, 3H), 4.45 (d, *J* = 11.5 Hz, 1H), 4.26 (ddd, *J* = 20.4, 8.5, 4.3 Hz, 1H), 4.22 – 4.10 (m, 1H), 3.87 (d, *J* = 11.1 Hz, 1H), 3.61 (dd, *J* = 11.4, 2.9 Hz, 1H), 1.37 (s, 3H) ppm; **^{13}C NMR (101 MHz, Chloroform-*d*)** δ 173.60, 150.40, 137.41, 137.29, 128.76(x2), 128.62(x2),128.29 128.28, 128.22(x2), 128.14(x2), 91.71 (d, *J* = 188.9 Hz), 88.28 (d, *J* = 34.2 Hz), 80.06, 78.23, 74.63 (d, *J* = 15.8 Hz), 73.77, 72.76, 71.91, 68.11, 22.90 ppm: **^{19}F NMR (376 MHz, Chloroform-*d*)** δ -198.2 (2 %), -201.3 (81 %), -201.4 (12 %), 201.7 ppm (5 %).

3′,5′-*O*-dibenzyl-5,6-dihydro-5,6-di[(tert-butyl)dimethylsilyloxy]-2′-fluorothymidine (6)

To a solution of **5** (1.200 mg, 2.54 mmol, 1 eq) in dry DMF (26 mL) were added imidazole (1.700 mg, 26.0 mmol, 10 eq) and TBDMS-Cl (1.900 mg, 12.6 mmol, 5.2 eq) and the reaction mixture was stirred at room temperature for 48h. The TBDMS-Cl was quenched with $NaHCO_3$, extracted with ethyl acetate, washed with aqueous $NaHCO_3$, water and brine. The organic layer was collected, dried over Na_2SO_4 and evaporated. The residue was purified with column chromatography to afford **6** (1.100 mg, 76%) as white solid. The product was characterized as following: **^{1}H NMR (400 MHz, Chloroform-*d*)** δ 7.42 (s, 1H), 7.39 – 7.24 (m, 10H), 5.46 – 5.32 (m, 2H), 4.83 – 4.71 (m, 2H), 4.65 – 4.56 (m, 2H), 4.50 (d, *J* = 12.0 Hz, 1H), 4.31 – 4.18 (m, 1H), 4.17 – 4.13 (m, 1H), 3.72 (dd, *J* = 10.8, 3.1 Hz, 1H), 3.64 (dd, *J* = 10.9, 4.9 Hz, 1H), 1.49 (s, 3H), 0.92 (s, 9H), 0.87 (s, 9H), 0.30 (s, 3H), 0.25 (s, 3H), 0.19 (s, 3H), 0.15 (s, 3H) ppm; **^{13}C NMR (101 MHz, Chloroform-*d*)** δ 172.70, 151.35, 138.15, 137.66, 128.56(x2), 128.49(x2), 128.25, 128.06, 128.03(x2), 127.77(x2),), 92.39 (d, *J* = 34.3 Hz), 90.79 (d, *J* = 188.9 Hz), 87.90, 80.66, 79.88, 76.97, 76.43 (d, *J* = 14.8 Hz), 73.60, 72.93, 70.09, 26.47(x3), 25.82(x3), 24.02, 18.71, 18.17, -1.97, -2.34, -3.80, -4.59 ppm; **^{19}F NMR (376 MHz, Chloroform-*d*)** δ -197.8 (14 %), -201.0 ppm (86 %).

5,6-dihydro-5,6-di[(tert-butyl)dimethylsilyloxy]-2′-fluorothymidine (7)

To a solution of the TBDMS-protected nucleoside **6** (1000 mg, 1.40 mmol, 1 eq) in ethanol was added 10% Pd/C (170 mg, 0.02 eq). The mixture was stirred for 4h under hydrogen atmosphere at room temperature. Then it was filtered from celite plug under reduced pressure, the residue was evaporated to afford **7** (700 mg, 96%) as a white solid. The product was characterized as following: **^{1}H NMR (400 MHz, DMSO-d_6)** δ 10.55 (s, 1H), 5.58 (dd, *J* = 19.3, 4.5 Hz, 1H), 5.48 (d, *J* = 5.5 Hz, 1H), 5.12 (s, 1H), 5.08 (dt, *J* = 54.1, 4.9 Hz, 1H), 4.76 (t, *J* = 4.8 Hz, 1H), 4.18 (dd, *J* = 11.4, 5.4 Hz, 1H), 3.76 (d, *J* = 4.2 Hz, 1H), 3.57 (dt, *J* = 6.8, 3.3 Hz, 1H), 3.51 (q, *J* = 5.8 Hz, 1H), 1.36 (s, 3H), 0.87 (s, 9H), 0.80 (s, 9H), 0.21 (s, 3H), 0.15 (s, 3H), 0.12 (s, 3H), 0.08 (s, 3H) ppm: **^{13}C NMR (101 MHz, DMSO-d_6)** δ 173.63, 151.90, 91.70 (d, *J* = 186.1 Hz), 88.74 (d, *J* = 33.4 Hz), 84.68, 84.01, 76.63, 69.44 (d, *J* = 15.6 Hz), 61.85, 26.72(x3), 26.10(x3), 23.97, 18.84, 18.26, -1.51, -1.74, -3.45, -4.14 ppm; **^{19}F NMR (376 MHz, DMSO-d_6)** δ -200.4 (9 %), -203.22 ppm (91 %); **HRMS:** (ESI) calculated for $C_{22}H_{43}FN_2NaO_7Si_2^+$ $[M+Na]^+$: 545.2485, found: 545.2478

5′-*O*-(4,4′-Dimethoxytrityl)-5,6-dihydro-5,6-di[(tert-butyl)dimethylsilyloxy]-2′-fluorothymidine (8)

To a solution of **7** (700 mg, 1.35 mmol, 1 eq) in dry pyridine (4 mL) was added DMAP (60 mg, 0.05 eq) and a solution of DMT-Cl (472 mg, 1.89 mmol, 1.4 eq) in dry pyridine (2 mL). The mixture was stirred for 16h under argon atmosphere at room temperature. Then it was quenched with methanol,

evaporated under reduced pressure, diluted in ethyl acetate, washed with water and Brine. The organic layer was dried over Na_2SO_4, filtered, and evaporated under reduced pressure. The residue was purified with column chromatography 10-40% EtOAc/PE (in the presence of 1% Et_3N) to give **8** (860 mg, 75%) as a white solid.

The products were characterized as following: Major Isomer (5*R*,6*S*) (3): **^{1}H NMR (400 MHz, Chloroform-*d*)** δ 7.51 (d, *J* = 7.5 Hz, 2H), 7.41 – 7.38 (m, 4H), 7.34 – 7.29 (m, 2H), 7.25 – 7.21 (m, 1H), 6.86 (d, *J* = 8.4 Hz, 4H), 5.39 – 5.18 (m, 1H), 5.27 – 5.14 (m, 1H), 4.72 (s, 1H), 4.41 (dt, *J* = 15.9, 6.4 Hz, 1H), 3.94 (td, *J* = 6.8, 3.7 Hz, 1H), 3.83 (s, 6H), 3.46 (dd, *J* = 10.3, 3.6 Hz, 1H), 3.40 (dd, *J* = 10.3, 5.9 Hz, 1H), 1.52 (s, 3H), 0.94 (s, 9H), 0.92 – 0.88 (m, 9H), 0.33 (s, 3H), 0.29 (s, 3H), 0.26 (s, 3H), 0.22 (s, 3H) ppm; Major Isomer (5*R*,6*S*): **^{13}C NMR (101 MHz, Chloroform-*d*)** δ 172.52, 158.57(x2), 150.73, 144.90, 136.15(x2), 130.21(x4), 129.15, 128.34, 127.89, 126.86, 125.42, 113.23(x4), 91.70 (d, *J* = 188.7 Hz), 88.74 (d, *J* = 40.3 Hz),, 89.05, 86.40, 81.61, 76.93, 70.37 (d, *J* = 16.3 Hz), 63.82, 55.29(x2), 26.48(x3), 25.85(x3), 24.06, 18.70, 18.15, -1.96, -2.36, -3.87, -4.45 ppm; **^{19}F NMR (376 MHz, $CDCl_3$)** δ Minor Isomer (5*S*,6*R*): -194.0 (11 %), Major Isomer (5*R*,6*S*): -198.9. ppm (89 %).

5′-*O*-(4,4′-Dimethoxytrityl)-5,6-dihydro-5,6-di[(tert-butyl)dimethylsilyloxy]-2′-fluorothymidine-3′-[(2-cyanoethyl)-N,N-diisopropyl]phosphoramidite (9)

To a degassed solution of **8** (500 mg, 0.60 mmol, 1 eq) in dry DCM (16 mL) was added DIPEA (300 µl, 1.8 mmol, 3 eq) and dropwise addition of (2-cyanoethyl)-*N*,*N*′-diisopropylchlorophosphoramidite (400 µl, 1.8 mmol, 3 eq). The reaction mixture was stirred for 3 hours under argon and then it was quenched with methanol. After 10 minutes the solvents were removed by rotary evaporator and the residue was purified with column chromatography in 30-50% EtOAc/PE (in the presence of pyridine 2%) and under argon pressure to afford the 400 mg (67%) of the phosphoramidite derivative as a white foam. Silica gel as well as all solvents used were flushed with argon and the fractions were collected in 20 mL vials and were continuously flushed with argon.

The products were characterized as following: **^{13}C NMR (101 MHz, DMSO-*d*$_6$)** δ 172.52, 158.53(x2), 150.60, 144.93, 136.16(x2), 130.26(x4), 128.39(x2), 127.82(x2), 126.79, 113.15(x4), 93.78 (d, *J* = 299 Hz), 89.40 (d, *J* = 20.6 Hz), 89.19, 86.27, 81.23, 76.93, 70.46, 63.78, 63,22, 55.28(x2), 43.42(x2), 26.45(x3), 25.84(x3), 24.71(x4), 24.04, 20.44, 18.68, 18.11, -2.01, -2.41, -3.98, -4.44 ppm; **^{19}F NMR (376 MHz, $CDCl_3$)** δ Minor Isomer (5*S*,6*R*, 11 %): -190.7 (d, *J* = 6.5 Hz), -1901.1 (d, *J* = 11.3 Hz), Major Isomer (5*R*,6*S*, 89 %): -196.1 (d, *J* = 14.7 Hz), -196.5 ppm (d, *J* = 9.4 Hz); **^{31}P NMR (162 MHz, Chloroform-*d*)** δ Minor Isomer (5*S*,6*R*, 11 %): 150.9 (d, *J* = 6.0 Hz), 149.9 (d, *J* = 11.6 Hz). Major Isomer (5*R*,6*S*, 89 %): 150.6 (d, *J* = 9.8 Hz), 150.1 ppm (d, *J* = 14.1 Hz); **HRMS:** (ESI) calculated for $C_{52}H_{78}FN_4NaO_{10}PSi_2^+$ $[M+Na]^+$: 1047.4870, found: 1047.4861

NMR spectra of intermediates and of the final protected 2′F-Tg phosphoramidite (Extended Data 1−33)

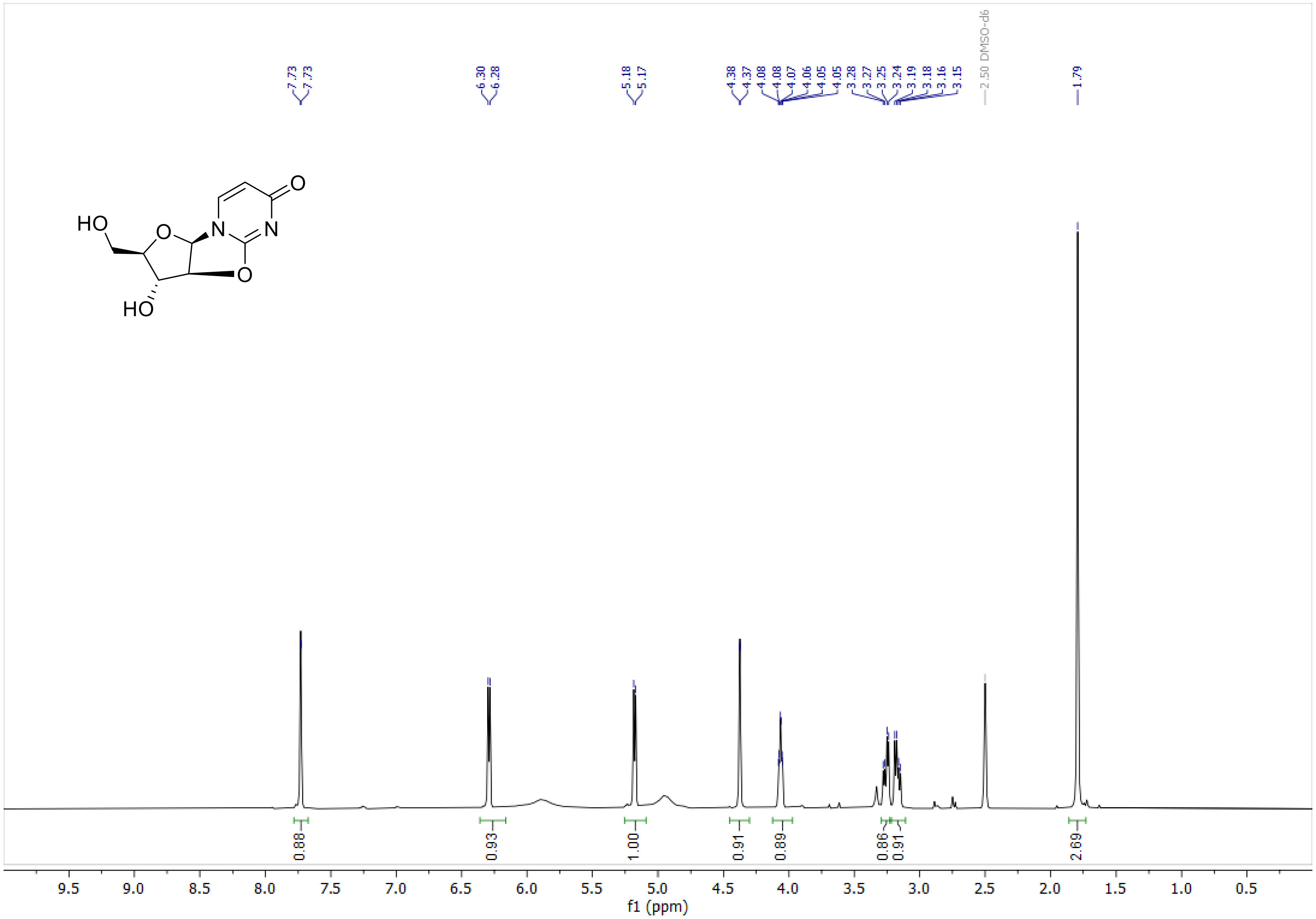

Extended Data 1. ^{1}H NMR spectrum of **2** (400 MHz, dmso-d_6)

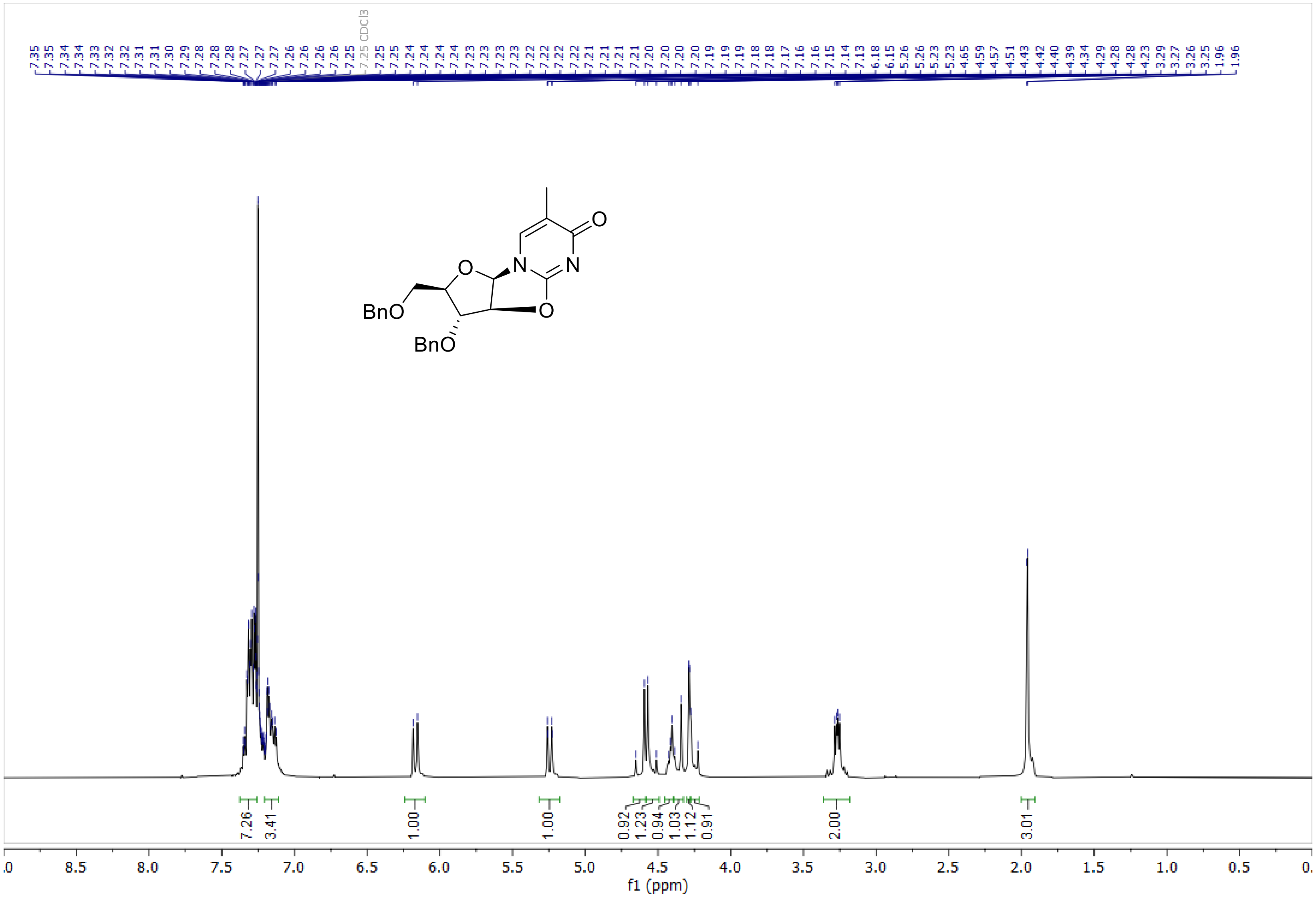

Extended Data 2. ^{1}H NMR spectrum of **3b** (400 MHz, $CDCl_3$)

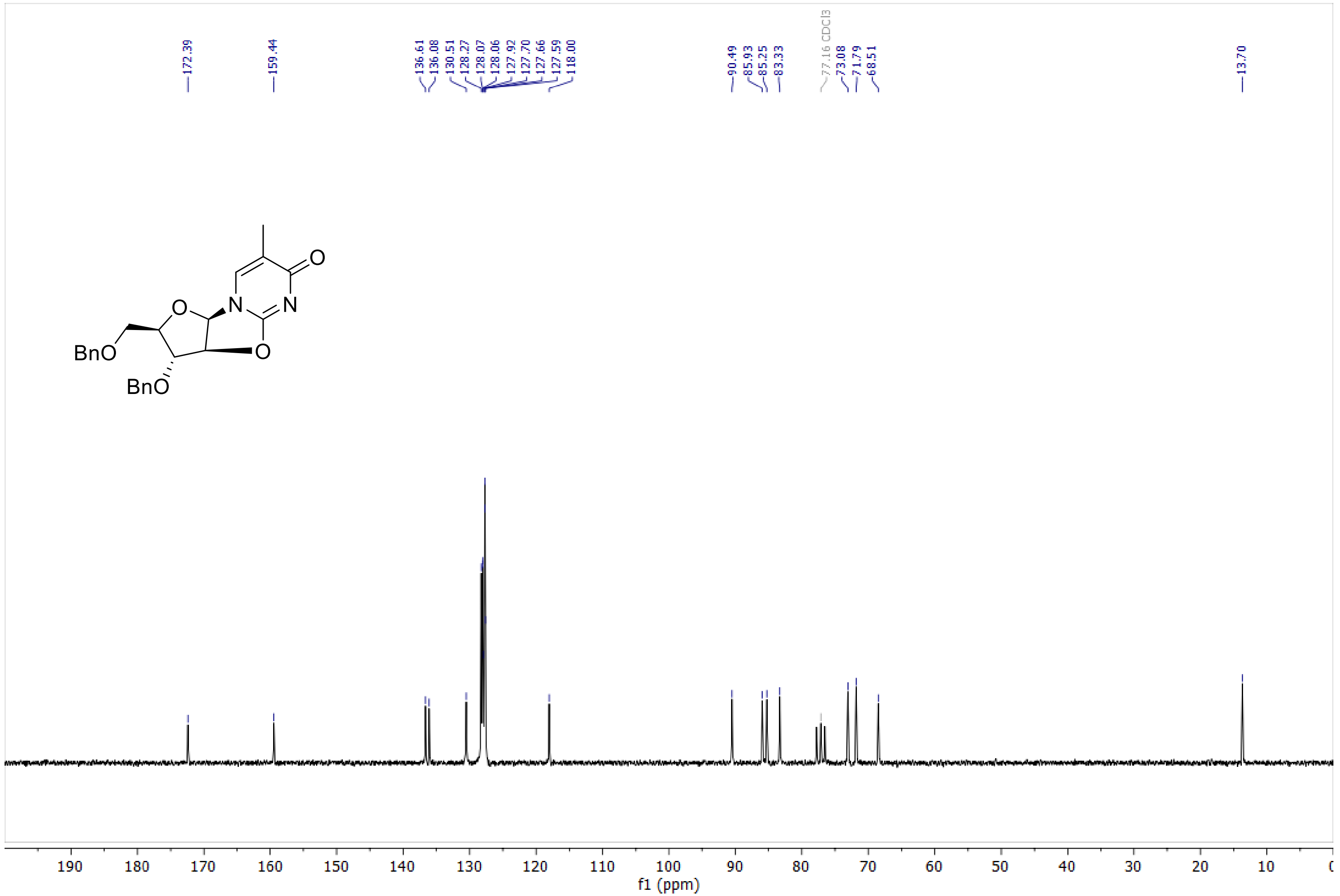

Extended Data 3. ^{13}C NMR spectrum of **3b** (101 MHz, $CDCl_3$)

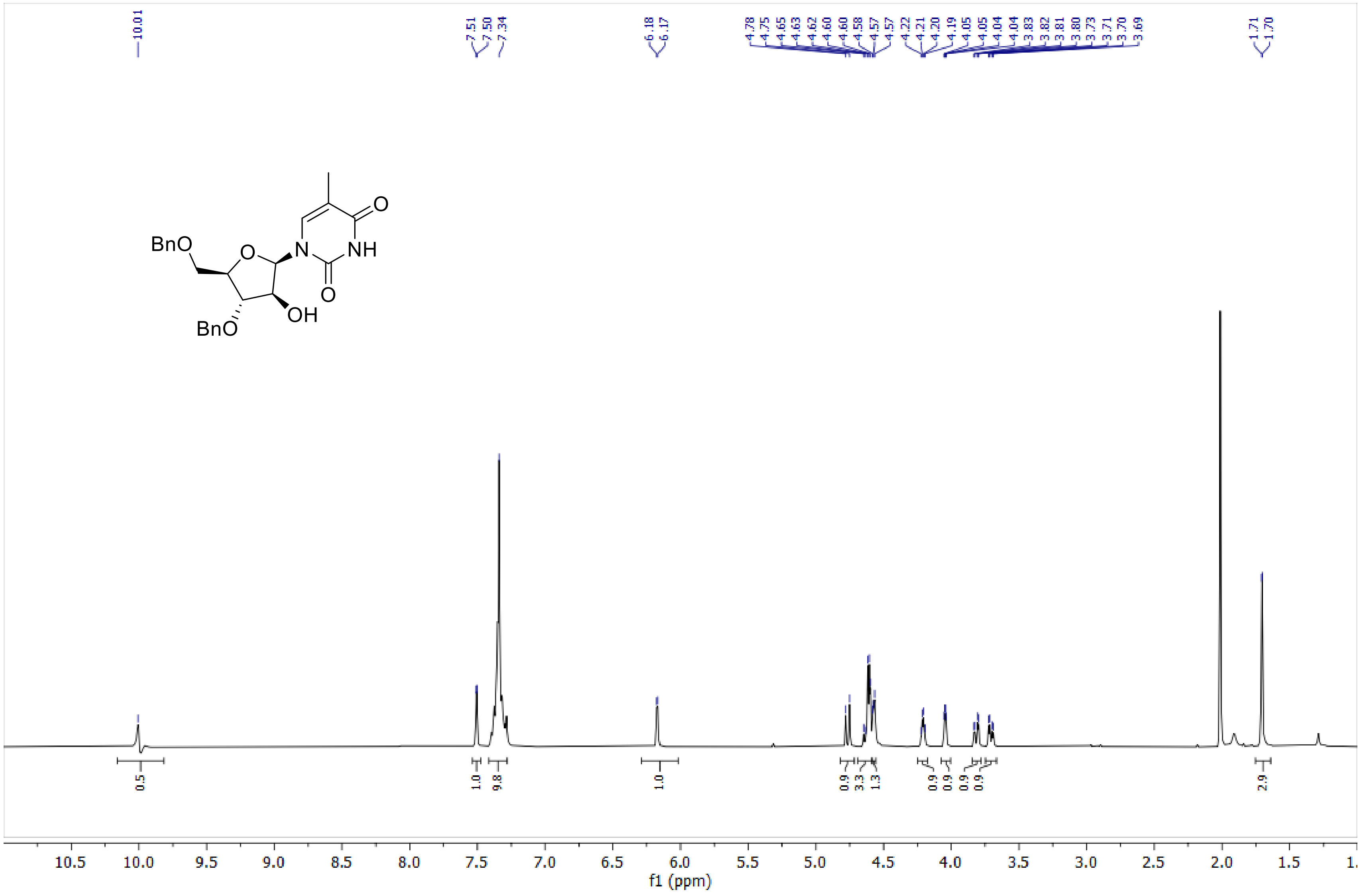

Extended Data 4. ^{1}H NMR spectrum of **3c** (400 MHz, $CDCl_3$)

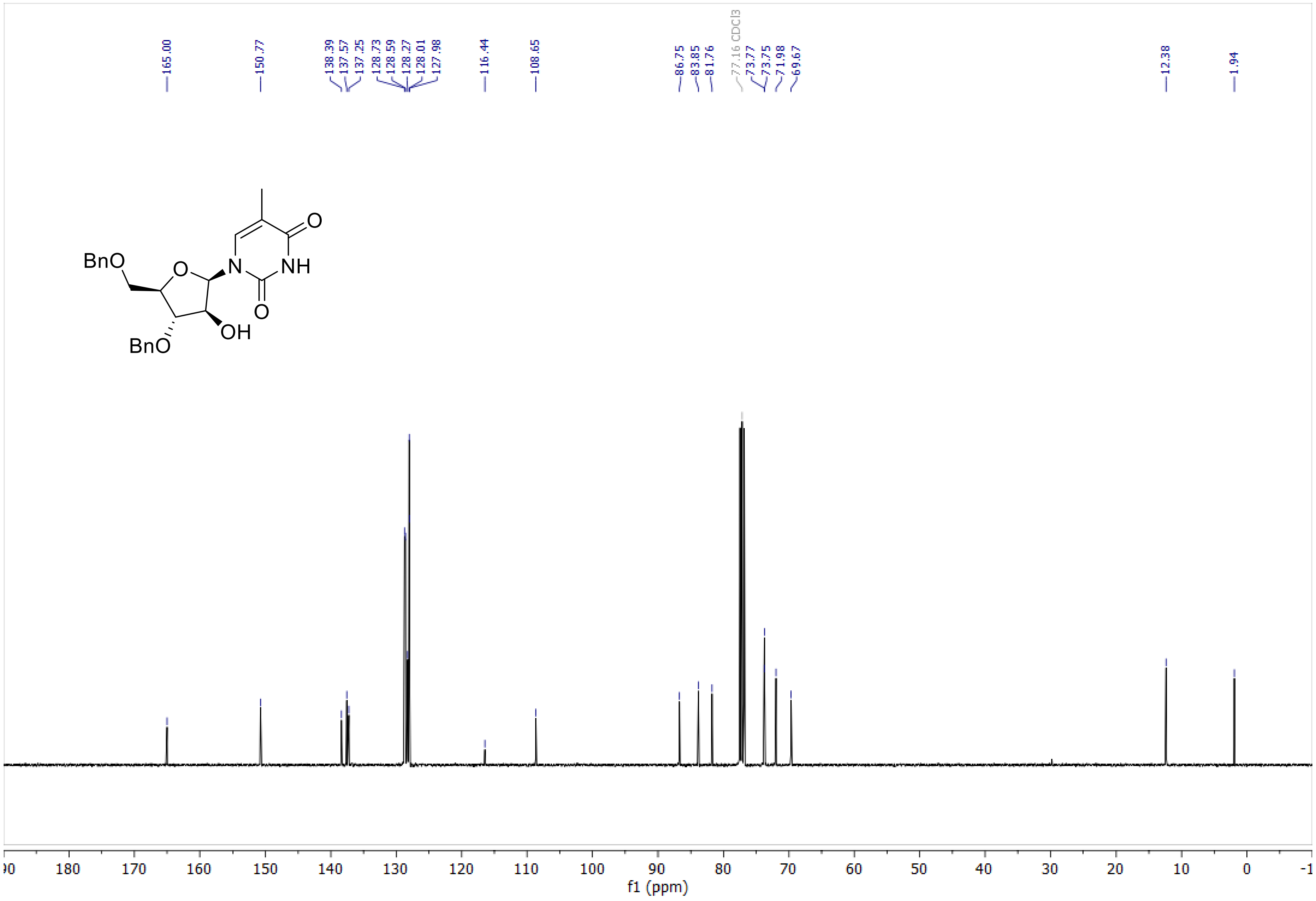

Extended Data 5. ^{13}C NMR spectrum of **3c** (101 MHz, $CDCl_3$)

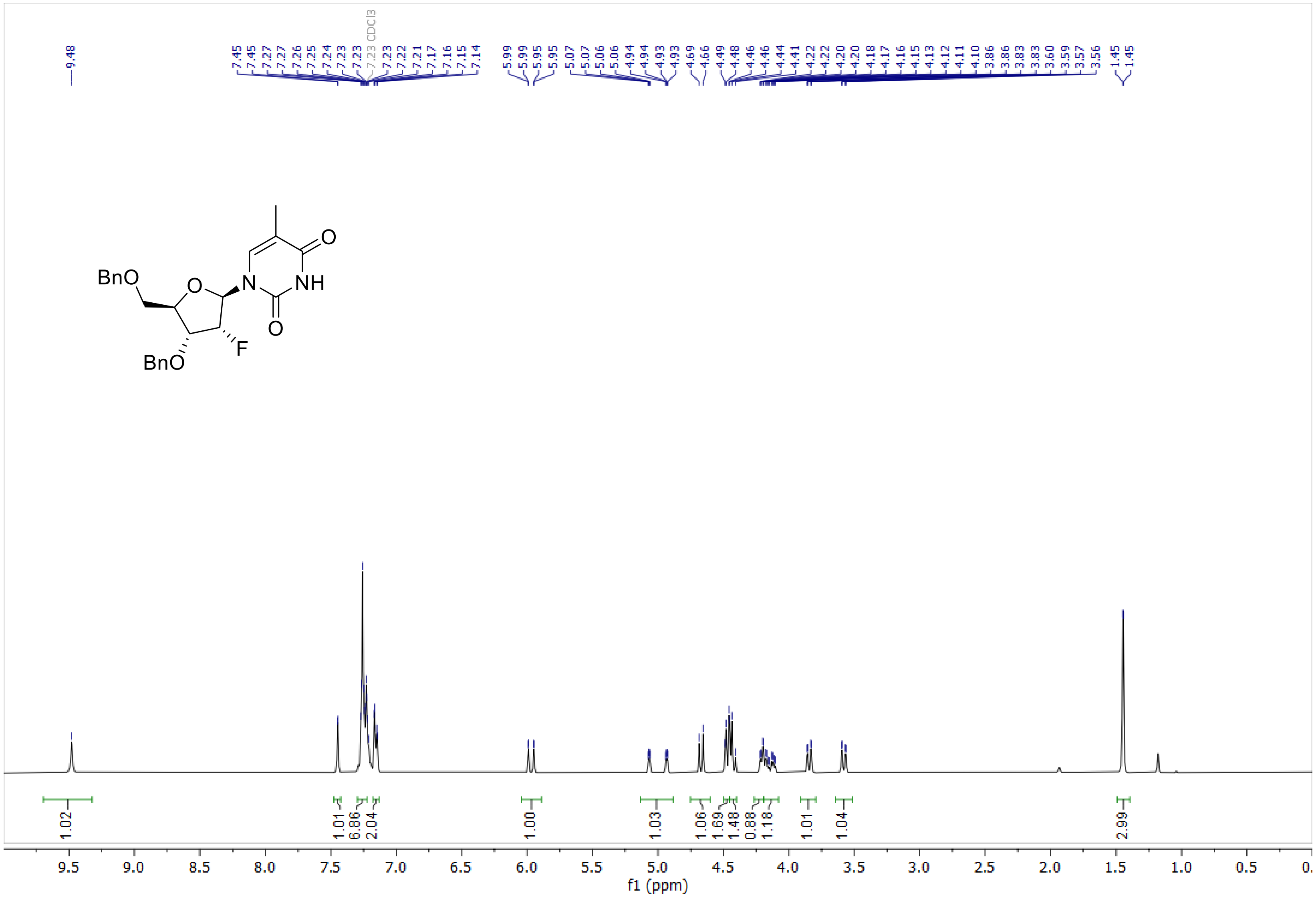

Extended Data 6. ^{1}H NMR spectrum of **4** (400 MHz, $CDCl_3$)

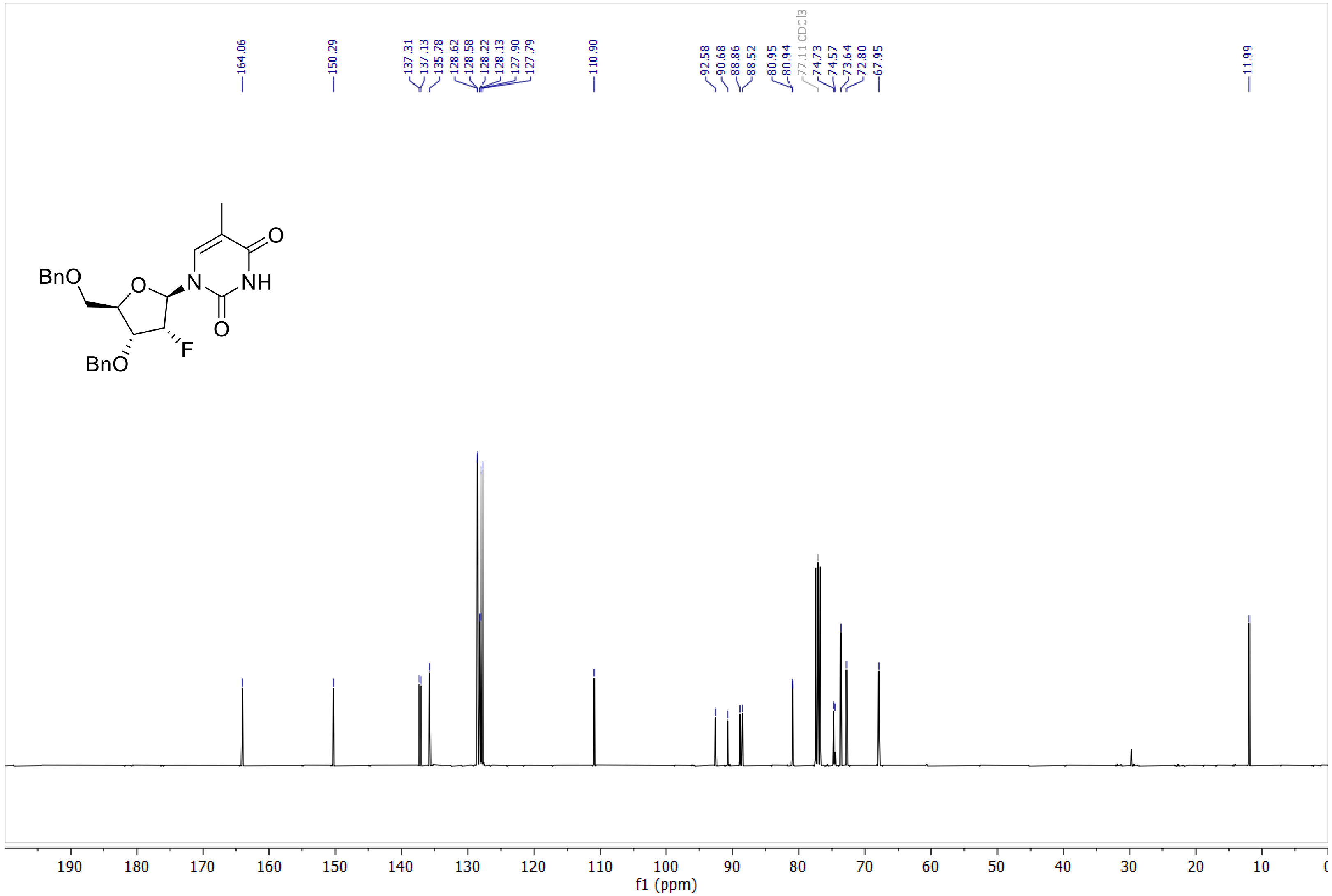

Extended Data 7. ^{13}C NMR spectrum of **4** (101 MHz, $CDCl_3$)

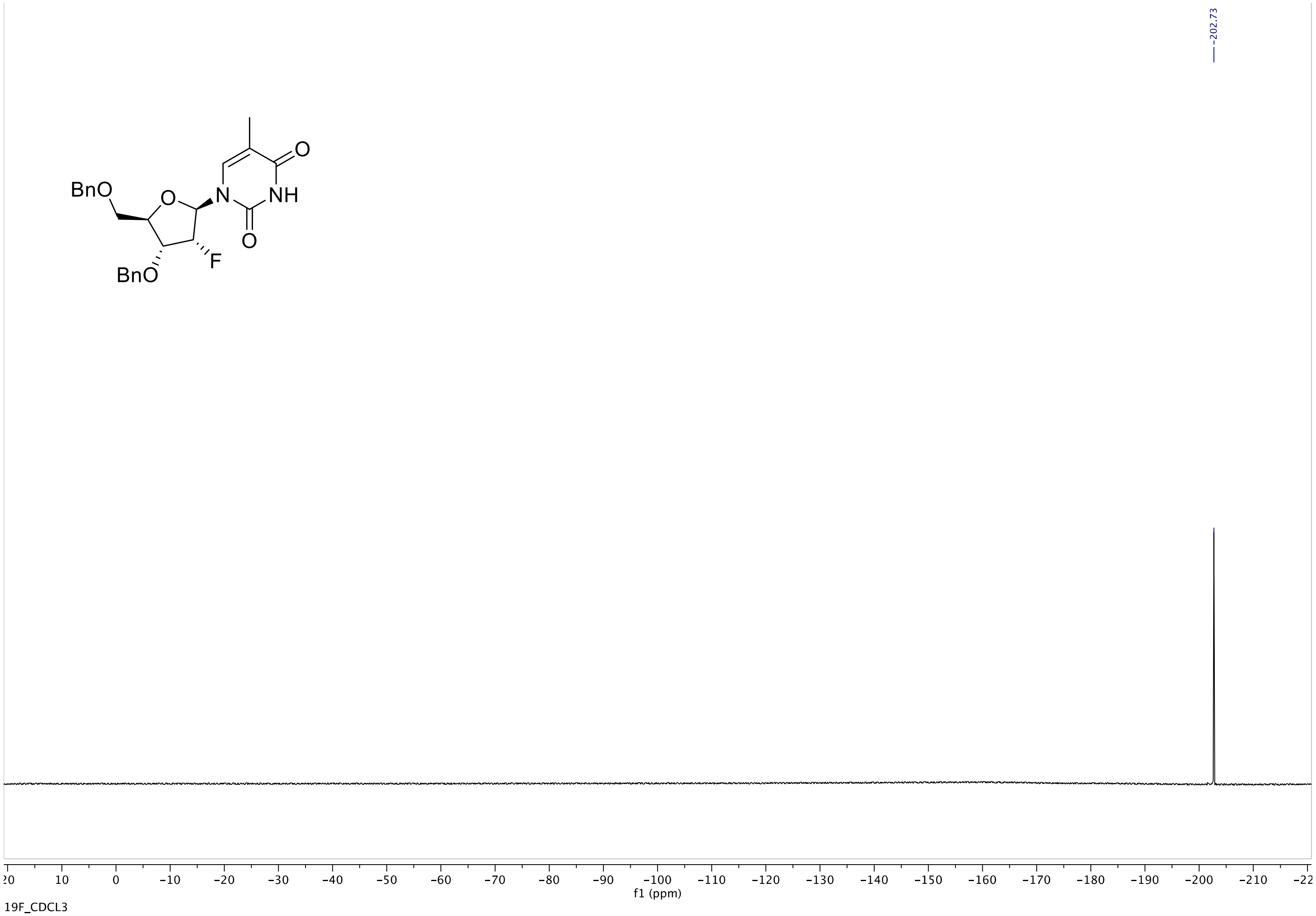

Extended Data 8. ^{19}F NMR spectrum of **4** (376 MHz, $CDCl_3$)

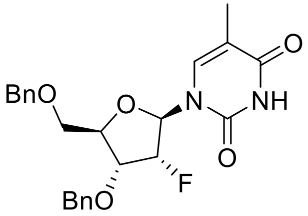

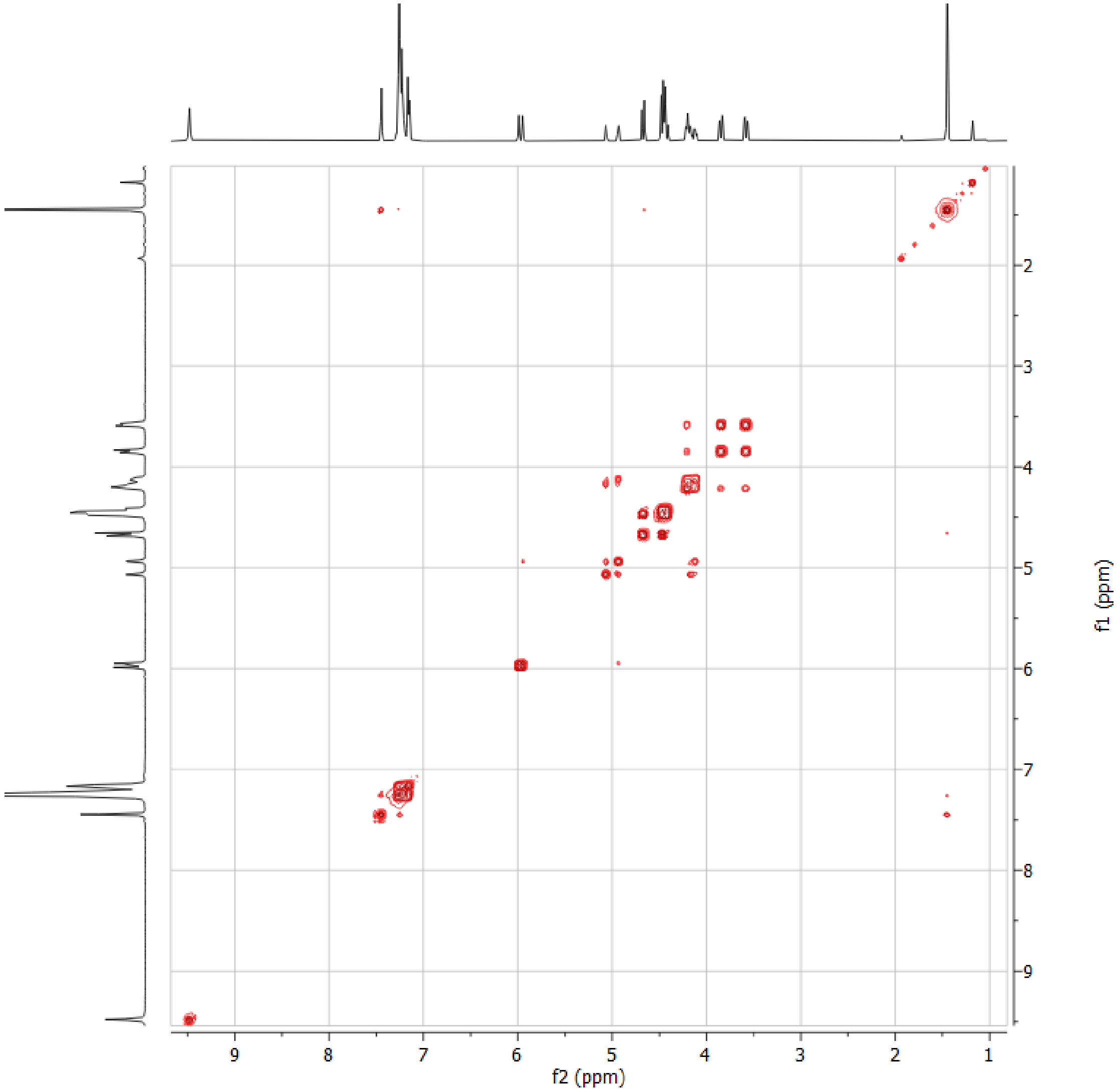

Extended Data 9. 2D-COSY spectrum of **4** (400 MHz, $CDCl_3$)

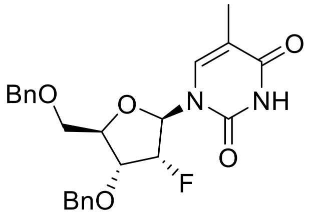

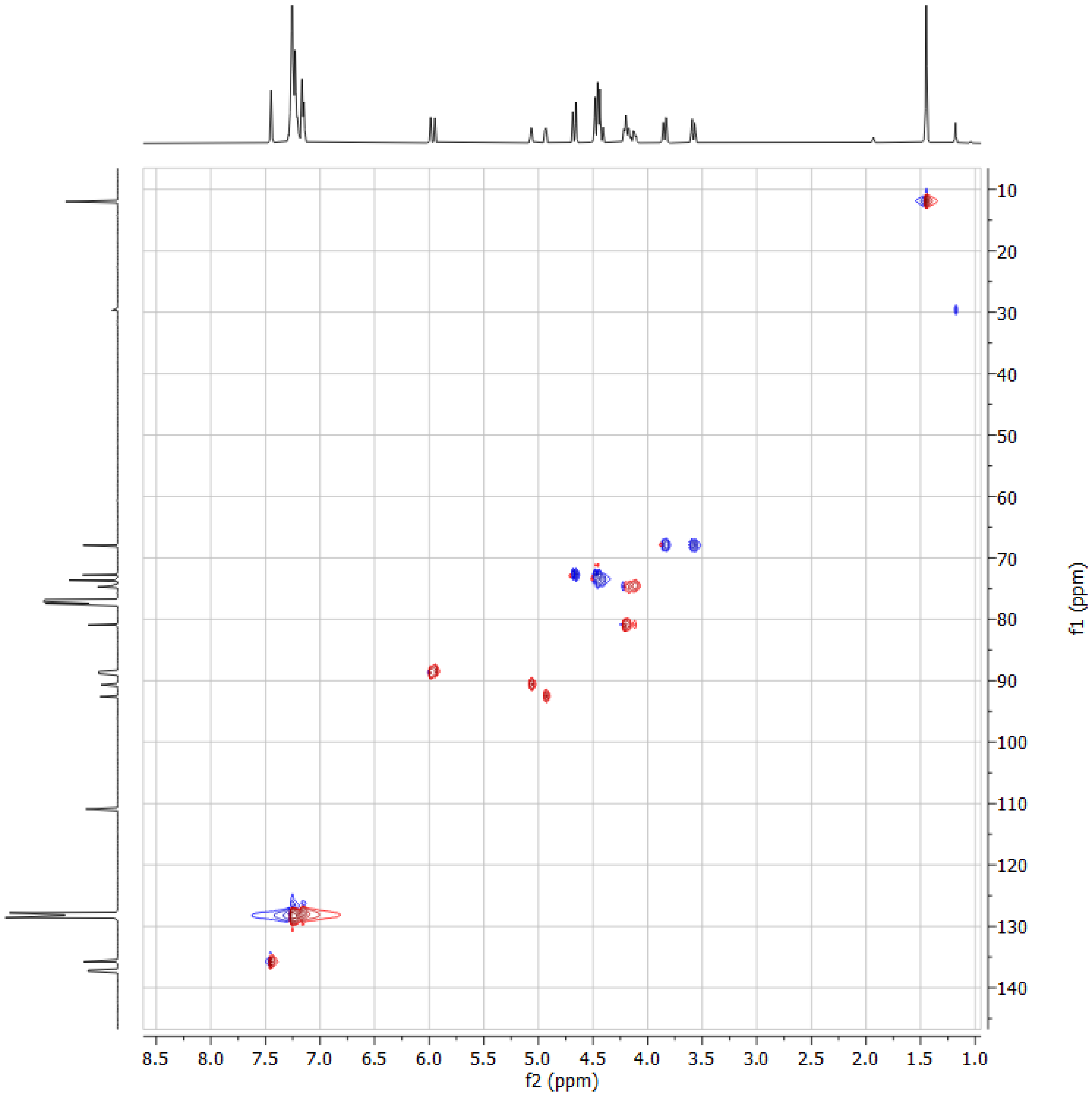

Extended Data 10. 2D-HSQC spectrum of **4** (400 MHz, $CDCl_3$)

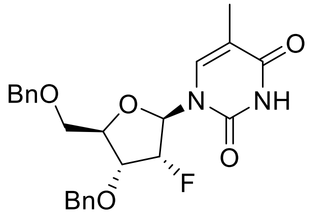

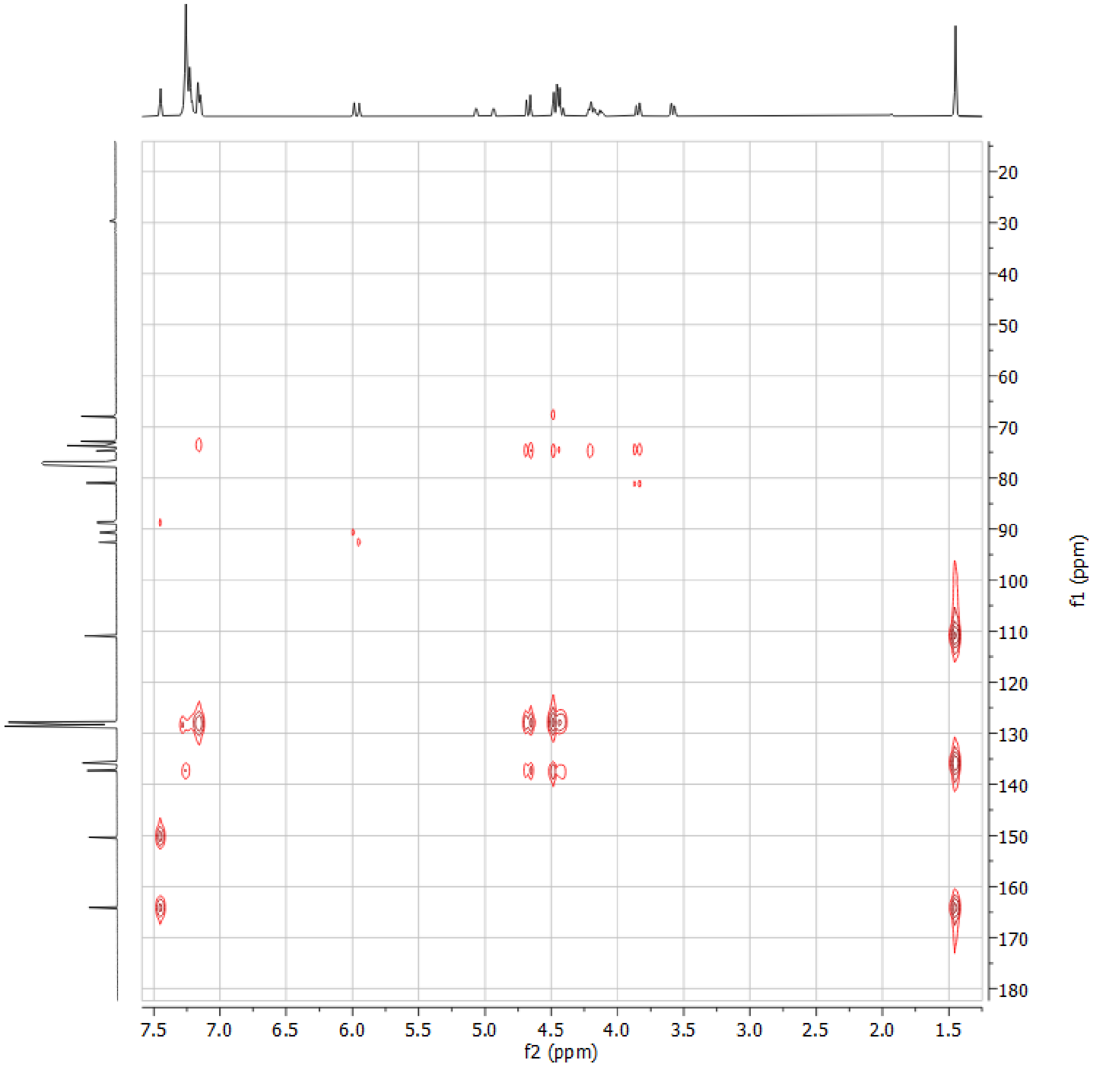

Extended Data 11. 2D-HMBC spectrum of **4** (400 MHz, $CDCl_3$)

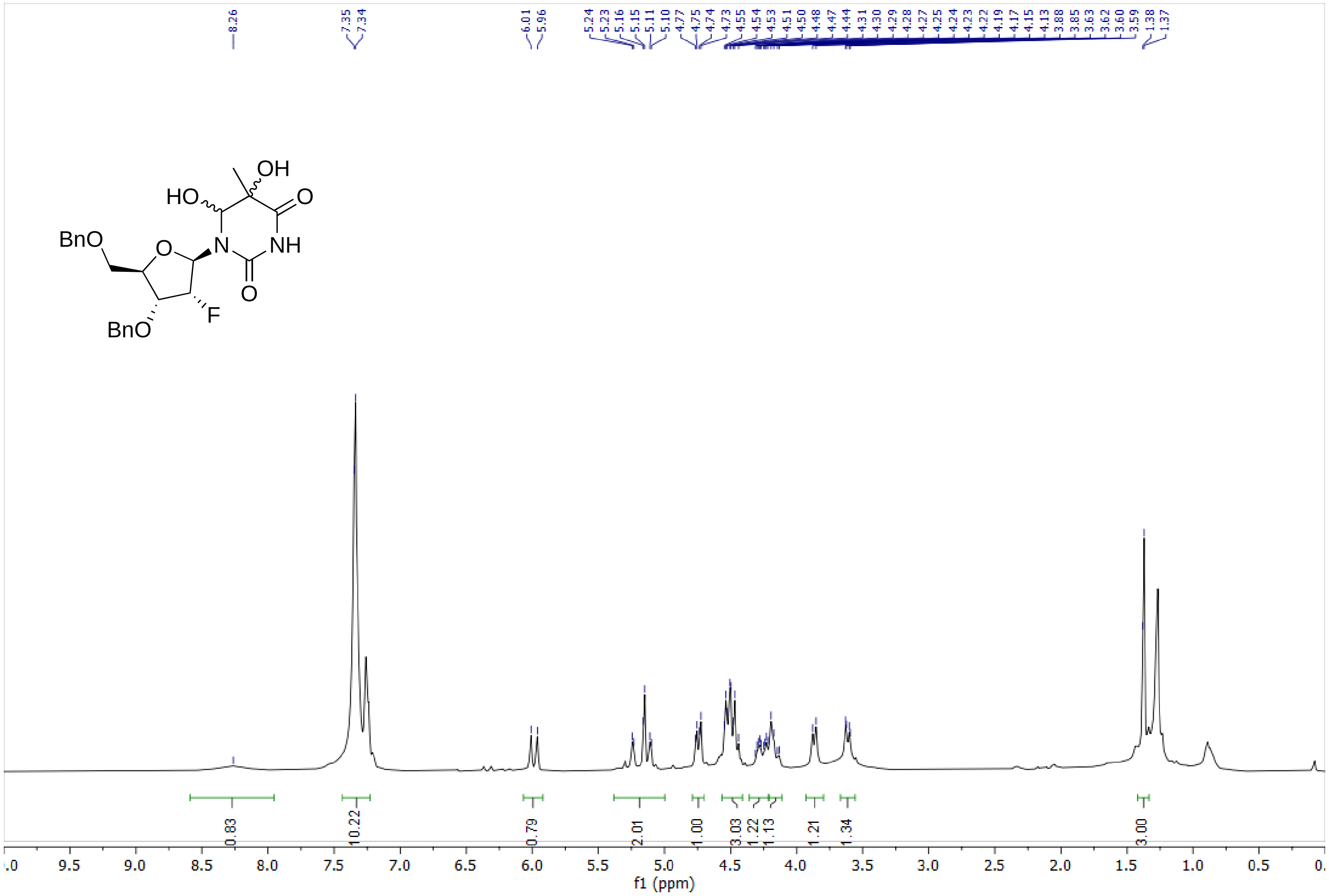

Extended Data 12. ^{1}H NMR spectrum of **5** (400 MHz $CDCl_3$)

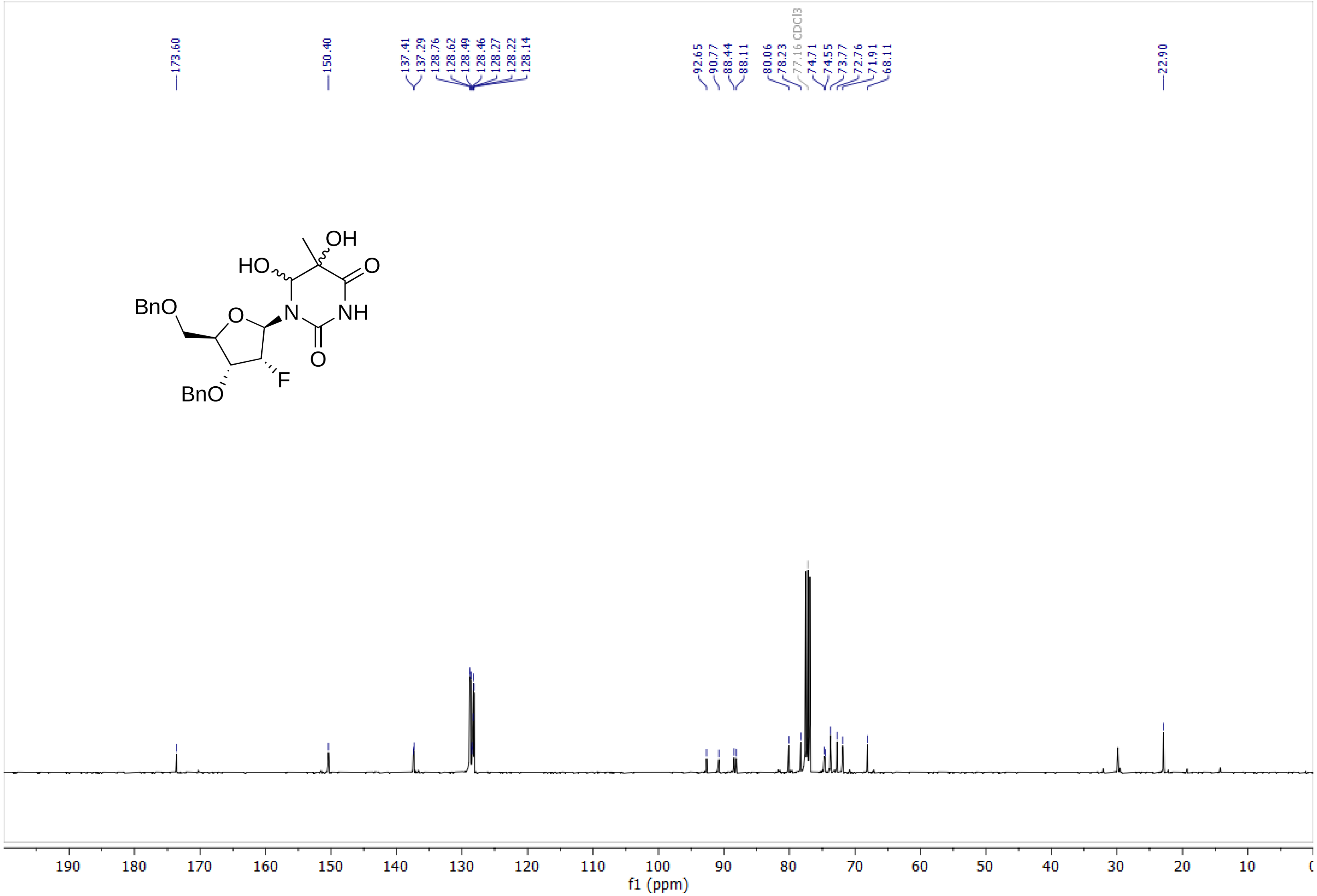

Extended Data 13. ^{13}C NMR spectrum of **5** (101 MHz, $CDCl_3$)

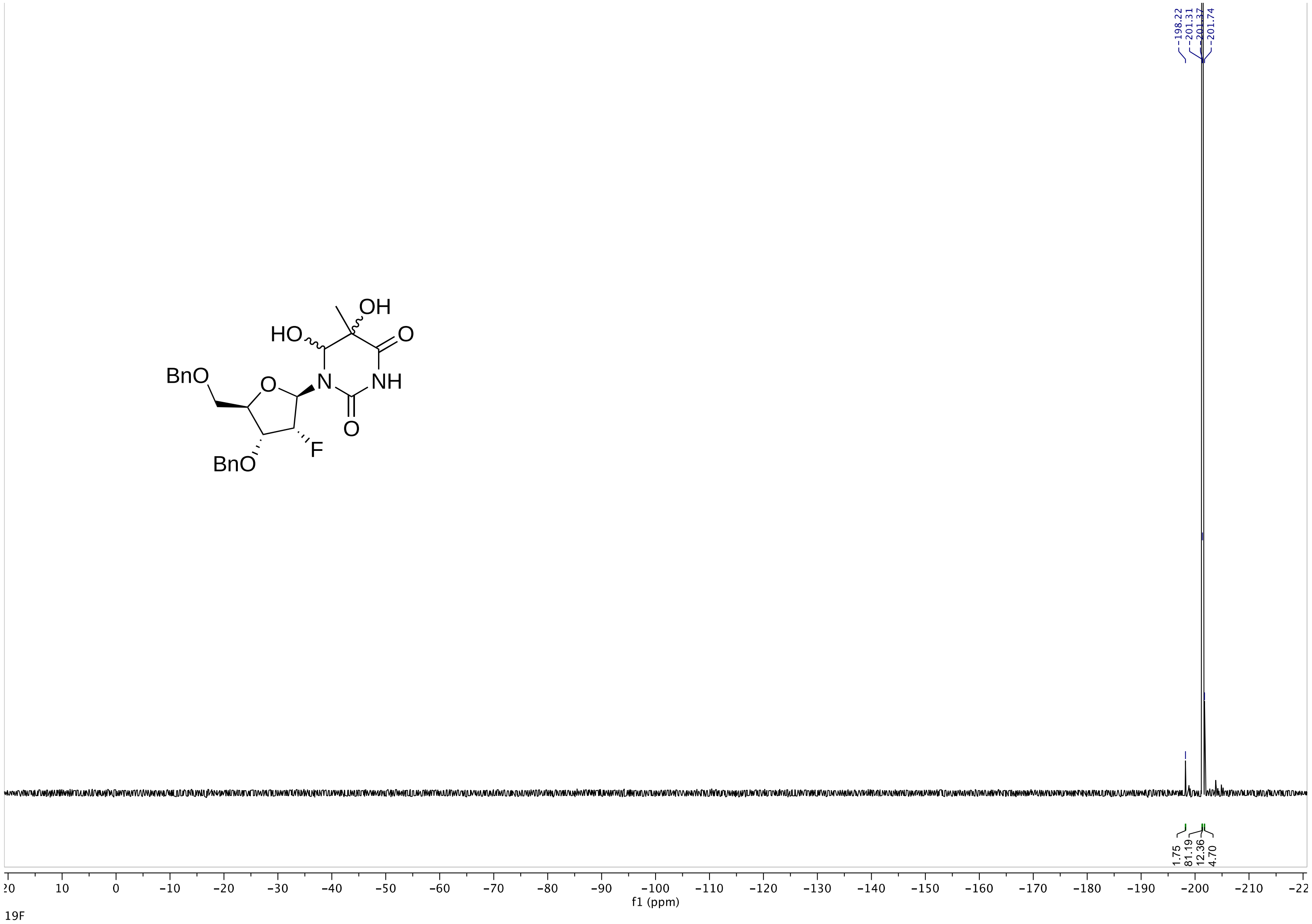

Extended Data 14. ^{19}F NMR spectrum of **5** (376 MHz, $CDCl_3$)

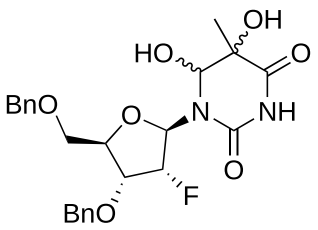

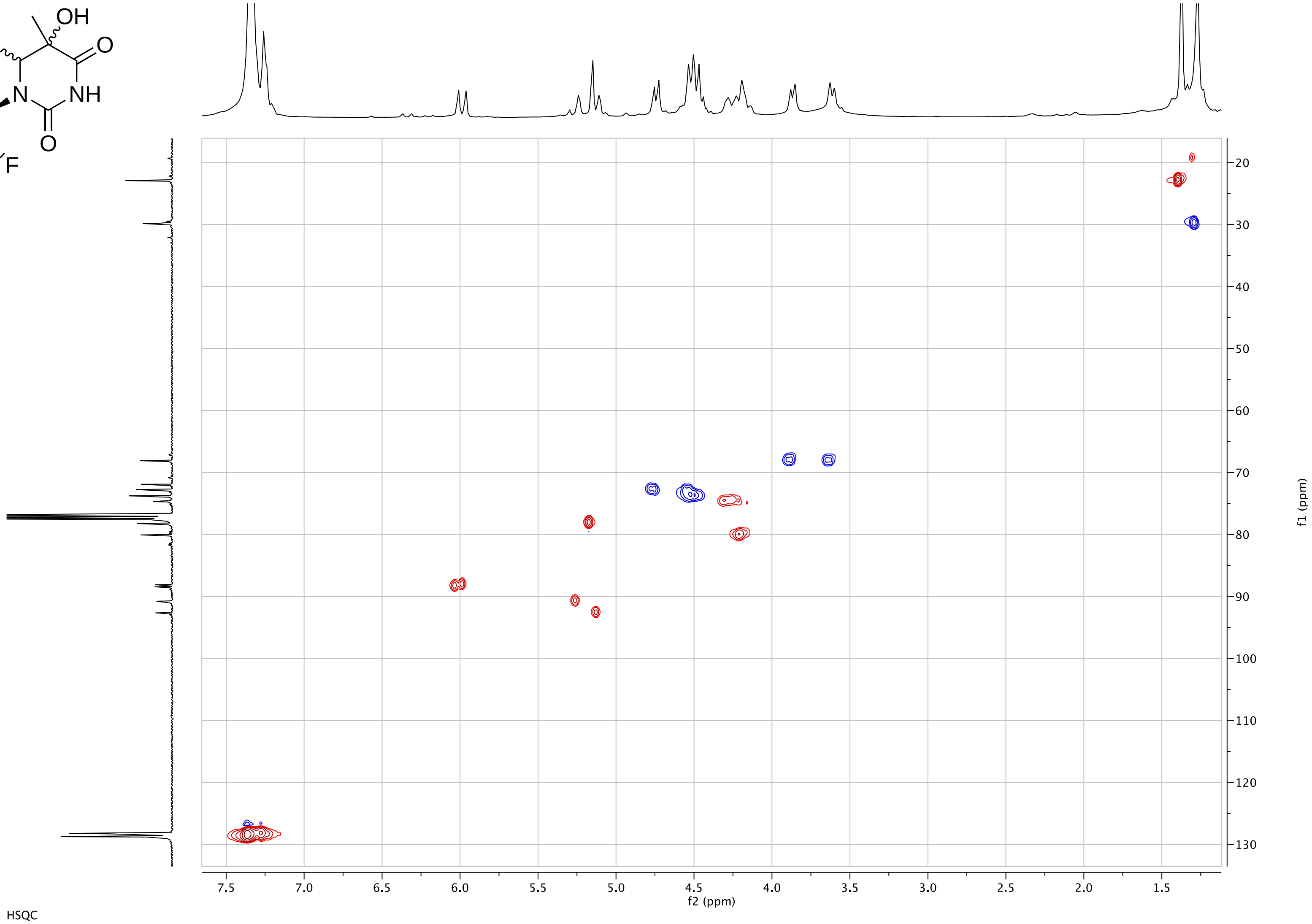

Extended Data 15. 2D-HSQC spectrum of **5** (400 MHz, $CDCl_3$)

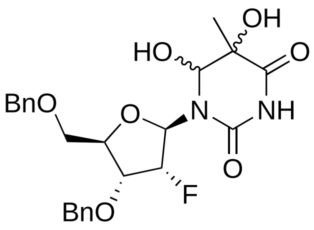

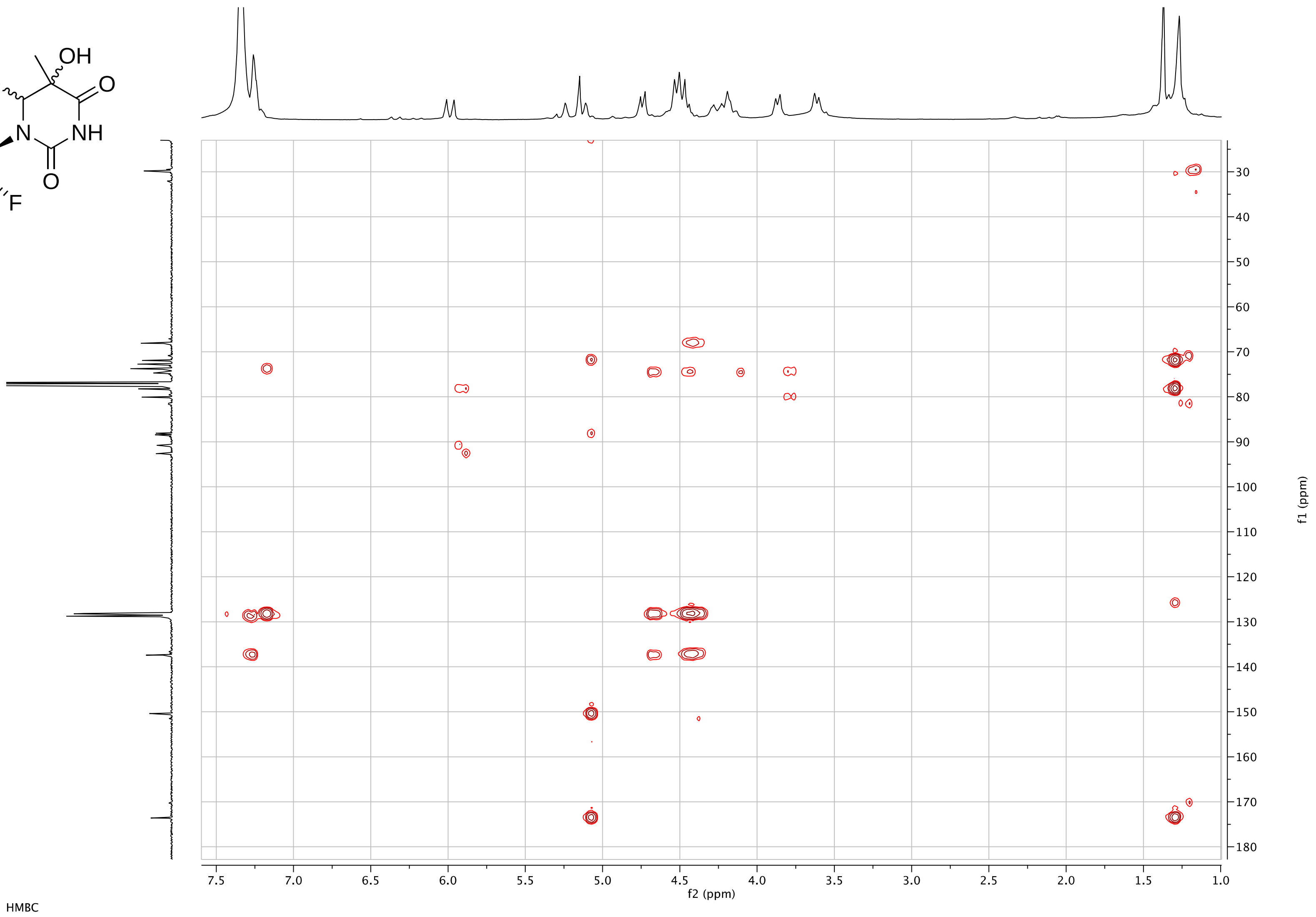

Extended Data 16. 2D HMBC spectrum of **5** (400 MHz, $CDCl_3$)

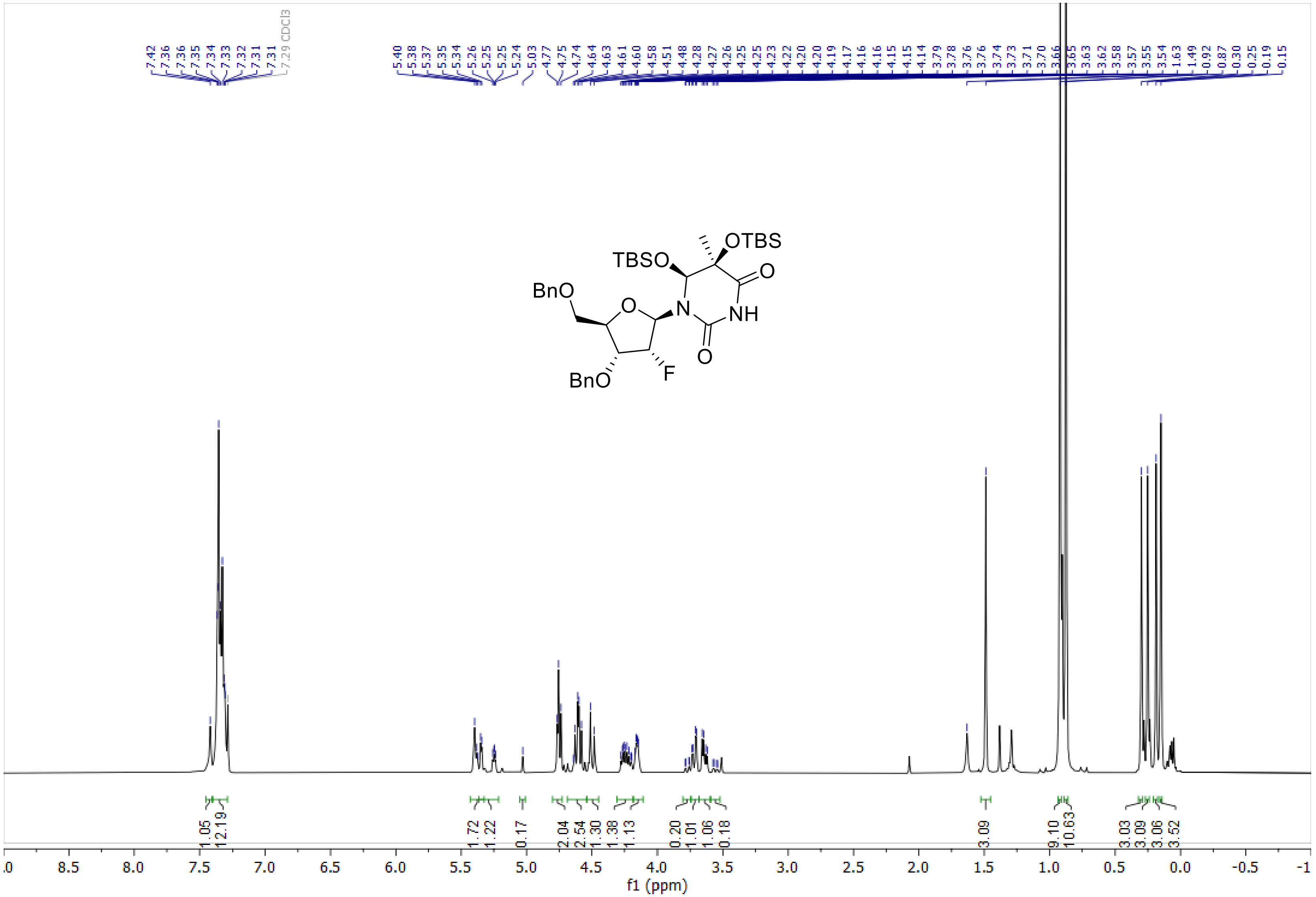

Extended Data 17. ^{1}H NMR spectrum of **6** (400 MHz CDCl₃)

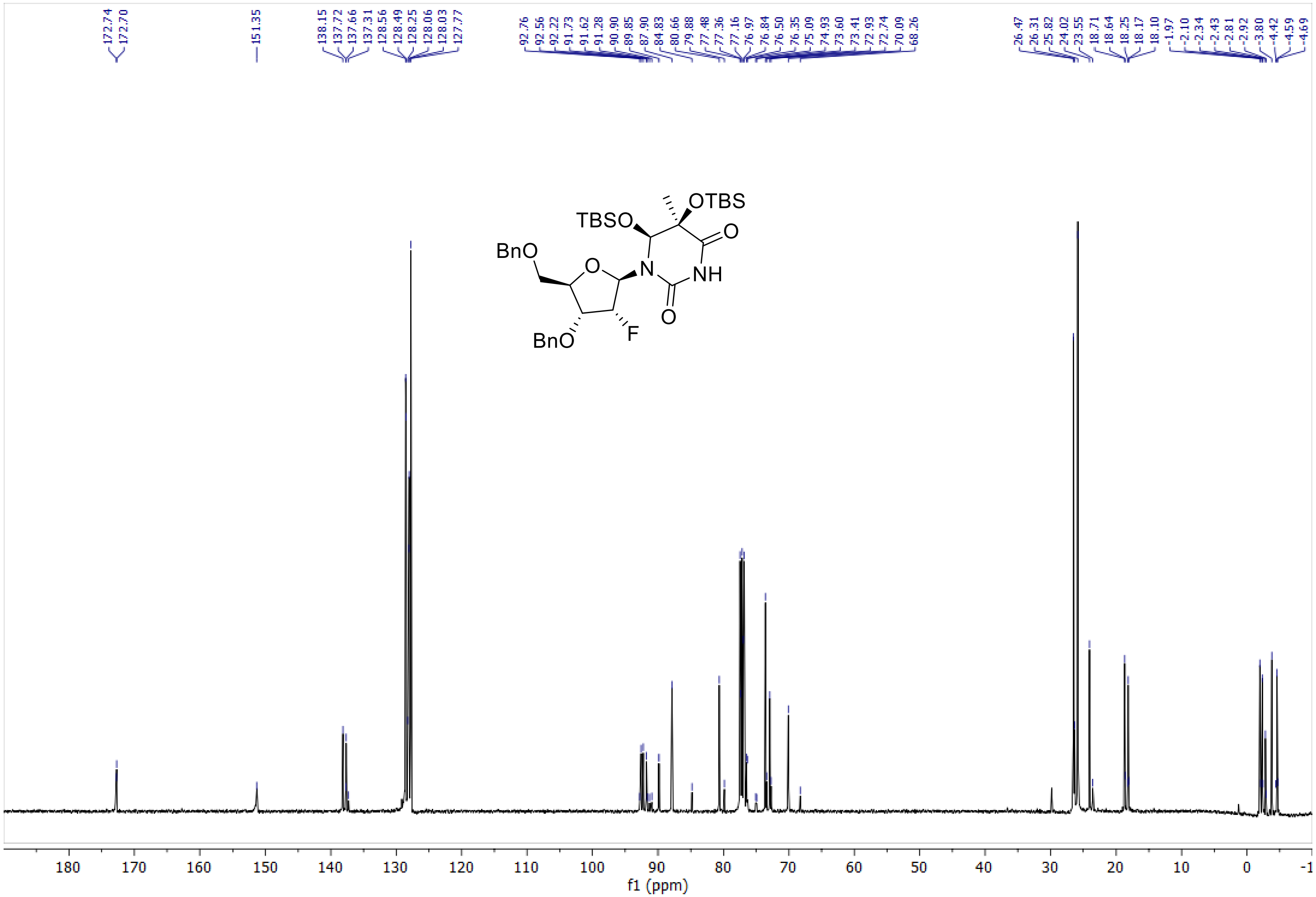

Extended Data 18. ^{13}C NMR spectrum of **6** (101 MHz $CDCl_3$)

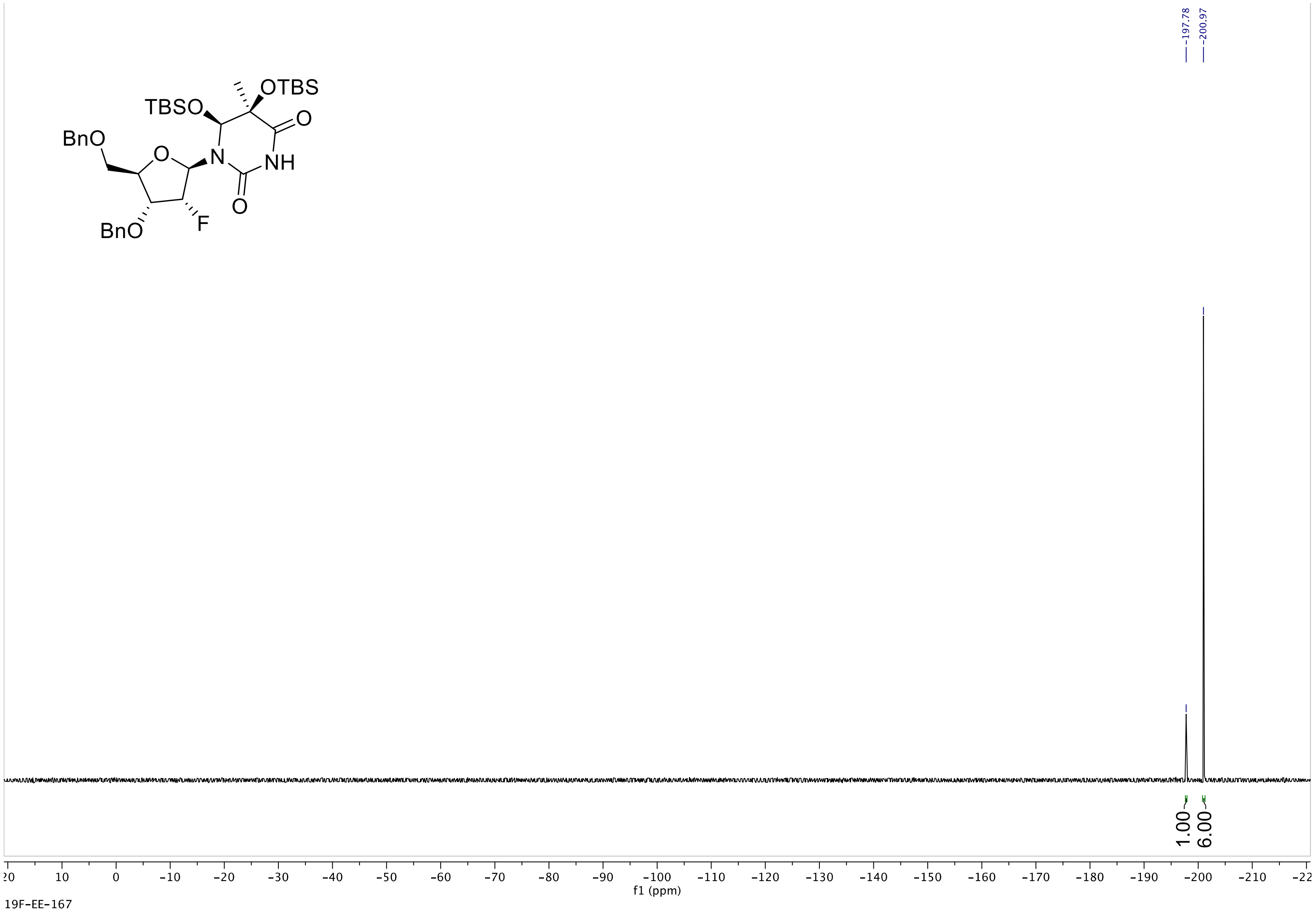

Extended Data 19. ^{19}F NMR spectrum of **6** (376 MHz, $CDCl_3$)

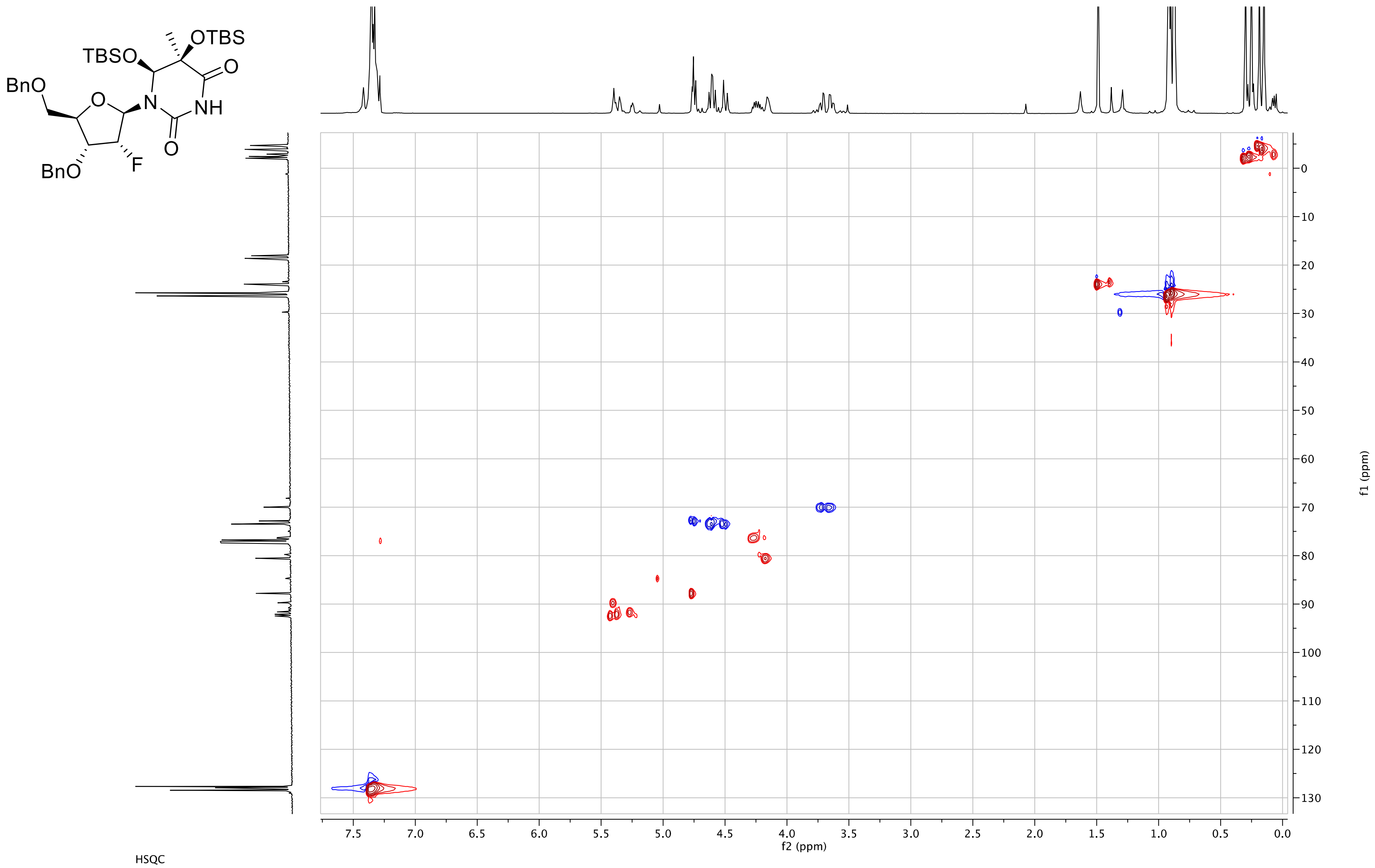

Extended Data 20. 2D -HSQC spectrum of **6** (400 MHz, $CDCl_3$)

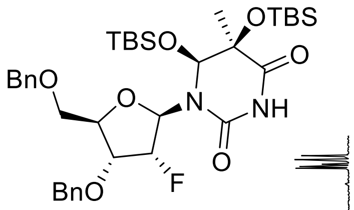

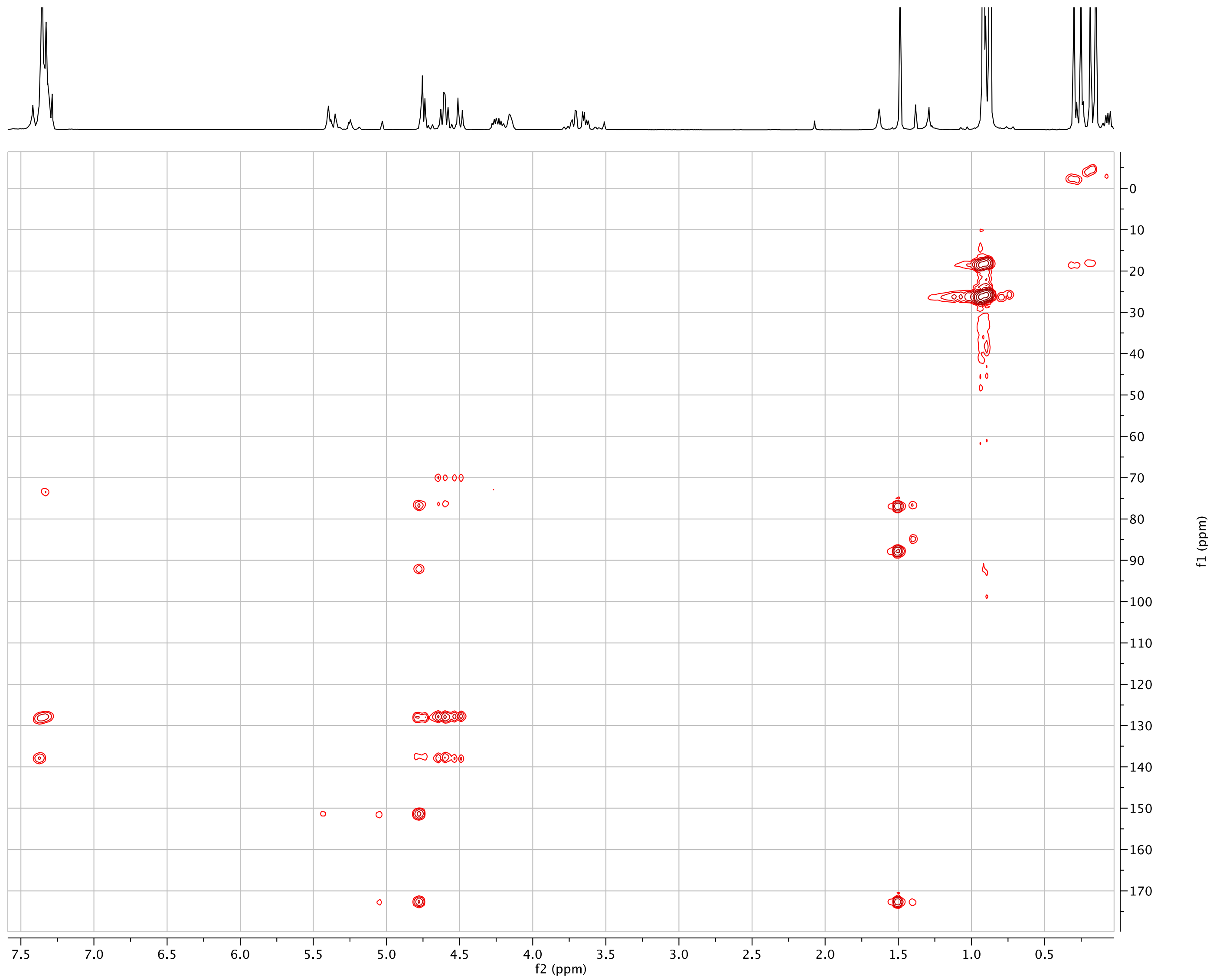

Extended Data 21. 2D -HMBC spectrum of **6** (400 MHz, $CDCl_3$)

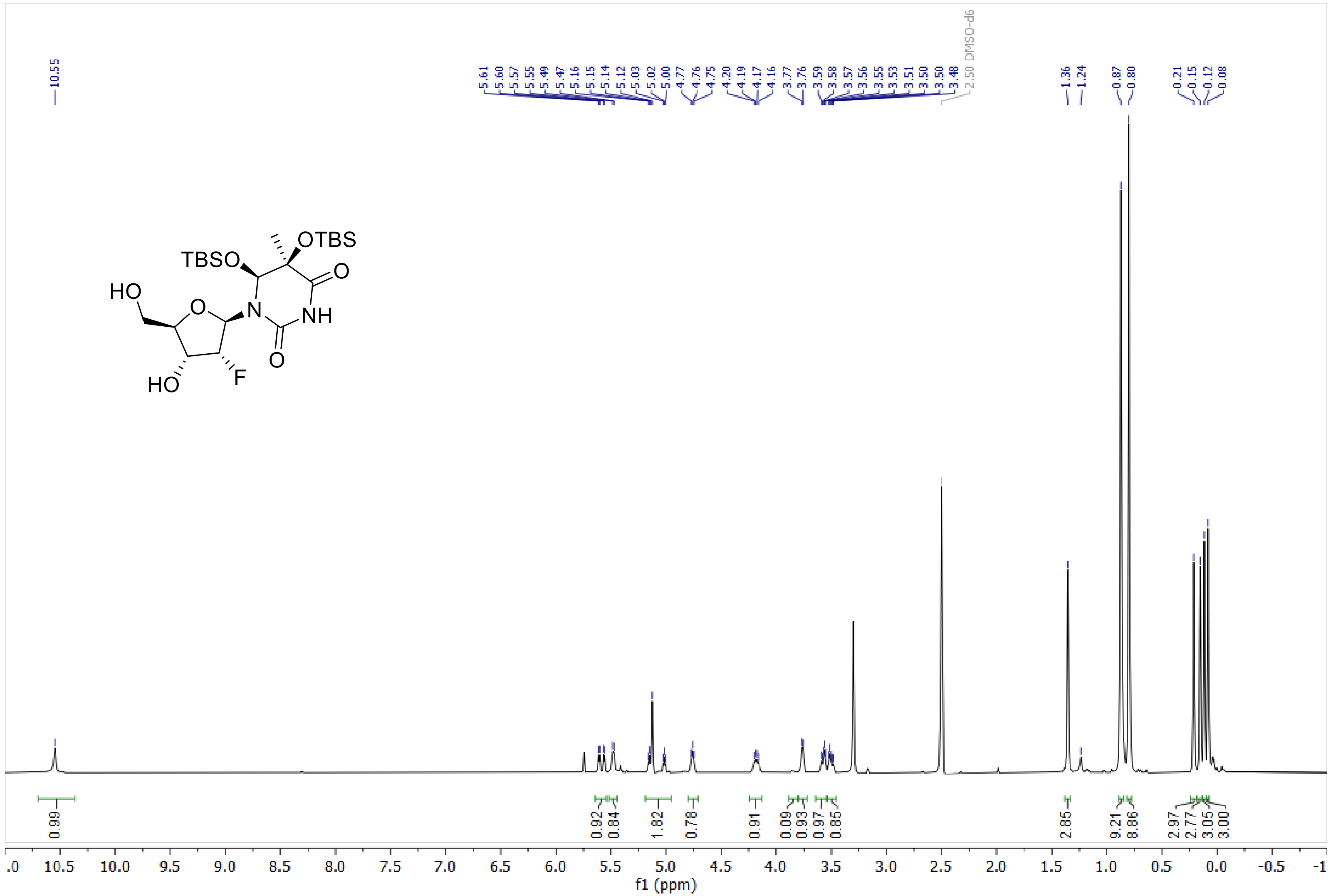

Extended Data 22. ^{1}H NMR spectrum of **7** (400 MHz $CDCl_3$)

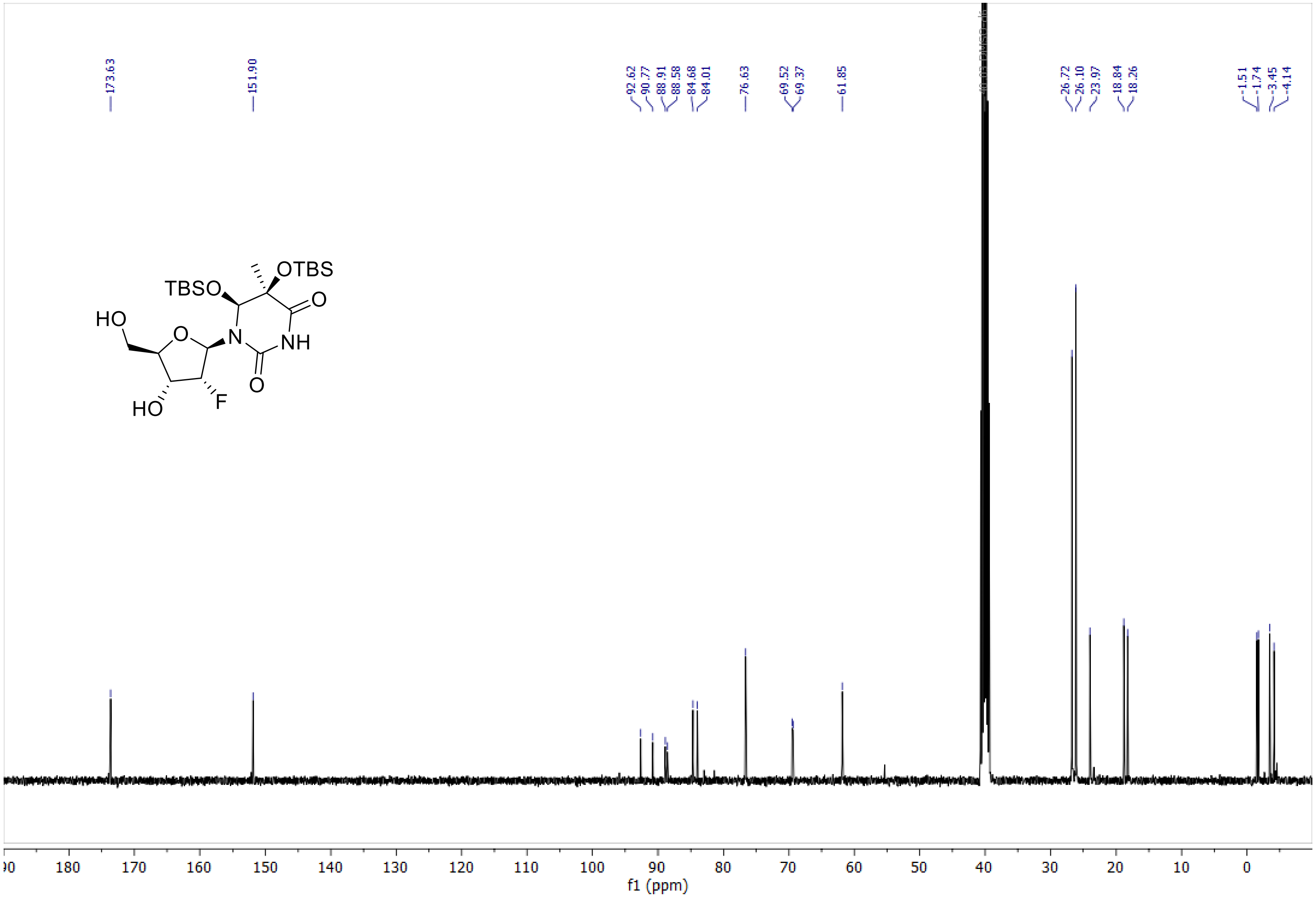

Extended Data 23. ^{13}C NMR spectrum of **7** (101 MHz $CDCl_3$)

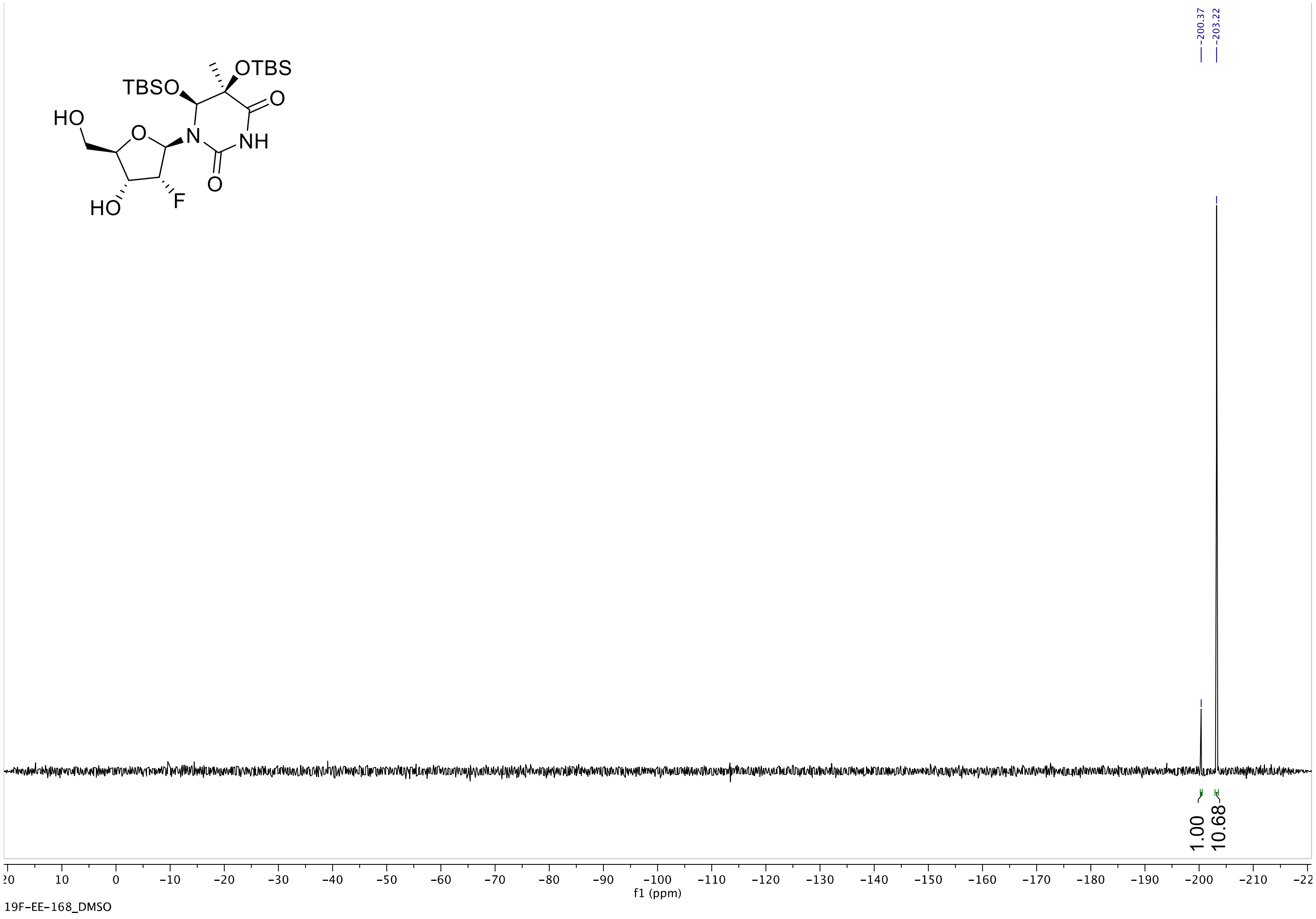

Extended Data 24. ^{19}F NMR spectrum of **7** (376 MHz, $CDCl_3$)

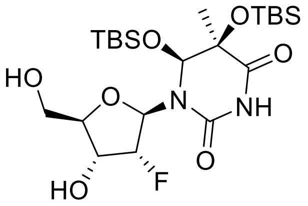

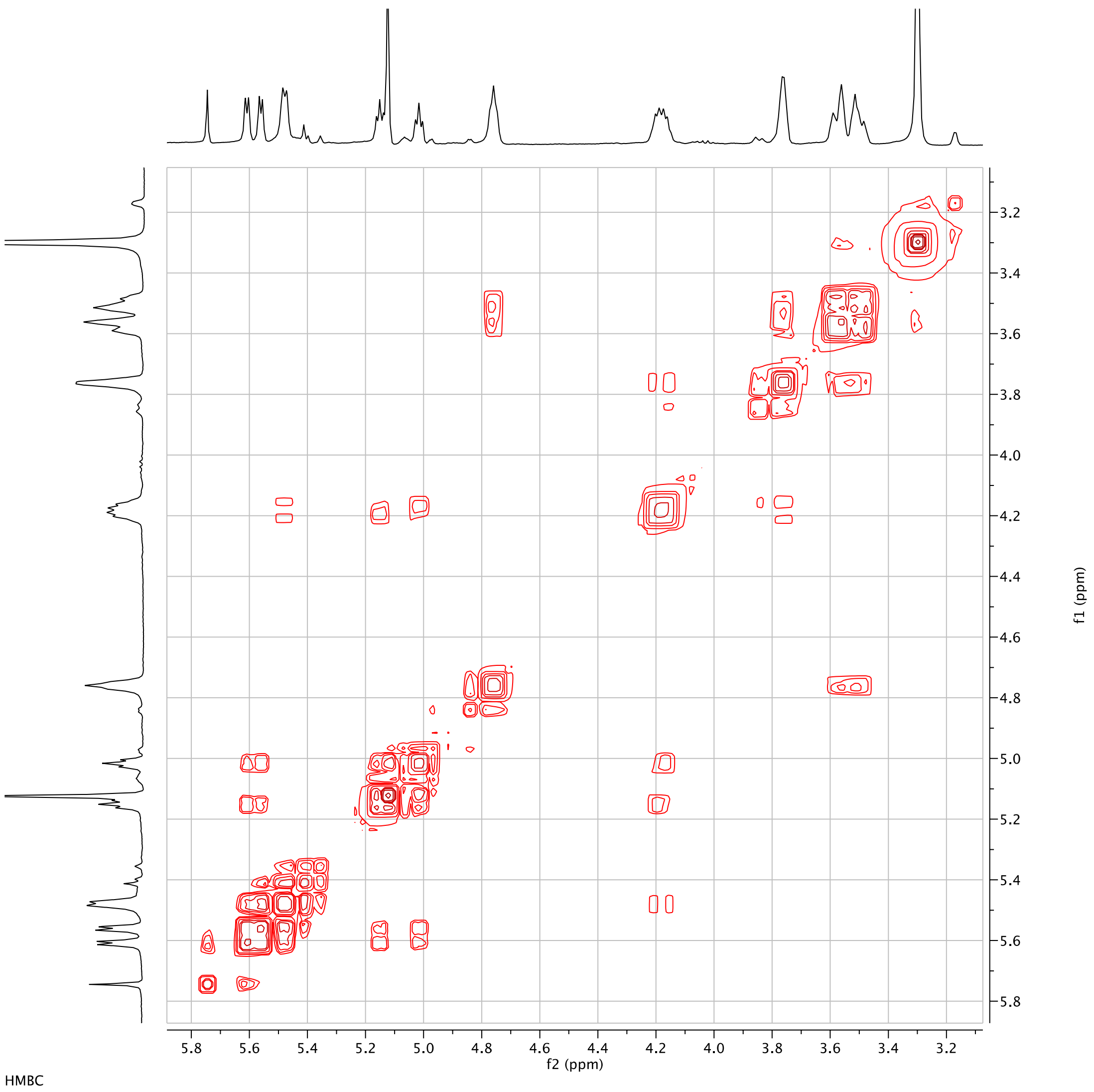

Extended Data 25. 2D-COSY spectrum of **7** (400 MHz, $CDCl_3$)

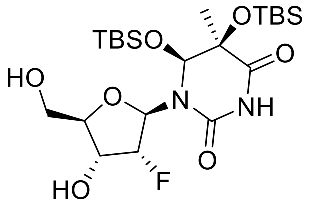

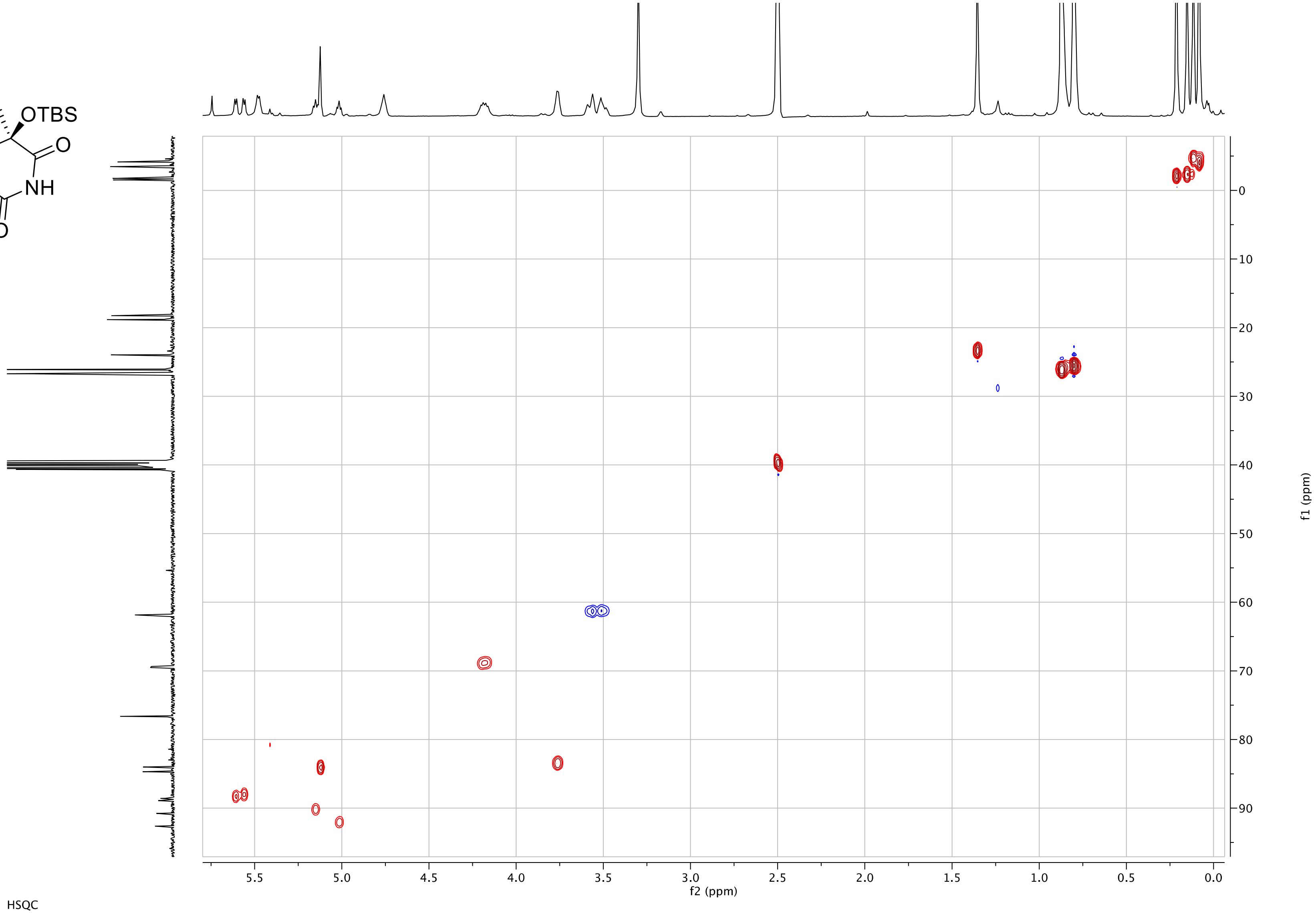

Extended Data 26. 2D -HSQC spectrum of **7** (400 MHz, $CDCl_3$)

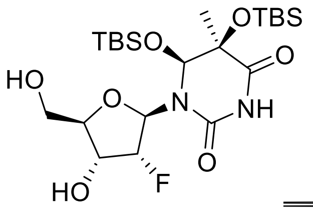

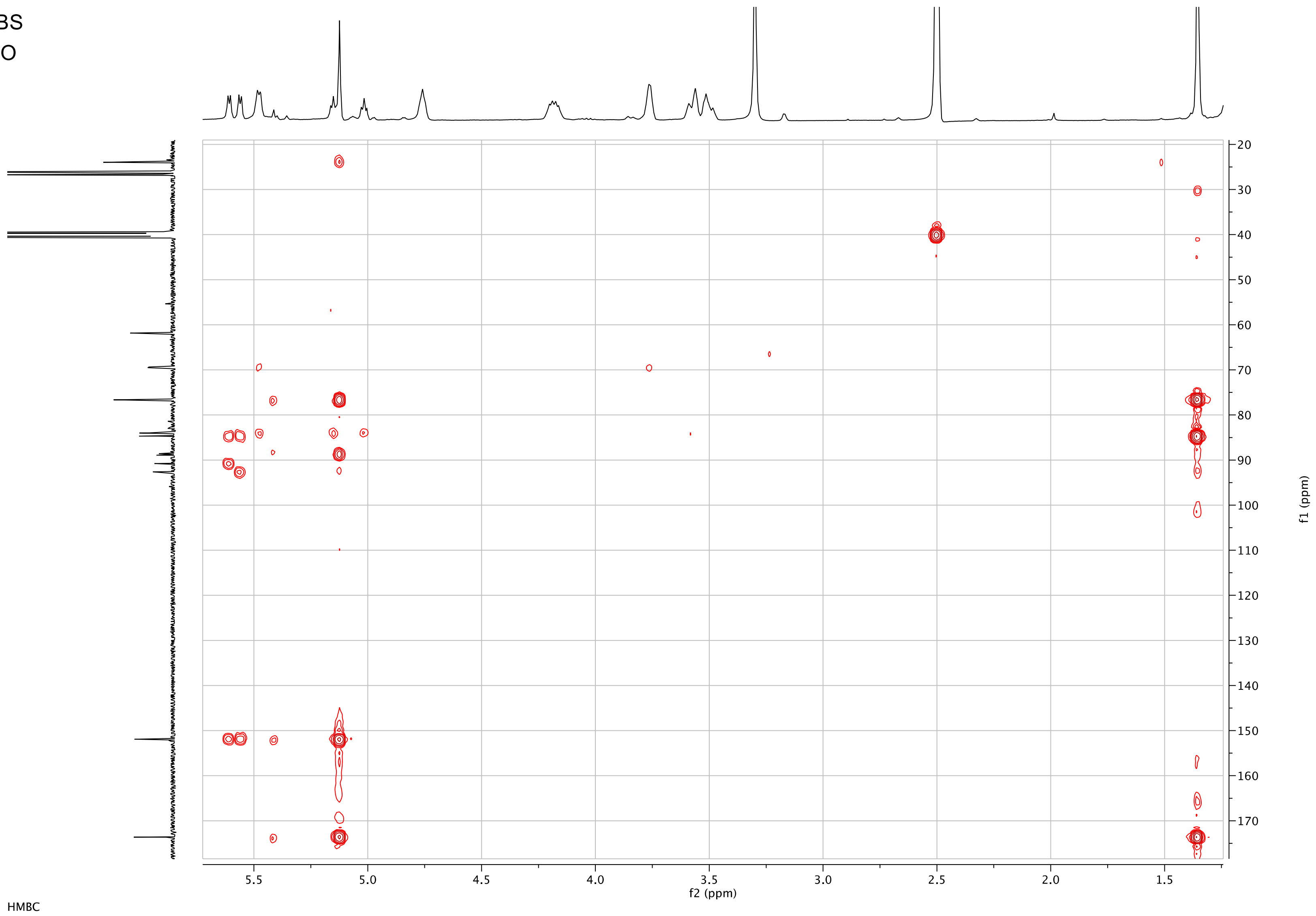

Extended Data 27. 2D -HMBC spectrum of **7** (400 MHz, $CDCl_3$)

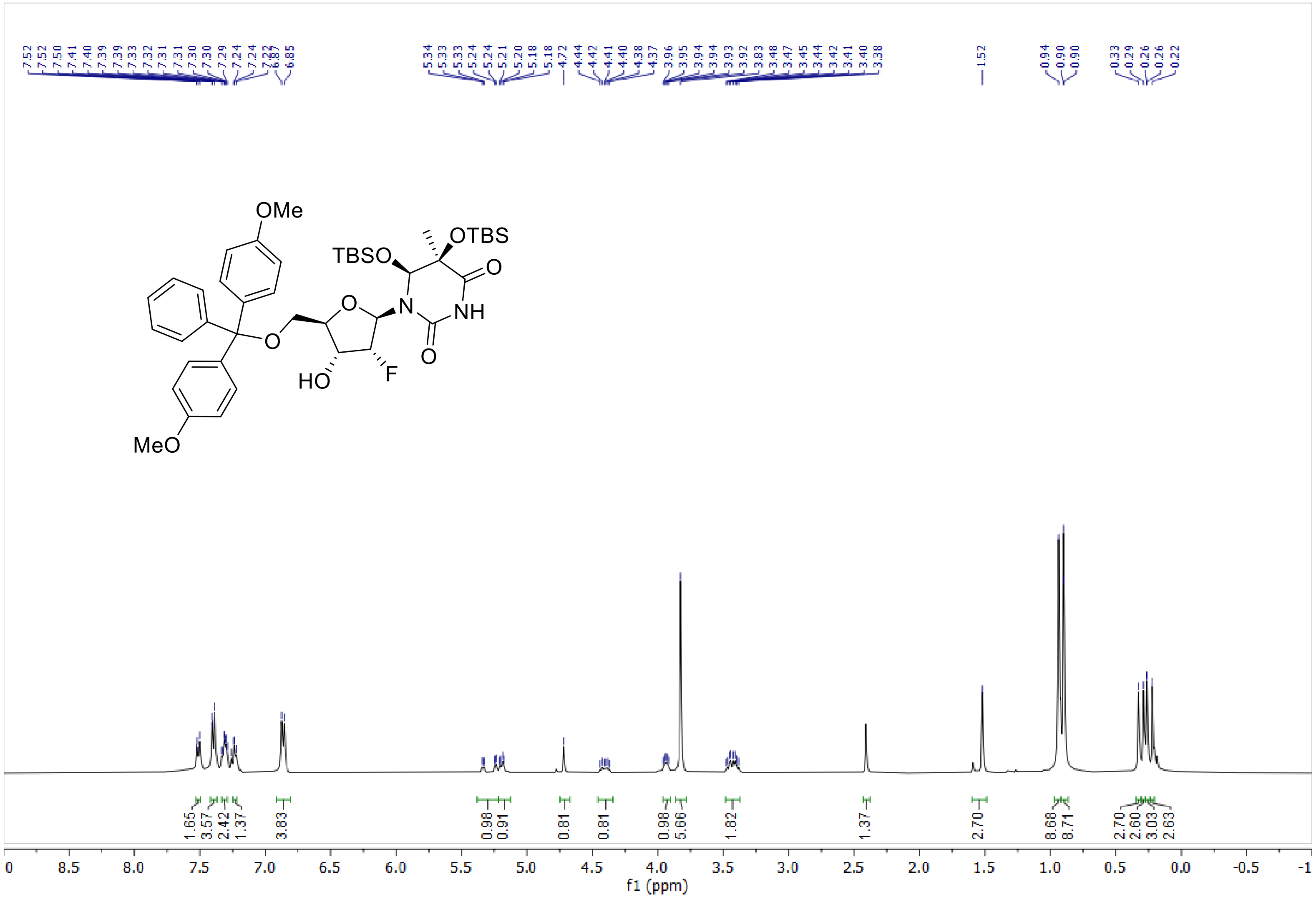

Extended Data 28. ^{1}H NMR spectrum of **8** (400 MHz $CDCl_3$)

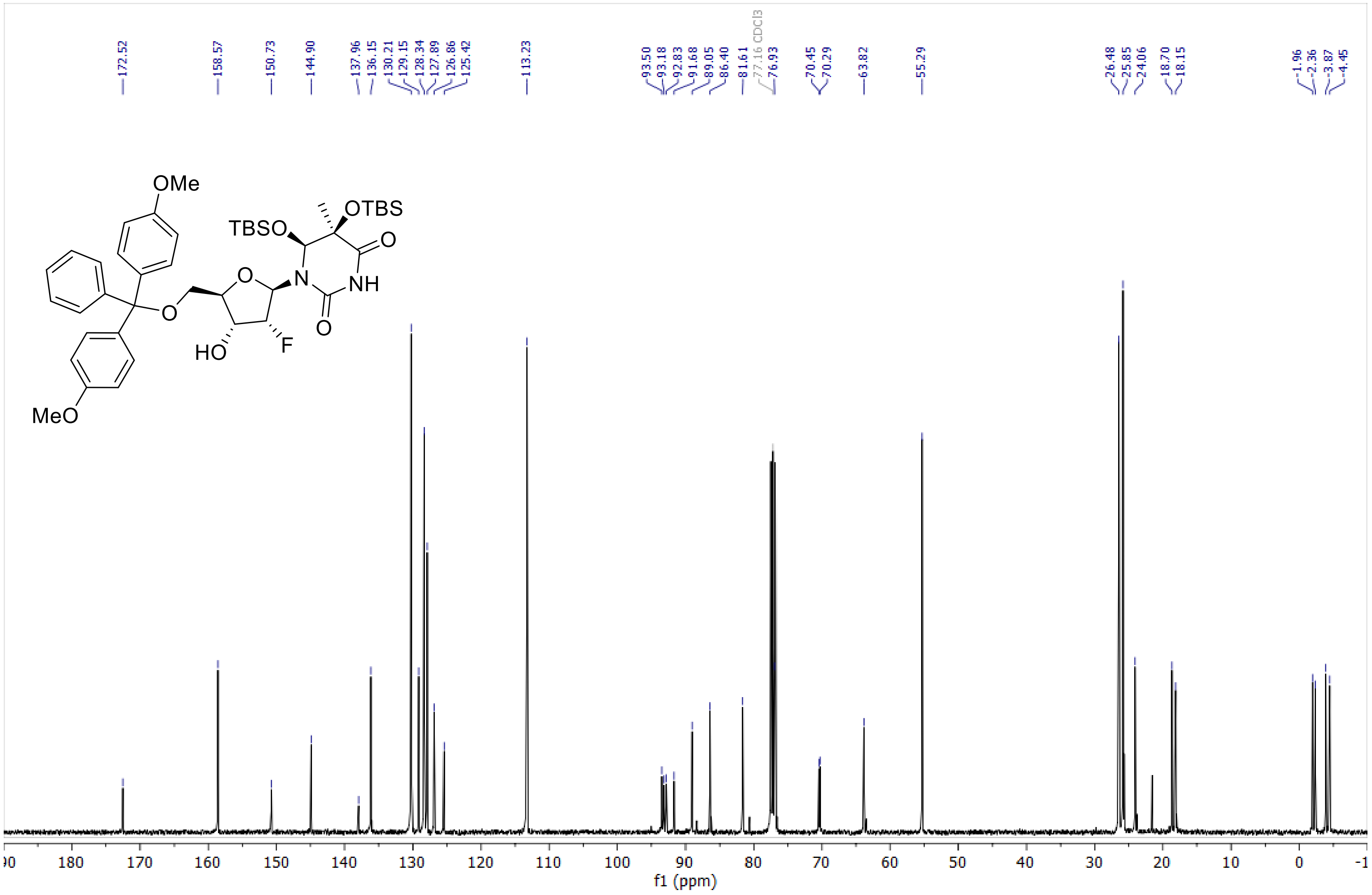

Extended Data 29. ^{13}C NMR spectrum of **8** (101 MHz $CDCl_3$)

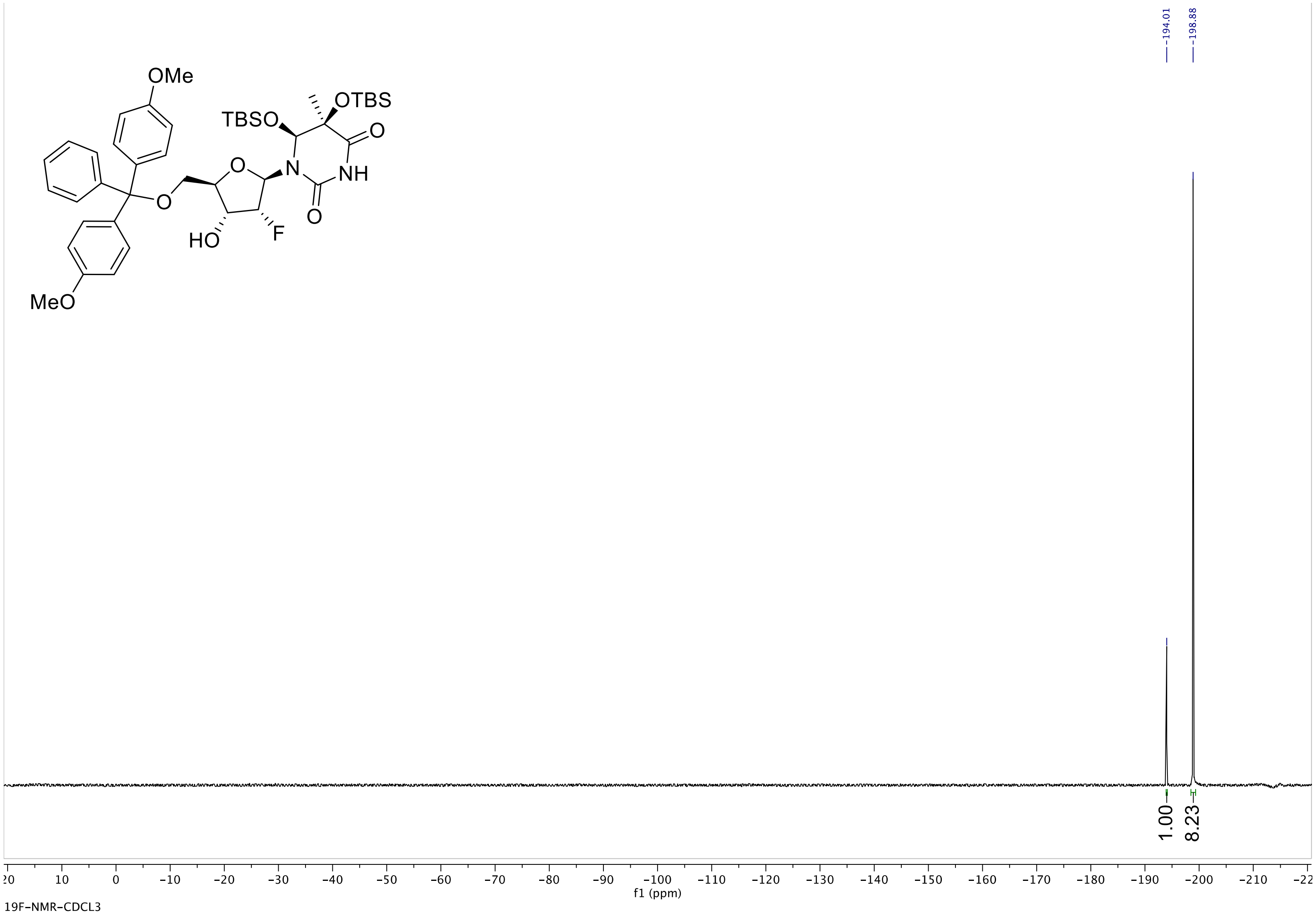

Extended Data 30. ^{19}F NMR spectrum of **8** (376 MHz, $CDCl_3$)

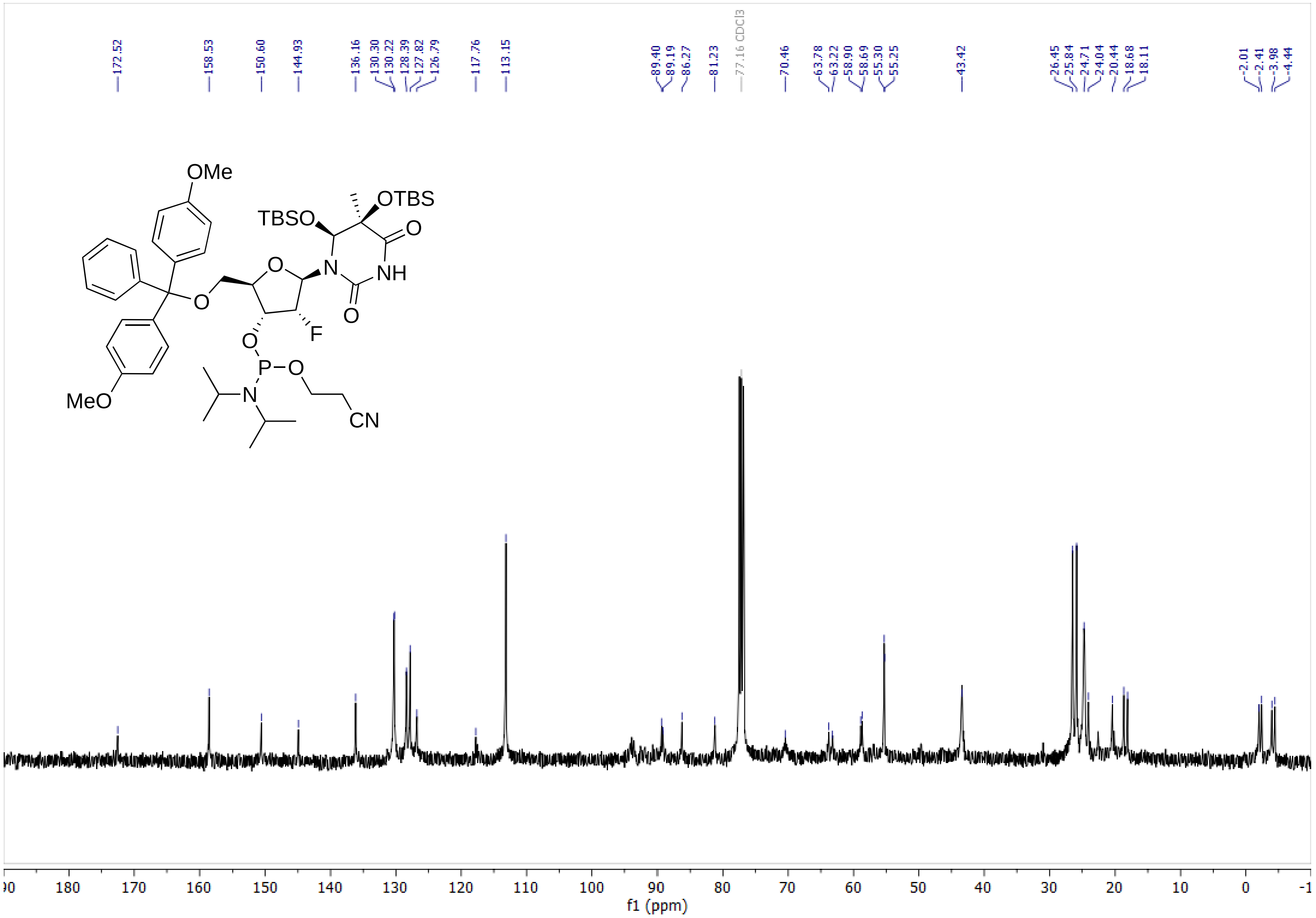

Extended Data 31. ^{13}C NMR spectrum of **9** (101 MHz $CDCl_3$)

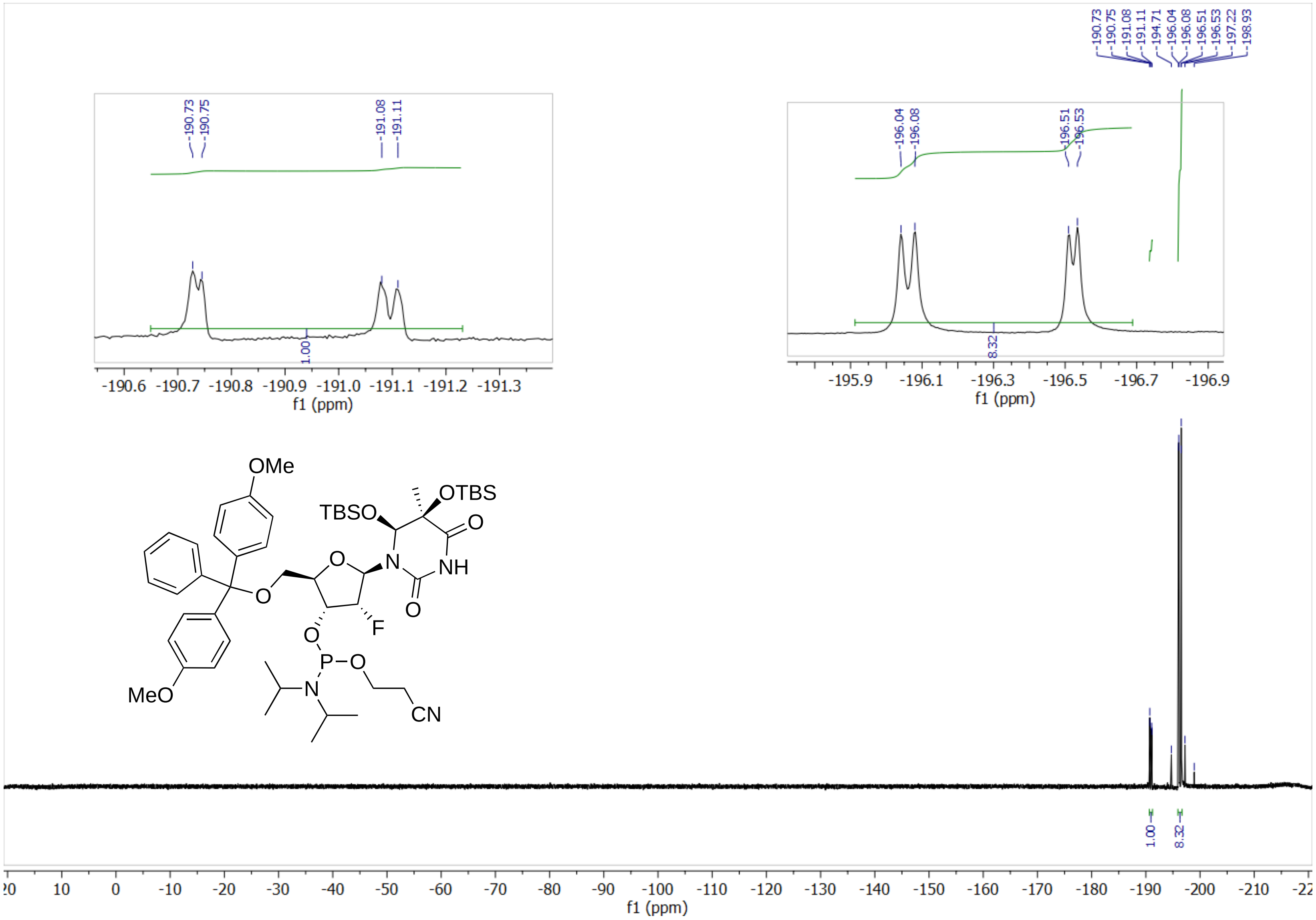

Extended Data 32. ^{19}F NMR spectrum of **9** (376 MHz, $CDCl_3$)

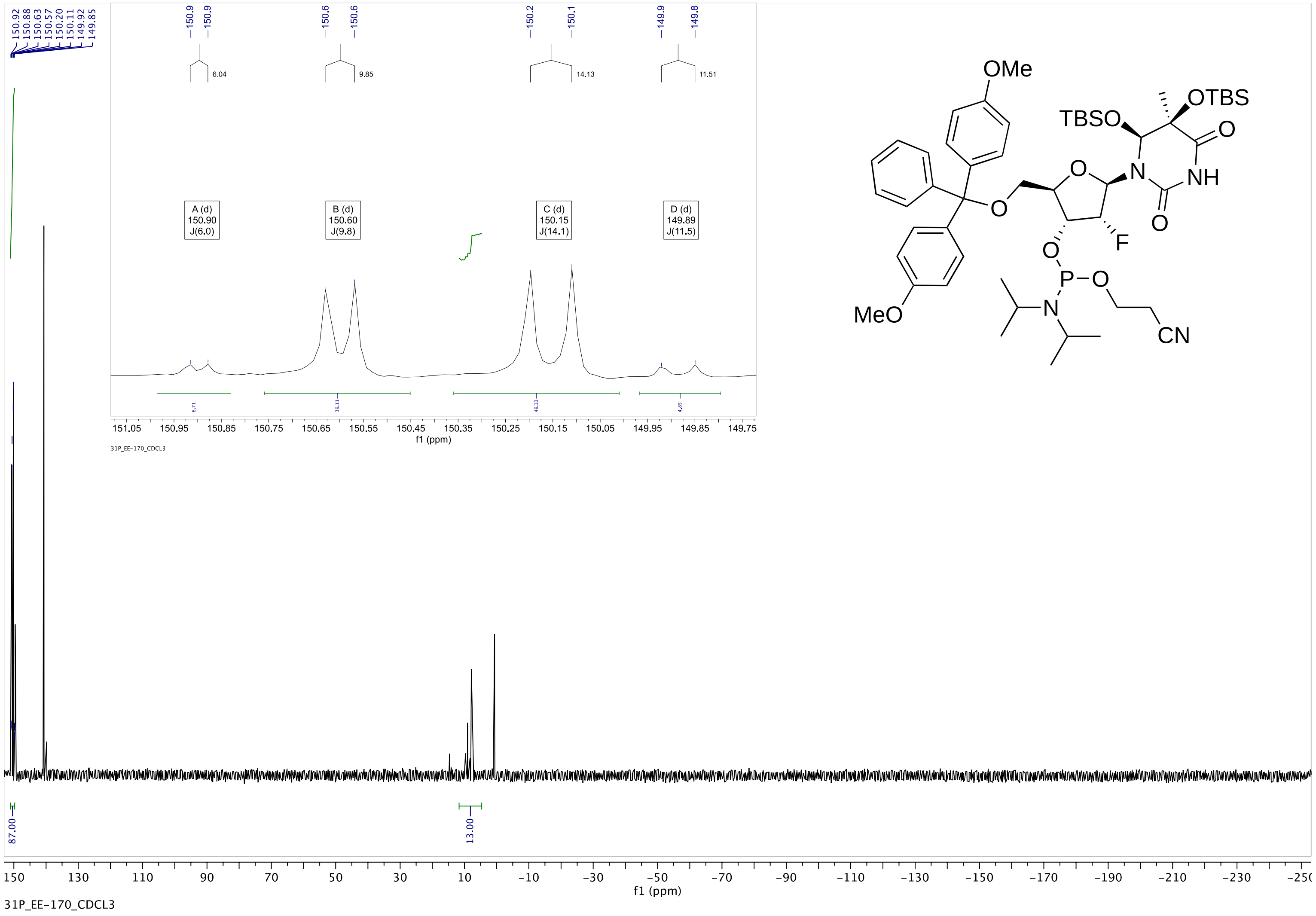

Extended Data 33. ^{31}P NMR spectrum of **9** (162 MHz, $CDCl_3$)

Supplementary References

1. Ikeda, H., Fernandez, R., Wilk, A., Barchi, J.J., Jr., Huang, X. and Marquez, V.E. (1998) The effect of two antipodal fluorine-induced sugar puckers on the conformation and stability of the Dickerson-Drew dodecamer duplex [d(CGCGAATTCGCG)]2. Nucleic Acids Res., 26, 2237-2244.
2. Liu, P., Sharon, A. and Chu, C.K. (2008) Fluorinated Nucleosides: Synthesis and Biological Implication. J Fluor Chem, 129, 743-766.
3. Doi, Y., Katafuchi, A., Fujiwara, Y., Hitomi, K., Tainer, J.A., Ide, H. and Iwai, S. (2006) Synthesis and characterization of oligonucleotides containing 2'-fluorinated thymidine glycol as inhibitors of the endonuclease III reaction. Nucleic Acids Res., 34, 1540-1551.
4. Kitsera, N., Gasteiger, K., Luhnsdorf, B., Allgayer, J., Epe, B., Carell, T. and Khobta, A. (2014) Cockayne syndrome: varied requirement of transcription-coupled nucleotide excision repair for the removal of three structurally different adducts from transcribed DNA. PLoS One, 9, e94405.
